# New Susceptibility Loci Associated with Kidney Disease in Type 1 Diabetes

**DOI:** 10.1371/journal.pgen.1002921

**Published:** 2012-09-20

**Authors:** Niina Sandholm, Rany M. Salem, Amy Jayne McKnight, Eoin P. Brennan, Carol Forsblom, Tamara Isakova, Gareth J. McKay, Winfred W. Williams, Denise M. Sadlier, Ville-Petteri Mäkinen, Elizabeth J. Swan, Cameron Palmer, Andrew P. Boright, Emma Ahlqvist, Harshal A. Deshmukh, Benjamin J. Keller, Huateng Huang, Aila J. Ahola, Emma Fagerholm, Daniel Gordin, Valma Harjutsalo, Bing He, Outi Heikkilä, Kustaa Hietala, Janne Kytö, Päivi Lahermo, Markku Lehto, Raija Lithovius, Anne-May Österholm, Maija Parkkonen, Janne Pitkäniemi, Milla Rosengård-Bärlund, Markku Saraheimo, Cinzia Sarti, Jenny Söderlund, Aino Soro-Paavonen, Anna Syreeni, Lena M. Thorn, Heikki Tikkanen, Nina Tolonen, Karl Tryggvason, Jaakko Tuomilehto, Johan Wadén, Geoffrey V. Gill, Sarah Prior, Candace Guiducci, Daniel B. Mirel, Andrew Taylor, S. Mohsen Hosseini, DCCT/EDIC Research Group, Hans-Henrik Parving, Peter Rossing, Lise Tarnow, Claes Ladenvall, François Alhenc-Gelas, Pierre Lefebvre, Vincent Rigalleau, Ronan Roussel, David-Alexandre Tregouet, Anna Maestroni, Silvia Maestroni, Henrik Falhammar, Tianwei Gu, Anna Möllsten, Danut Cimponeriu, Mihai Ioana, Maria Mota, Eugen Mota, Cristian Serafinceanu, Monica Stavarachi, Robert L. Hanson, Robert G. Nelson, Matthias Kretzler, Helen M. Colhoun, Nicolae Mircea Panduru, Harvest F. Gu, Kerstin Brismar, Gianpaolo Zerbini, Samy Hadjadj, Michel Marre, Leif Groop, Maria Lajer, Shelley B. Bull, Daryl Waggott, Andrew D. Paterson, David A. Savage, Stephen C. Bain, Finian Martin, Joel N. Hirschhorn, Catherine Godson, Jose C. Florez, Per-Henrik Groop, Alexander P. Maxwell

**Affiliations:** 1 Folkhälsan Institute of Genetics, Folkhälsan Research Center, Biomedicum Helsinki, Helsinki, Finland; 2 Division of Nephrology, Department of Medicine, Helsinki University Central Hospital, Helsinki, Finland; 3 Department of Biomedical Engineering and Computational Science, Aalto University, Espoo, Finland; 4 Program in Medical and Population Genetics, Broad Institute, Cambridge, Massachusetts, United States of America; 5 Endocrine Research Unit, Department of Endocrinology, Children's Hospital, Boston, Massachusetts, United States of America; 6 Department of Medicine, Harvard Medical School, Boston, Massachusetts, United States of America; 7 Nephrology Research, Centre for Public Health, Queen's University of Belfast, Belfast, United Kingdom; 8 Diabetes Research Centre, Conway Institute, School of Medicine and Medical Sciences, University College Dublin, Dublin, Ireland; 9 Mater Misericordiae Hospital, Dublin, Ireland; 10 Division of Nephrology and Hypertension, University of Miami, Miami, Florida, United States of America; 11 Center for Human Genetic Research, Massachusetts General Hospital, Boston, Massachusetts, United States of America; 12 Institute of Clinical Medicine, Department of Internal Medicine, Biocenter Oulu and Clinical Research Center, University of Oulu, Oulu, Finland; 13 Department of Medicine, University of Toronto, Toronto, Canada; 14 Department of Clinical Sciences, Diabetes, and Endocrinology, Skåne University Hospital, Lund University, Malmö, Sweden; 15 Wellcome Trust Centre for Molecular Medicine, University of Dundee, Dundee, Scotland, United Kingdom; 16 Computer Science, Eastern Michigan University, Ypsilanti, Michigan, United States of America; 17 Division of Nephrology, Internal Medicine, University of Michigan, Ann Arbor, Michigan, United States of America; 18 Diabetes Prevention Unit, National Institute for Health and Welfare, Helsinki, Finland; 19 Division of Matrix Biology, Department of Medical Biochemistry and Biophysics, Karolinska Institutet, Stockholm, Sweden; 20 Department of Ophthalmology, Helsinki University Central Hospital, Helsinki, Finland; 21 Institute for Molecular Medicine Finland, Helsinki, Finland; 22 Hjelt Institute, Department of Public Health, University of Helsinki, Helsinki, Finland; 23 Unit for Sports and Exercise Medicine, Institute of Clinical Medicine, University of Helsinki, Finland; 24 South Ostrobothnia Central Hospital, Seinäjoki, Finland; 25 Red RECAVA Grupo RD06/0014/0015, Hospital Universitario La Paz, Madrid, Spain; 26 Centre for Vascular Prevention, Danube-University Krems, Krems, Austria; 27 Diabetes Endocrine Unit, Clinical Sciences Centre, Aintree University Hospital, University of Liverpool, Liverpool, United Kingdom; 28 Institute of Life Sciences, Swansea University, Swansea, United Kingdom; 29 Institute of Medical Science, University of Toronto, Toronto, Canada; 30 Program in Genetics and Genome Biology, Hospital for Sick Children, Toronto, Canada; 31 National Institute of Diabetes and Digestive and Kidney Diseases (NIDDK), National Institutes of Health, Bethesda, Maryland, United States of America; 32 Biostatics Division, The George Washington University, Washington, D.C., United States of America; 33 Department of Medical Endocrinology, University Hospital of Copenhagen, Copenhagen, Denmark; 34 Faculty of Health Sciences, University of Aarhus, Aarhus, Denmark; 35 Steno Diabetes Center, Gentofte, Denmark; 36 INSERM U872, Paris-Descartes University, Pierre and Marie Curie University, Paris, France; 37 CHU Sart Tilman, Liège, Belgium; 38 CHU Bordeaux, Bordeaux, France; 39 AP-HP, Hôpital Bichat, Diabetology Endocrinology Nutrition, Paris, France; 40 Université Paris Diderot, Sorbonne Paris Cité, UMR 738, Paris, France; 41 INSERM, UMR872, Equipe 2, Centre de Recherche des Cordeliers, Paris, France; 42 INSERM UMR_S 937, ICAN Institute for Cardiometabolism and Nutrition, Pierre and Marie Curie University, Paris, France; 43 Complications of Diabetes Unit, Division of Metabolic and Cardiovascular Sciences, San Raffaele Scientific Institute, Milano, Italy; 44 Department of Molecular Medicine and Surgery, Karolinska Institutet, Stockholm, Sweden; 45 Department of Endocrinology, Metabolism, and Diabetes, Karolinska University Hospital, Stockholm, Sweden; 46 Department of Clinical Sciences, Paediatrics, Umeå University, Umeå, Sweden; 47 Genetics Department, Bucharest University, Bucharest, Romania; 48 University of Medicine and Pharmacy of Craiova, Craiova, Romania; 49 “Carol Davila” University of Medicine and Pharmacy, Bucharest, Romania; 50 Diabetes Epidemiology and Clinical Research Section, NIDDK, Phoenix, Arizona, United States of America; 51 Internal Medicine, Center for Computational Medicine and Bioinformatics, University of Michigan, Ann Arbor, Michigan, United States of America; 52 CHU Poitiers–Endocrinology, University of Poitiers, Poitiers, France; 53 INSERM CIC0802, CHU Poitiers, Poitiers, France; 54 INSERM, U695 (Genetic Determinants of Type 2 Diabetes and Its Vascular Complications), Paris, France; 55 Prosserman Centre for Health Research, Samuel Lunenfeld Research Institute, Toronto, Canada; 56 Division of Biostatistics, Dalla Lana School of Public Health, University of Toronto, Toronto, Canada; 57 Baker IDI Heart and Diabetes Institute, Melbourne, Australia; 58 Regional Nephrology Unit, Belfast City Hospital, Belfast, United Kingdom; University Hospital Regensburg, Germany

## Abstract

Diabetic kidney disease, or diabetic nephropathy (DN), is a major complication of diabetes and the leading cause of end-stage renal disease (ESRD) that requires dialysis treatment or kidney transplantation. In addition to the decrease in the quality of life, DN accounts for a large proportion of the excess mortality associated with type 1 diabetes (T1D). Whereas the degree of glycemia plays a pivotal role in DN, a subset of individuals with poorly controlled T1D do not develop DN. Furthermore, strong familial aggregation supports genetic susceptibility to DN. However, the genes and the molecular mechanisms behind the disease remain poorly understood, and current therapeutic strategies rarely result in reversal of DN. In the GEnetics of Nephropathy: an International Effort (GENIE) consortium, we have undertaken a meta-analysis of genome-wide association studies (GWAS) of T1D DN comprising ∼2.4 million single nucleotide polymorphisms (SNPs) imputed in 6,691 individuals. After additional genotyping of 41 top ranked SNPs representing 24 independent signals in 5,873 individuals, combined meta-analysis revealed association of two SNPs with ESRD: rs7583877 in the *AFF3* gene (*P* = 1.2×10^−8^) and an intergenic SNP on chromosome 15q26 between the genes *RGMA* and *MCTP2*, rs12437854 (*P* = 2.0×10^−9^). Functional data suggest that *AFF3* influences renal tubule fibrosis via the transforming growth factor-beta (TGF-β1) pathway. The strongest association with DN as a primary phenotype was seen for an intronic SNP in the *ERBB4* gene (rs7588550, *P* = 2.1×10^−7^), a gene with type 2 diabetes DN differential expression and in the same intron as a variant with *cis*-eQTL expression of *ERBB4*. All these detected associations represent new signals in the pathogenesis of DN.

## Author Summary

The global prevalence of diabetes has reached epidemic proportions, constituting a major health care problem worldwide. Diabetic kidney disease, or diabetic nephropathy (DN)—the major long term microvascular complication of diabetes—is associated with excess mortality among patients with type 1 diabetes. Even though DN has been shown to cluster in families, the underlying genetic and molecular pathways remain poorly defined. We have undertaken the largest genome-wide association study and meta-analysis to date on DN and on its most severe form of kidney disease, end-stage renal disease (ESRD). We identified new loci significantly associated with diabetic ESRD: *AFF3* and an intergenic locus on chromosome 15q26 residing between *RGMA* and *MCTP2*. Our functional analyses suggest that *AFF3* influences renal tubule fibrosis, a pathological hallmark of severe DN. Another locus in *ERBB4* was suggestively associated with DN and resides in the same intronic region as a variant affecting the expression of *ERBB4*. Subsequent pathway analysis of the genes co-expressed with *ERBB4* indicated involvement of fibrosis.

## Introduction

Diabetic kidney disease, or diabetic nephropathy (DN), is the leading cause of end-stage renal disease (ESRD) worldwide [1]. It affects approximately 30% of patients with long-standing type 1 and type 2 diabetes [2], [3], and confers added risks of cardiovascular disease and mortality. DN is a progressive disorder that is characterized by proteinuria (abnormal loss of protein from the blood compartment into the urine) and gradual loss of kidney function. Early in its course, the kidneys are hypertrophic, and glomerular filtration is increased. However, with progression over several years, proteinuria and decline in kidney function set in, and may result in fibrosis and terminal kidney failure, necessitating costly renal replacement therapies, such as dialysis and renal transplantation. While current treatments that decrease proteinuria will moderately abate DN progression, recent studies show that even with delivery of optimal care, high risks of cardiovascular disease, ESRD and mortality persist [4], [5]. Therefore, discovery of genetic factors that influence development and susceptibility to DN is a critical step towards the identification of novel pathophysiologic mechanisms that may be targeted for interventions to improve the adverse clinical outcomes in diabetic patients.

**Figure 1:**
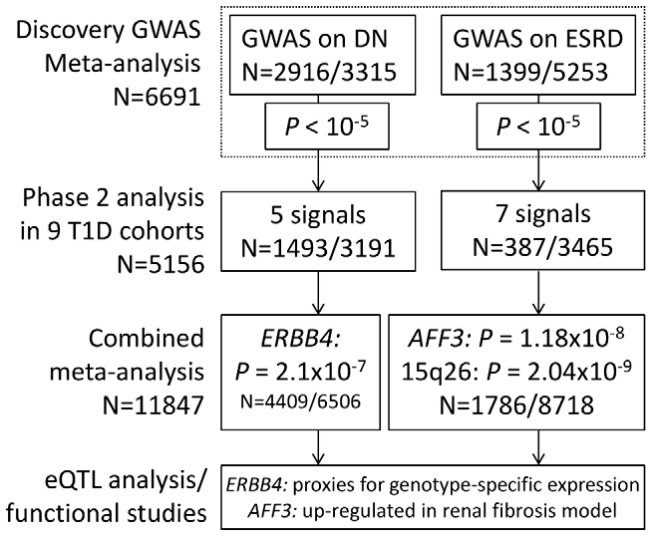
Flow chart summarizing study design. We applied a two stage study design, where the top signals from the meta-analysis of three GENIE studies (UK-ROI, FinnDiane and GoKinD US) were followed up in phase two analysis, consisting of nine T1D cohorts. After combined meta-analysis, two signals reached genome-wide significance in the analysis of ESRD (*P*<5×10^−8^). For DN phenotype no loci reached this threshold, but the strongest association was observed for *ERBB4*. These signals were followed up with eQTL studies and functional analysis. The number of patients (N) refers to the number of samples after genotype quality control; either the total number of samples or divided into cases/controls.

Whereas the degree of glycemia plays a pivotal role in DN, a subset of individuals with poorly controlled type 1 diabetes (T1D) do not develop DN. Furthermore, strong familial aggregation supports genetic susceptibility to DN. The sibling risk of DN has been estimated to be 2.3-fold [6]. While prior studies of individuals with T1D have reported on the possible existence of genetic associations for DN, results have been inconclusive. In GENIE, we leveraged three existing collections for T1D nephropathy (All Ireland Warren 3 Genetics of Kidneys in Diabetes UK Collection [UK-ROI], Finnish Diabetic Nephropathy Study [FinnDiane], and Genetics of Kidneys in Diabetes US Study [GoKinD US]) comprising 6,691 individuals to perform the most comprehensive and well powered DN susceptibility genome-wide association study (GWAS) and meta-analysis to date, with the aim to identify genetic markers associated with DN by meta-analyzing independent GWAS, imputed to HapMap CEU II (Table 1, Figure 1). As a result, we here present two new loci associated with ESRD and a locus suggestively associated with DN.

**Table 1:** Characteristics of samples successfully analyzed in each discovery collection and the meta-analyses.

	UK-ROI		FinnDiane			GoKinD US	
	Cases (n = 823)	Controls (n = 903)	Cases (n = 1,319)	Micro (n = 460)	Controls (n = 1,591)	Cases (n = 774)	Controls (n = 821)
Gender (M/F)	478/345	395/508	785/534	259/201	656/935	402/372	342/479
Duration of T1D (years)	32.9±9.6	27.0±8.6	32.8±9.1	28.2±11.2	27.8±9.5	31.4±7.8	25.4±7.7
Age at diagnosis of T1D (years)	14.5±7.7	14.52±7.8	12.8±7.6	13.2±8.2	15.1±8.3	11±6.6	13±7.3
HbA_1C_ (%)	9.0±1.9	8.7±1.6	8.8±1.6	8.6±1.4	8.1±1.2	7.5±1.9	7.5±1.2
BMI (km/m^2^)	26.3±4.7	26.2±4.2	25.5±4.2	25.9±3.7	25.2±3.5	25.7±5.2	26.1±4.3
ESRD (%)	29.9	0	48.9	0	0	65.6	0

n = total number of patients; Micro = patients with microalbuminuria; M/F = number of males/females; HbA_1C_ blood glycosylated hemoglobin; BMI = body mass index. Case = macroalbuminuria or ESRD, Control = normoalbuminuric, see text for full details.

## Results/Discussion

**Figure 2:**
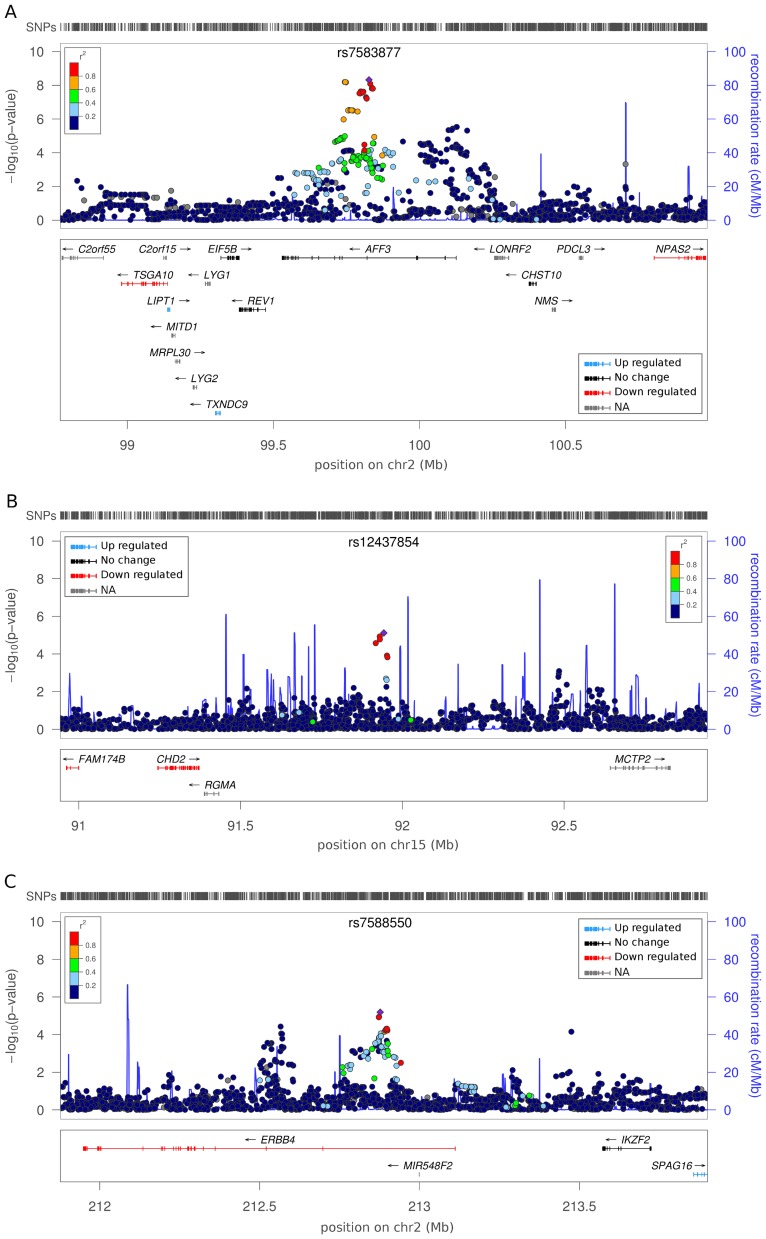
Regional association plots for top ranked SNPs with associated gene expression data. Panels represent independent signals for the primary DN and ESRD analysis. The color of the SNP symbol indicates the linkage disequilibrium (r^2^) with the index SNP which is colored purple. Blue and red gene colors in the lower part of each figure panel indicate up and down regulation in tubulointerstitial or glomerular DN kidney biopsies, respectively. Genes with no change in expression are indicated with black; no data on gene expression with gray color. (A) Association of rs7583877 with ESRD. (B) Association of rs12437854 with ESRD. (C) Association of rs7588550 with DN.

The primary phenotype of interest was DN, defined by the presence of persistent macroalbuminuria or ESRD in individuals aged over 18 who had T1D for at least 10-year duration. Controls were defined as individuals with T1D for at least 15 years but without any clinical evidence of kidney disease (see Methods for more detailed definitions). Meta-analysis of the DN results from each cohort resulted in five independent signals with *P*<10^−5^ (Table S1, Figure S1A). In a parallel analysis of ESRD versus non-ESRD (n cases = 1,399, n controls = 5,253; referred to as “ESRD” analysis throughout the manuscript, unless otherwise stated), SNP rs7583877 on chromosome 2q11.2-q12 achieved genome-wide significance (*P* = 4.8×10^−9^), primarily driven by FinnDiane and the UK-ROI samples, along with six other independent signals reaching *P*<10^−5^ (Figure 2A, Table S1, Figure S1C).

**Figure 3:**
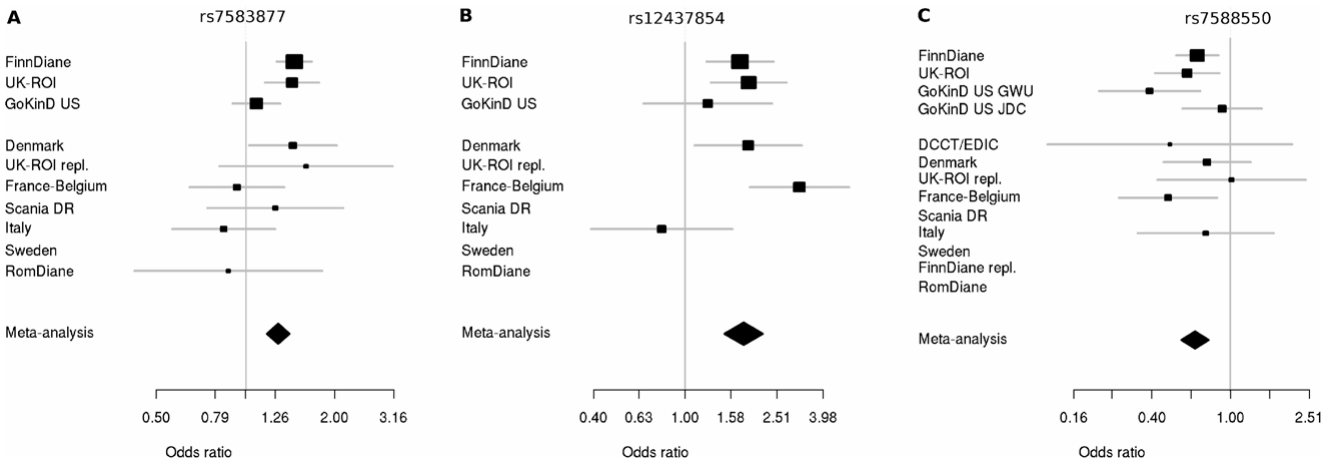
Forest plots for significant hits incorporating discovery and replication plots. Plots show the study-specific association estimates (OR) and 95% confidence intervals for the discovery and second phase studies. (A) Association of rs7583877 with ESRD; heterogeneity *P* = 0.037. (B) Association of rs12437854 with ESRD; heterogeneity *P* = 0.046. (C) Association of rs7588550 with DN; heterogeneity *P* = 0.467. The association estimate and confidence interval for the meta-analysis combining the discovery and second-stage results are denoted by the diamond.

We invited investigators responsible for available collections with similar phenotypes to participate in the secondary genotyping phase of the top ranked SNPs (n = 41 including proxies, representing 24 independent signals) from the initial meta-analysis. Nine independent cohorts contributed 5,873 individuals with comparable phenotypic inclusion criteria (Table S2). After the combined meta-analysis of the first and second phase cohorts, the association of the intronic SNP rs7583877 in *AFF3* with ESRD retained genome-wide significance (odds ratio [OR] = 1.29, 95% confidence interval [CI]: 1.18–1.40, *P* = 1.2×10^−8^; Figure 3A), with the bulk of the association evidence still provided by the FinnDiane and UK-ROI cohorts. The population attributable risk [PAR] for the causal variant underlying the observed association at rs7583877 was estimated to be 3.5%–10.5%. *AFF3* belongs to the AFF (AF4/FMR2) family and encodes a transcriptional activator, with DNA-binding activity, initially found to be fused with *MLL* in some acute lymphoblastic leukemia patients [7], [8]. Recent evidence points to a role for *AFF3* as an RNA-binding protein, with overexpression affecting organization of nuclear speckles and splice machinery integrity [9]. Variants near *AFF3* have been associated with acute lymphoblastic leukemia [10], rheumatoid arthritis [11], [12] and recently T1D [13], [14]. Another locus between the *RGM*A (RGM domain family, member A) and *MCTP2* (multiple C2 domains, transmembrane 2) genes on chromosome 15q26 also reached genome-wide significance for association with ESRD (rs12437854, OR 1.80, 95% CI: 1.48–2.17, *P* = 2.0×10^−9^; Table 2, Figure 3B). PAR estimates for this locus varied from 0.5% to 4.1%. For the primary DN phenotype, an intronic SNP in the *ERBB4* gene demonstrated consistent protective effects in the replication samples and was the top associated SNP identified from the combined discovery and second stage analysis; however, this did not reach genome-wide statistical significance (rs7588550, OR 0.66, 95% CI: 0.56–0.77, *P* = 2.1×10^−7^, PAR 28.3%–32.5% for removal of the major risk allele; Table 2, Figure 3C). *ERBB4* encodes an epidermal growth factor receptor subfamily member, and has been implicated in cardiac, mammary gland and neural development [15], [16]. Mutations in *ERBB4* have previously been reported in cancer [17]. Several studies using Madin-Darby canine kidney (MDCK) cells and conditional *ERBB4* overexpression/knock-out mice, suggest a crucial role for *ERBB4* in renal development and tubulogenesis [18], [19].

**Table 2:** Results from discovery, second stage, and combined meta-analysis for supported markers.

						Discovery		Stage 2		Combined	
SNP	Phenotype	A1	A2	Freq (A1)	Region	OR (95% CI)	*P*-value	OR (95% CI)	*P*-value	OR (95% CI)	*P*-value
rs12437854	ESRD	G	T	0.038	15q26 (*RGMA-MCTP2*)	1.72 (1.36–2.18)	7.6×10^−6^	1.95 (1.41–2.7)	5.4×10^−5^	1.80 (1.48–2.17)	2.0×10^−9^
rs7583877	ESRD	C	T	0.289	2q11.2-q12 (*AFF3*)	1.34 (1.22–1.48)	4.8×10^−9^	1.11 (0.93–1.34)	0.25	1.29 (1.18–1.40)	1.2×10^−8^
rs7588550	T1DN	G	A	0.052	2q33.3-q34 (*ERBB4*)	0.65 (0.55–0.79)	5.3×10^−6^	0.67 (0.49–0.92)	0.01	0.66 (0.56–0.77)	2.1×10^−7^

A1 = minor allele = effect allele; A2 = major allele; Freq(A1) = minor allele frequency; OR = odds ratio; 95% CI = 95% confidence interval. Discovery: Meta analysis results for GENIE discovery cohorts. Stage 2: Meta analysis results for replication cohorts. Combined: Meta analysis results for discovery and the stage 2 cohorts. NA = no result, due to genotype failure or quality control filtering.

It is possible that our observed signal is in linkage disequilibrium with an untyped SNP, or exerts functional effects over an extended genomic region. To explore a putative biological signature we identified, for the top three SNPs, all genes within a 2 Mb window (1 Mb upstream and downstream). Gene ontology analysis revealed no significant enrichment of biological terms or pathways within this subset of flanking genes (Table S3). We determined whether any of these genes were differentially expressed in microarray data derived from tubulointerstitial (n = 49) or glomerular (n = 70) human early DN renal biopsy material versus pre-transplant renal biopsies from living kidney donors (n = 32) [20]. Around rs7583877 (*AFF3*), we noted upregulation of *LIPT1* and *TXNDC9*, while *TSGA10* was downregulated in both tubulointerstitial and glomerular enriched kidney biopsies (Figure 2 and Table S4). *NPAS2*, which flanks rs7583877 (*AFF3*), and *FAM174B* and *CHD2*, which flank rs12437854 (*15q26*), were downregulated in glomerular enriched biopsies of DN patients versus control, but remained unchanged in tubulointerstitial biopsies (Figure 2 and Table S4). *NPAS2* (neuronal PAS domain protein 2), has been implicated in circadian rhythms in the distal nephron segments, acting as a regulator of kidney function [21]. Interestingly, mutations in chromodomain helicase DNA binding protein 2 (*CHD2*), encoding a chromatin-remodeling enzyme, result in impaired glomerular function in mice [22]. Furthermore, at the rs7588550 (*ERBB4*) locus expression of *ERBB4* was down, and *SPAG16* upregulated in tubulointerstitial enriched kidney biopsy tissue of DN versus control subjects (Figure 2 and Table S4).

We also examined whether any of the top three SNPs modulated expression of neighboring genes in *cis* in a dataset of glomerular and tubulointerstitial kidney biopsies of Pima Indians with type 2 diabetes and DN who had been genotyped on the Affymetrix 6.0 array [23]. In Pima Indians, no adequate proxies (haplotype-based D′≥0.8) for the Affymetrix 6.0 SNPs that were strongly correlated with GWAS findings (r^2^≥0.8) could be found for rs12437854, and expression of *AFF3* was below detectable thresholds in this dataset; however, two SNPs in the same intron of *ERBB4* as rs7588550 (rs17418640 and rs17418814) were associated with genotype-specific expression of *ERBB4* in tubulointerstitial but not in glomerular tissue in the Pima cohort (*P*<0.05; Figure S2). Follow-up work is required to investigate the DN associated and eQTL signals in this *ERBB4* intron.

To explore the potential functional role of these *ERBB4* SNPs, we looked for other genes whose expression is correlated with that of *ERBB4*. A total of 388 *ERBB4*-correlated genes were found in the Pima population (Benjamini-Hochberg Q-value<0.1). Pathway analysis of these genes indicates coexpression of *ERBB4* with collagen-related genes, which have been implicated in renal fibrosis [24], [25] (Genomatix Pathway System; Table S5).

**Figure 4:**
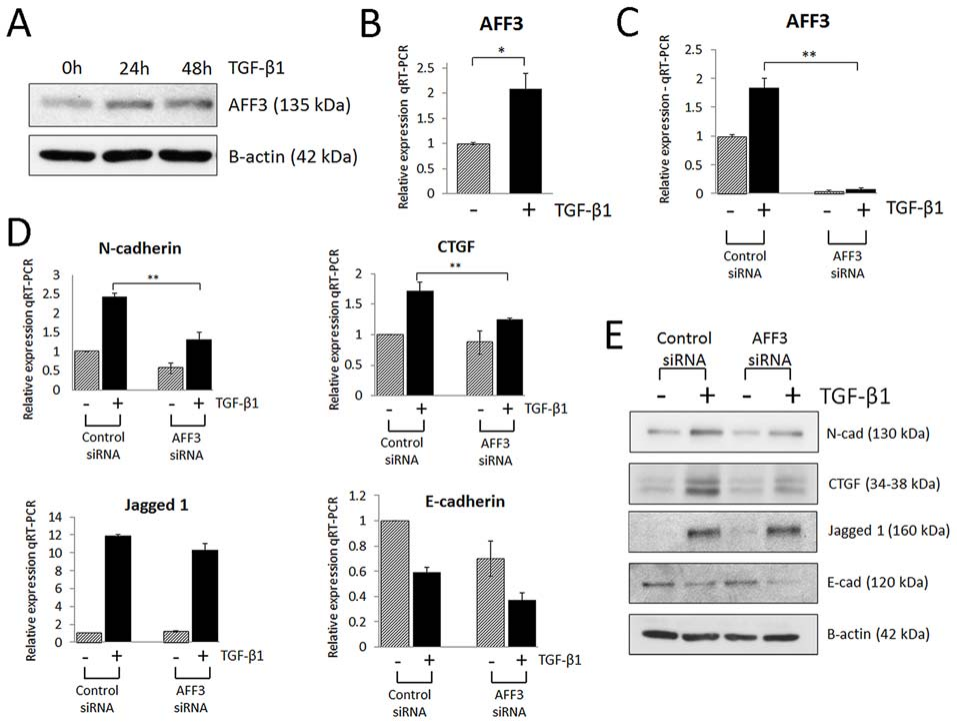
AFF3 is upregulated in renal epithelial cells (HK-2) stimulated with pro-fibrotic TGF-β1. (A) Western blot of AFF3 protein expression in HK-2 cells stimulated with TGF-β1 (5 ng/ml; 24–48 h). (B) TaqMan quantitative PCR analysis of AFF3 mRNA expression in HK-2 cells stimulated with TGF-β1 (5 ng/ml; 48 h) and (C) AFF3 mRNA expression in HK-2 cells transfected with AFF3 siRNA in the presence (black bar)/absence (grey bar) of TGF-β1 (5 ng/ml; 48 h). (D) TaqMan quantitative PCR analysis of N-cadherin, CTGF, Jagged1 and E-cadherin expression in HK-2 cells transfected with AFF3 siRNA in the presence (black bar)/absence (grey bar) of TGF-β1 (5 ng/ml; 48 h). (E) Representative Western blot of N-cadherin, CTGF, Jagged1 and E-cadherin protein responses in HK-2 cells transfected with AFF3 siRNA in the presence/absence of TGF-β1 (5 ng/ml; 48 h). HK-2 cells transfected with control siRNA were selected as a control. For TaqMan PCR, expression was normalized to GAPDH. Data are plotted as mean ± SE (n = 3; *P<0.05, **P<0.01).

Because the low expression level of *AFF3* limited exploration of this gene using expression data, we pursued additional functional experiments in an *in vitro* model of renal fibrosis, namely human tubular epithelia exposed to transforming growth factor-β1 (TGF-β1). Low-level basal expression of the *AFF3* mouse homologue (*LAF4*) has been reported in kidney tubules during embryonic development [26] suggesting proximal renal tubule epithelial cells may be suitable for detection and functional interrogation of *AFF3*. TGF-β1 is implicated in the development of diabetic glomerulosclerosis, and there is recent appreciation of its role as a key driver of tubulointerstitial fibrosis. TGF-β1 induces epithelial cell de-differentiation into a more mesenchymal-like phenotype, characterized by a switch in predominant cadherins from E-cadherin (epithelial) to N-cadherin (mesenchymal), and increased vimentin, α-smooth muscle actin, connective tissue growth factor (CTGF) and Jagged 1 [27], [28]. TGF-β1-mediated loss of E-cadherin in renal epithelia, is believed to be mediated through loss of miR-192 expression [29]. We and others have previously shown that Jagged 1, a ligand for multiple Notch receptors, is up-regulated in human diabetic kidney disease [30], [31], with the Notch signaling pathway implicated in driving renal fibrosis [32], [33]. CTGF is a member of the CCN protein family, with biological roles in differentiation and tissue repair. CTGF is induced by TGF-β1 and enhances expression of multiple extracellular matrix proteins observed in DN, including collagens and fibronectin, and CTGF expression is elevated in the glomeruli of STZ (streptozotocin) - treated rats, an *in vivo* model of T1D [34]. Basal *AFF3* expression was detectable in HK-2 cells, and expression levels were upregulated upon stimulation with TGF-β1 (5 ng/ml; 48 h), as measured at protein and RNA level (Figure 4A–4B). Inhibition of *AFF3* by siRNA attenuated the expression of TGF-β1-driven markers of fibrosis - CTGF and N-cadherin (Figure 4C–4E). Taken together, these data suggest that *AFF*3 may play a role in TGF-β1-induced fibrotic responses of renal epithelial cells.

Traditionally, DN has been viewed as a continuous trait with onset at microalbuminuria, progression to macroalbuminuria, loss of GFR, and culmination in ESRD. Recent studies have called this paradigm into question, suggesting that the syndrome may perhaps be composed of varying phenotypes [35], [36]. Association of rs7583877 (*AFF3*) and rs12437854 (*RGMA – MCTP2*) with the different stages of DN was tested on a time-to-event analysis of relevant endpoints using longitudinal data for participants in the FinnDiane discovery collection. Consistent with our case-control GWAS analyses, the strongest association for rs7583877 was observed for the time from T1D diagnosis to development of ESRD (hazard ratio [HR] 1.33, 95% CI: 1.18–1.49, *P* = 1.9×10^−6^), but also the time from T1D diagnosis to development of macroalbuminuria (HR 1.15, 95% CI: 1.04–1.27, *P* = 0.006) and the time from macroalbuminuria to ESRD (HR 1.16, 95% CI: 1.01–1.36, *P* = 0.04) reached nominal significance. Similarly, rs12437854 was associated with time from T1D diagnosis to development of macroalbuminuria (HR 1.31, 95% CI: 1.03–1.67, *P* = 0.03) and ESRD (HR 1.35, 95% CI: 1.02–1.77, *P* = 0.03) (Text S1, Table S6, Figure S3). When we studied these SNPs and their association with various DN-related phenotypes in the case-control setting of the discovery cohorts, similar observations were made supporting the role of these SNPs in the development of ESRD: Whereas we found evidence of association between rs7583877 (*AFF3*) and all the examined phenotypes with ESRD as the case definition, only moderate association was observed for the DN phenotype (OR = 1.14, *P* = 0.002) and no association when patients with macroalbuminuria were compared to controls with normoalbuminuria (OR = 1.00, *P* = 0.95). rs12437854 (*RGMA* – *MCTP2*) had the strongest association with the original ESRD phenotype (controls defined as all non-ESRD subjects) and with the ESRD vs. normoalbuminuria phenotype, and moderate association with the DN phenotype and comparison of ESRD vs. macroalbuminuric patients (Table S7).

An alternative explanation for our ESRD findings may be that the associated variants in *AFF3* gene and on chromosome 15q26 might be markers of survival. Mortality rates are extremely high in patients with kidney disease and macroalbuminuria, with at least 25% of macroalbuminuric patients dying before they reach ESRD [37]. Thus, the selection of patients with ESRD may be biased towards selection of severe kidney disease survival. To address this question, we used the time until death as the final end point in the longitudinal analysis. Neither of the loci associated with ESRD was also associated with mortality (Text S1, Table S6, Figure S3), suggesting that these loci are associated with ESRD per se.

To explore whether these SNPs contribute to DN via related intermediate phenotypes, such as adiposity, fasting lipid levels, or blood pressure we performed *in silico* searching of publicly available GWAS datasets for our top SNPs [38]–[41]. We found nominal, directionally consistent associations of rs12437854 with fasting glucose (*P* = 0.03) [42] and of rs7583877 with waist-hip ratio (*P* = 0.04) [43] (Table S8). We also considered if previously published T1D and CKD SNP associations were associated with DN or ESRD in our GWAS meta analyses. Eight of 80 SNPs at T1D-associated loci showed nominal significance with DN or ESRD (including three at *AFF3* that are in weak LD [r^2^ 0.030–0.046 in CEU] with the SNPs described here), while no CKD SNPs were nominally significant (Table S9) [44]–[47]. The lack of association with DN for CKD-associated SNPs suggests that the genetic risk factors for DN may differ from the genetic risk factors for CKD in a nondiabetic population.

Finally, to generate further biological hypotheses based on our GWAS results, we employed MAGENTA [48] gene set enrichment analysis software integrating Gene Ontology (GO) terms, KEGG and Ingenuity pathways and PANTHER database entries (Table S10). In the analysis of DN as a case phenotype, enriched gene sets included “sugar binding” (*P* = 0.0006), “double stranded DNA binding” (*P* = 0.001) and “nucleic acid binding” (*P* = 0.004). In the analysis of ESRD significantly enriched gene sets (*P*<0.01) included an enrichment of terms associated with DNA binding, including “sequence-specific DNA binding” (*P* = 0.003), “positive regulation of transcription” (*P* = 0.003), and “homeobox transcription factor” (*P* = 0.004). Taken together, the principal biological signal found within GWAS data suggests an enrichment of transcriptional regulators.

In this largest meta-analysis to date of DN from individuals with T1D, we found two genome-wide significant associations with ESRD. Variants in *AFF3* have been shown to be associated with juvenile idiopathic rheumatoid arthritis, Graves' disease, celiac disease and T1D, indicating this may be a pan-autoimmune disease gene. It is possible that the *AFF3* signal represents an association with T1D and/or is a false positive finding, as it was not seen in the follow-up cohorts. However, we note the following: 1) both FinnDiane and UK-ROI yielded very similar association results, 2) the number of ESRD cases in the replication cohorts is small (n = 363), indicating that statistical power to replicate the original association is limiting, 3) the association result in the second stage, while non-significant, trends in a consistent direction (OR 1.11), 4) after evaluating >12,000 individuals the *AFF3* signal remained genome-wide significant (*P* = 1.2×10^−8^), and 5) we have provided supportive functional evidence that suggests AFF3 may be a relevant contributor to renal disease. Although survival bias is a possibility in the analyses of ESRD, longitudinal analysis revealed the association of the *AFF3* and chromosome 15q26 loci with renal end-points and not with death. Experimental models provide independent evidence of *AFF3* involvement in renal fibrosis and support an association of this locus with a renal phenotype. Importantly, despite our large sample size, we did not achieve genome-wide statistical significance for DN using a combined proteinuria/ESRD phenotype, suggesting that this phenotype may have been too heterogeneous to detect significant associations with a sample of this size. For example, lifelong glycemic control, a known risk factor for DN, is not well captured in most existing cohorts. Nevertheless, this study is the largest, well powered GWAS on DN to date. We demonstrated a suggestive signal of association at *ERBB4* that is supported by experimental data showing haplotype specific mRNA expression in DN biopsies. Our findings reinforce the need for additional studies of patients with T1D and a homogeneous renal phenotype, in whom additional GWAS, fine-mapping and sequencing to uncover rare variants could be performed. Integration of our findings with ongoing GWAS in both type 1 and type 2 diabetes DN may also lead to discovery of additional genetic determinants of DN. The traditional phenotypic definition of DN for individuals with type 2 diabetes may be even more challenging for genetic studies given the heterogeneity of vascular complications and differential renal diagnoses. Several larger-scale GWAS have now been conducted for renal phenotypes [49]–[56], however in most cases the true disease-causing variant and functional impact for specific phenotypes remains to be established. Encouraging reports include the association of uromodulin with CKD [57], *MYH9/APOL1* with non-diabetic ESRD [58], [59], and *PLA2R1* with membranous nephropathy, where anti-PLA2R antibodies appear to predict activity of the disease as well as response to therapy [60].

Our findings point to two transcriptional networks centered around *AFF3* and *ERBB4* that may be operational in the pathogenesis of kidney disease in diabetes.

## Methods

### Ethics statement

All human research was approved by the relevant institutional review boards, and conducted according to the Declaration of Helsinki.

### Study populations

We implemented a two stage analysis, in which a GWAS was performed using a set of three discovery cohorts in the GENIE consortium, and top signals for the DN and ESRD analyses were analyzed further in the second phase in a set of nine independent cohorts (described below) with 5,873 patients in total. The patient numbers in the individual studies are given in Table S11. Additional details are provided in the online material Text S1.

### All Ireland, Warren 3, Genetics of Kidneys in Diabetes UK (UK-ROI) Collection [61]

Inclusion criteria included white individuals with T1D, diagnosed before 31 years of age, whose parents and grandparents were born in the UK and Ireland. The case group comprised 903 individuals with persistent proteinuria (>500 mg/24 h) developing more than 10 years after the diagnosis of diabetes, hypertension (>135/85 mmHg and/or treatment with antihypertensive medication), and retinopathy; ESRD (27.2%) was defined as individuals requiring renal replacement therapy or having received a kidney transplant. Absence of DN was defined as persistent normal urine albumin excretion rate (AER; 2 out of 3 urine albumin to creatinine ratio [ACR] measurements <20 µg of albumin/mg of creatinine) despite duration of T1D for at least 15 years, while not taking an antihypertensive medication, and having no history of treatment with ACE inhibitors; 1,001 individuals formed the control group. After exclusion of patients with low quality DNA samples, 914 DN/ESRD cases and 956 controls remained for the GWAS.

### Finnish Diabetic Nephropathy Study (FinnDiane) [62]

The FinnDiane study is a Finnish cohort of more than 4,800 adult ethnic Finns with T1D, recruited from across Finland, diagnosed prior to age 35 and insulin treatment begun within 1 year. This study comprises 1,721 patients with normal AER, 516 with microalbuminuria, 733 with macroalbuminuria and 682 with ESRD. The disease status was defined by urine AER or urine ACR in at least two out of three consecutive urine collections at local centers: Microalbuminuria was defined as AER≥20<200 µg min^−1^ or ≥30<300 mg/24 h or an ACR of 2.5–25 mg mmol^−1^ for men and 3.5–35 mg mmol^−1^ for women in overnight, 24-hour or spot urine collections, respectively. Similarly, the limit for macroalbuminuria was AER≥200 µg min^−1^ or ≥300 mg/24 h or ACR≥25 mg mmol^−1^ for men and ≥35 mg mmol^−1^ for women. ESRD was defined as ongoing dialysis treatment or transplanted kidney. Control patients with normal AER were required to have T1D duration of at least 15 years. 558 of these patients were included from an independent Finnish cohort collected by the National Institute of Health and Welfare. These patients met the FinnDiane diagnosis and selection criteria, and were analyzed together with the FinnDiane cohort.

### Genetics of Kidneys in Diabetes US Study (GoKinD US) [63]

The GoKinD US study consists of a DN case-control cohort of individuals diagnosed with T1D prior to 31 years of age who began insulin treatment within 1 year of T1D diagnosis. Controls were 18–59 years of age, with T1D for at least 15 years but without DN, n = 889. DN definition includes individuals with ESRD, dialysis or kidney transplant and persistent macroalbuminuria (at least 2 out of 3 tests positive for albuminuria by dipstick ≥1+, or ACR>300 µg albumin/mg of urine creatinine). Cases were defined as people 18–54 years of age, with T1D for at least 10 years and DN, n = 903. Individuals recruited to the control group employed the same inclusion criteria as UK-ROI. Individuals were recruited at two study centers, George Washington University (GWU) and the Joslin Diabetes Centre (JDC) using differing methods of ascertainment and recruitment [64]. Analysis of the GoKinD US cohort was limited to individuals whose primary ethnicity was Caucasian.

### Collections genotyped in Phase 2

DNA was sought from worldwide case-control collections of individuals with T1D and known renal status. A total of 5,873 individuals from nine independent collections were genotyped or imputed for the top-ranked SNPs (n = 41 including 17 proxies), with the exception of the DCCT/EDIC cohort where GWAS data was imputed. All the patients included in the phase two analysis were adults of European descent and had T1D diagnosed before 35 years of age. Controls with normal AER had duration of T1D at least 15 years, and cases with DN had minimum T1D duration of 10 years. If a collection included patients with microalbuminuria, they were excluded from the primary analysis of DN, but included as controls in the analysis of ESRD versus non-ESRD. The main clinical characteristics of all the replication cohorts are shown in the Table S2 and the cohorts are described in Text S1.

### Phenotype definitions

The primary phenotype of interest was DN, defined as individuals aged over 18, with T1D for at least 10 years and diabetic kidney disease. DN includes ESRD or persistent macroalbuminuria as defined in the cohort descriptions above. Controls were defined as individuals with T1D for at least 15 years but without any clinical evidence of kidney disease. Individuals with microalbuminuria were excluded from the primary DN analysis in all cohorts. Disease status definitions were consistent across all the study cohorts. Details of clinical characteristics for each cohort are defined in Table 1 and Table S2. We evaluated a second phenotype to gain further insights into the genetic basis of the most severe form of DN (leading to ESRD), and compared ESRD cases to all those without ESRD. This phenotype is referred to as the “ESRD” or “ESRD vs. non-ESRD” phenotype throughout the manuscript. We also considered individuals with ESRD compared to T1D controls with no clinical evidence of DN. Results for this comparison are given in the online supporting material (Tables S1, S6, S7, S9, S10), where this contrast is called “ESRD vs. normoalbuminuria” or “ESRD vs. normo”.

### Genotyping

DNA from individuals in the UK-ROI collection were genotyped using the Omni1-Quad array (Illumina, San Diego, CA, USA) while FinnDiane samples employed Illumina's BeadArray 610-Quad array. Samples in UK-ROI and FinnDiane were excluded if they had insufficient DNA quality, quantity or poor genotype concordance with previous genotypes during the fingerprint evaluation stage. Existing genotype data for the GoKinD US genotype data was downloaded from dbGAP (phs000018.v2.p1, retrieved June 2010), containing updated genotype data from Affymetrix 500 K set (Affymetrix, Santa Clara, CA, USA).

### Genotype quality control

Samples for UK-ROI and FinnDiane were excluded for insufficient DNA quality, quantity or poor genotype concordance with previous genotypes during a fingerprint evaluation stage. In the UK-ROI sample, 1,830 unique case (n = 872) and control (n = 958) individuals were submitted for genotyping on the Omni1-Quad. For FinnDiane, 3,651 individuals (cases, n = 1,934; controls n = 1,721) were submitted for genotyping on the 610-Quad. For all three discovery datasets (UK-ROI, FinnDiane, GoKinD US), uniform and extensive genotype quality control procedures were applied: SNPs were filtered for those with call rates greater than 90%, minor allele frequency (MAF) exceeding 1%, and concordance with Hardy Weinberg Equilibrium (HWE, *P*<10^−7^). Sample filters included individual call rates greater than 95%, no extreme heterozygosity and cryptic relatedness as determined using identity by descent (first degree relatives, estimated identity by descent >0.4), and admixture assessment using principal components (plotted with HapMap reference panel, Figure S4). Additional quality control measures included test of missing by haplotype (*P*<10^−8^), missing by phenotype (*P*>10^−8^) and plate effects (*P*<10^−7^). These quality control steps were performed using PLINK [65] with custom Perl and R analysis scripts. Known copy number variation and mitochondrial SNPs were excluded from analyses. Detailed results of each QC step are reported in Table S12 for each study population.

A HapMap control sample was included on all genotyping plates for UK-ROI; average call rate was 99.9% with HapMap concordance equaling 99.7%. The average sample call rate was 99.5% in UK-ROI with sample heterozygosity 22.1%. Concordance with internal control for FinnDiane was 99.996% with an average sample call rate of 99.8%.

Principal Component Analysis (PCA) was performed separately for each of the three studies with the EIGENSTRAT program [66] in order to detect genetic outliers and to adjust the analyses for population structure. Genetic outliers were defined as more than six standard deviations away from the center of distribution along any of the ten first principal components and the procedure was repeated until no outliers were detected. After filtering, PCA were calculated for each study cohort combined with unrelated individuals from three original HapMap populations (www.hapmap.org), and plotted to identify additional admixed individuals. The first ten principal components were employed to adjust the association analysis for any residual population structure from the cleaned datasets.

In total, directly genotyped results for 823 cases and 903 controls in 791,687 SNPs passed QC procedure in UK-ROI. Similarly, 549,530 SNPs with average genotyping rate of 99.9% passed the QC filters in 1,319 cases, 1,591 controls and 460 individuals with microalbuminuria for FinnDiane. 360,899 SNPs in 774 cases and 821 controls for GoKinD US passed quality control and were included in the analysis.

### Imputation

Imputation was performed after the quality control employing MACH 1.0 software (http://www.sph.umich.edu/csg/abecasis/MACH) with HapMap phase II CEU population as a reference, resulting in ∼2.4 million SNPs for each cohort. The cross-over and error rates were estimated with 50 iteration rounds in roughly 300 randomly selected samples. The imputation was run with the greedy algorithm and the maximum likelihood method in order to obtain expected allele dosages rather than integer allele counts. SNPs with low imputation quality (r^2^<0.6) are not reported.

### Statistical analysis

PLINK v1.07 [67] was employed to conduct association tests for the allele dosage data with logistic regression adjusted for sex, age, the duration of diabetes and the ten first components of the study specific principal component analysis. UK-ROI and GoKinD US were adjusted for study center, but in the primary DN phenotype the two GoKinD US centers; GWU and JDC, were analyzed separately. Results from individual studies were adjusted for study specific genomic inflation factor and then combined by fixed effect meta-analysis model using METAL [68], to estimate the combined effect sizes and significances from beta values and standard error. Regional association plots were generated using hg18 in LocusZoom [69]. Quantile-Quantile plots were generated to evaluate the number and magnitude of observed associations compared with those expected under the null hypothesis (Figure S1).

### Second-phase SNP selection and genotyping

All SNPs observed with *P*<10^−5^ were selected for further analysis. These SNPs were reviewed and a top SNP (with a proxy) was selected for each independent signal (SNPs more than 500 kb distant or LD r^2^<0.3 in HapMap II CEU) using the LD-based clumping procedure implemented in PLINK. *De novo* genotyping was performed for all phase two cohorts except for DCCT/EDIC using identical designs of Sequenom IPLEX assays (Sequenom Inc, San Diego, US). The DCCT/EDIC samples were imputed from their GWAS results that had undergone their respective quality control procedure. The statistical analysis was similar to the discovery cohorts with the difference that the models were not adjusted for principal components. All results were then combined by meta-analysis using METAL software as previously described.

### Longitudinal analysis

Time to event analyses were performed on longitudinal data from the FinnDiane discovery cohort using Kaplan-Meier and Cox proportional hazards regression with the aim to evaluate the genetic association of rs7583877 and rs12437854 with time from the diagnosis of T1D to the onset of the following end points: microalbuminuria, macroalbuminuria or ESRD. Additionally, we analyzed time from onset of macroalbuminuria to development of ESRD. The most recent kidney status data were utilized for each patient. We also examined if the two main association loci, rs7583877 and rs12437854, were associated with mortality using data from the Finnish Death Registry (as per 30.9.2010). As DN (defined as macroalbuminuria or ESRD) is strongly associated with mortality, the time to death was separately analyzed for patients without DN (time from T1D onset to death; patients who developed DN were censored at the time of the onset of DN) and for those with DN (time from onset of DN to death and time from onset of ESRD to death). Analyses were performed using the ‘survival’ package in R software (version 2.36-10, http://cran.r-project.org/web/packages/survival). (See Text S1.)

### Additional analyses

SNPs were annotated with associated genes and function using dbSNP build 132, human build 37.1. Cytogenetic locations for genes were sourced from Entrez gene; locations for SNPs that were not associated with genes were recorded from NCBI MapView. *In silico* analyses included gene set enrichment using MAGENTA [48]. To explore functional implications of *AFF3*, human kidney epithelial cells (HK-2) were cultured and evaluated (Figure 4).

### Renal biopsy populations

Gene expression was measured in renal tissue compartments micro-dissected from renal biopsies from Pima Indians with type 2 diabetes and early stage DN (n = 77), as well as from Caucasian living kidney transplant donors (n = 20). Pima Indian subjects are 25–68 in age, with measured ACR in the range 5.23–7162, and GFR in the range 40.45–274.80.

### Renal expression

Renal biopsies were micro-dissected into glomeruli and either tubulointerstitial or cortical compartments, and gene expression measured using the Affymetrix HGU-133A and HGU-133 Plus 2 platforms [70]. Background adjustment, quantile normalization and probe-set summarization were performed with in a GenePattern (www.genepattern.org) pipeline using Robust Multichip Analysis [71] with batch correction using Combat [72]. The differential expression data sets were processed with the Entrez Gene Custom CDF v.10, and the eQTL data sets were processed with the RefSeq Custom CDF v.12 [73] for probe-sets common to both expression platforms.

### eQTL association

The Affymetrix 6.0 genotyping platform was used to genotype Pima Indians with glomerular expression (n = 65), a subset of which (n = 54) also had tubulointerstitial/cortical expression. The *cis* region of each gene was defined as 150 kb upstream of the transcript start site and 50 kb downstream of the transcription end site.

## Supporting Information

Figure S1 Manhattan and QQ-plots for DN and ESRD phenotypes. Manhattan plots (panels A and C) highlighting *P* values from the discovery meta-analysis where dotted horizontal lines represent the threshold for follow up, *P*<1×10^−5^, and the solid horizontal lines indicate the threshold for genome-wide significance, *P*<5×10^−8^. The nearest genes are indicated above regions of interest. SNPs that reached threshold *P*<1×10^−5^ and were selected for follow up are denoted as black diamonds, SNPs in linkage disequilibrium (R>0.6) with top SNP are denoted with blue dots, and final meta analysis *P* values (discovery+phase 2 results) as red triangles. Q-Q plots (panels B and D) evaluated inflation of the GWAS results and show the expected versus observed *P* values; the diagonal line is the line of identity. The inflation factor λ for the genomic control is indicated in the Q-Q plots.
(TIF)

Figure S2 Box and whisker plots of normalized *ERBB4* expression intensities in glomerulus (A,B) and tubulointerstitium (C,D) by genotype showing eQTL associations in tubulointerstitium. Both SNPs show significant eQTL associations in tubulointerstitial kidney biopsies of Pima Indians with type 2 diabetes and DN (*P* = 0.018 for rs1718640, *P* = 0.024 for rs17418814; linear regression using additive model). Association remained significant for rs17418640 when the subject with homozygous minor allele was excluded (*P* = 0.043). Associations with glomerular expression are not significant. Gene expression in kidneys was evaluated with Affy HGU-133A custom CDF probesets annotated to RefSeq transcripts NM_005235 and NM_001042599, and SNPs were genotyped with Affy 6.0 genotyping platform. Conditional analysis indicates rs17418814 is dependent on rs1718640 (*P* = 0.95 conditioned on rs1718640, versus rs1718640 *P* = 0.48 conditioned on rs17418814). Both SNPs lie within the same intron of the *ERBB4* gene as rs7588550 that was suggestively associated with DN.
(TIF)

Figure S3 Longitudinal analyses in FinnDiane for rs7583877 (*AFF3*) and rs12437854 (chromosome 15q26). Analyses assume an additive model of the SNP effects. The plotted survival curves have been truncated at the point at which fewer than five participants remained with the corresponding genotype. The genotype legend in each figure indicates the number of samples with the corresponding genotype, shown in parentheses. The *P*-value is indicated for the nominally significant associations (*P*<0.05). ns = not significant. The bottom part of each figure indicates the number of samples at risk at ten-year intervals.
(TIF)

Figure S4 Rooted Principal Component Analysis of the discovery cohorts. Two first principal components (PC1 and PC2) are shown for (A) UK-ROI, (B) FinnDiane and (C) GoKinD US. Principal Component Analysis was calculated with EIGENSTRAT software including CEU, YRI and CBT from HapMap II as reference samples.
(TIF)

Table S1 Top ranked SNPs selected for DN, ESRD vs. non ESRD, and ESRD vs. normoalbuminuria phenotypes.
(DOC)

Table S2 Clinical characteristics and information on genotyping of the phase two cohorts.
(XLS)

Table S3 Gene ontology analysis of all genes within ±1 Mbp of top GWAS signals: rs7583877/*AFF3*; rs12437854/15q26; rs7588550/*ERBB4*.
(DOC)

Table S4 Gene expression in early DN versus living donor kidney biopsies. All genes within a 2 Mb window (1 Mb upstream and downstream) of the three main signals (rs7583877/*AFF3*, rs12437854/15q26, rs7588550/*ERBB4*) were studied.
(DOC)

Table S5 Significantly enriched pathways (Genomatix Pathway System) for the *ERBB4*-correlated genes in early diabetic nephropathy.
(DOC)

Table S6 Cross-sectional and longitudinal analyses in FinnDiane for rs7583877 (*AFF3*) and rs12437854 (chromosome 15q26).
(DOC)

Table S7 Additional kidney phenotype analysis results for the three main loci.
(DOC)

Table S8 *P*-value for association with DN related traits for the main signals after combined meta-analysis of DN and ESRD phenotypes. A1 is associated with increasing risk of ESRD/DN.
(DOC)

Table S9 GENIE GWAS associations for SNPs that have been previously associated with T1D or chronic kidney disease.
(DOC)

Table S10 Gene set enrichment analysis with MAGENTA. Gene sets with nominal *P*-value<0.01 for the three analyzed phenotypes.
(DOC)

Table S11 Number of patients included in the study.
(DOC)

Table S12 Quality control and filtering for the discovery GWAS data.
(DOC)

Table S13 Physicians and nurses participating in the collection of the FinnDiane study subjects.
(DOC)

Table S14 The DCCT/EDIC Study Research Group.
(DOC)

Text S1 Supplementary Methods: Detailed explanation of employed methods.
(DOC)
