# Supplementary figures and images for: New Susceptibility Loci Associated with Kidney Disease in Type 1 Diabetes
Source: PLoS Genet. 2012 Sep 20;8(9):e1002921. doi: 10.1371/journal.pgen.1002921 (PMC3447939; doi:10.1371/journal.pgen.1002921)

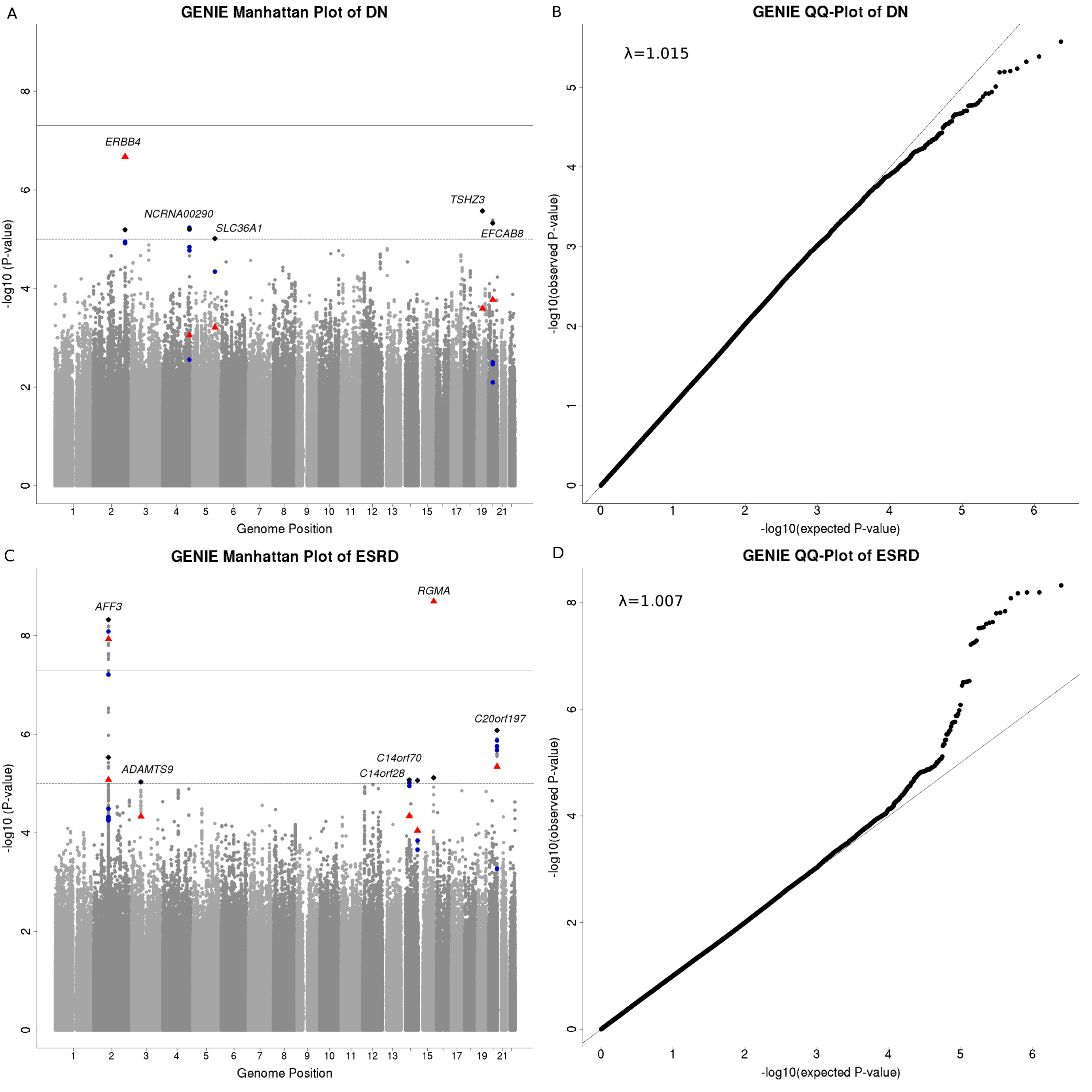

Supplement: Figure S1 — Manhattan and QQ-plots for DN and ESRD phenotypes. Manhattan plots (panels A and C) highlighting P values from the discovery meta-analysis where dotted horizontal lines represent the threshold for follow up, P<1×10^−5^, and the solid horizontal lines indicate the threshold for genome-wide significance, P<5×10^−8^. The nearest genes are indicated above regions of interest. SNPs that reached threshold P<1×10^−5^ and were selected for follow up are denoted as black diamonds, SNPs in linkage disequilibrium (R>0.6) with top SNP are denoted with blue dots, and final meta analysis P values (discovery+phase 2 results) as red triangles. Q-Q plots (panels B and D) evaluated inflation of the GWAS results and show the expected versus observed P values; the diagonal line is the line of identity. The inflation factor λ for the genomic control is indicated in the Q-Q plots. (TIF) [file pgen.1002921.s001.tif]

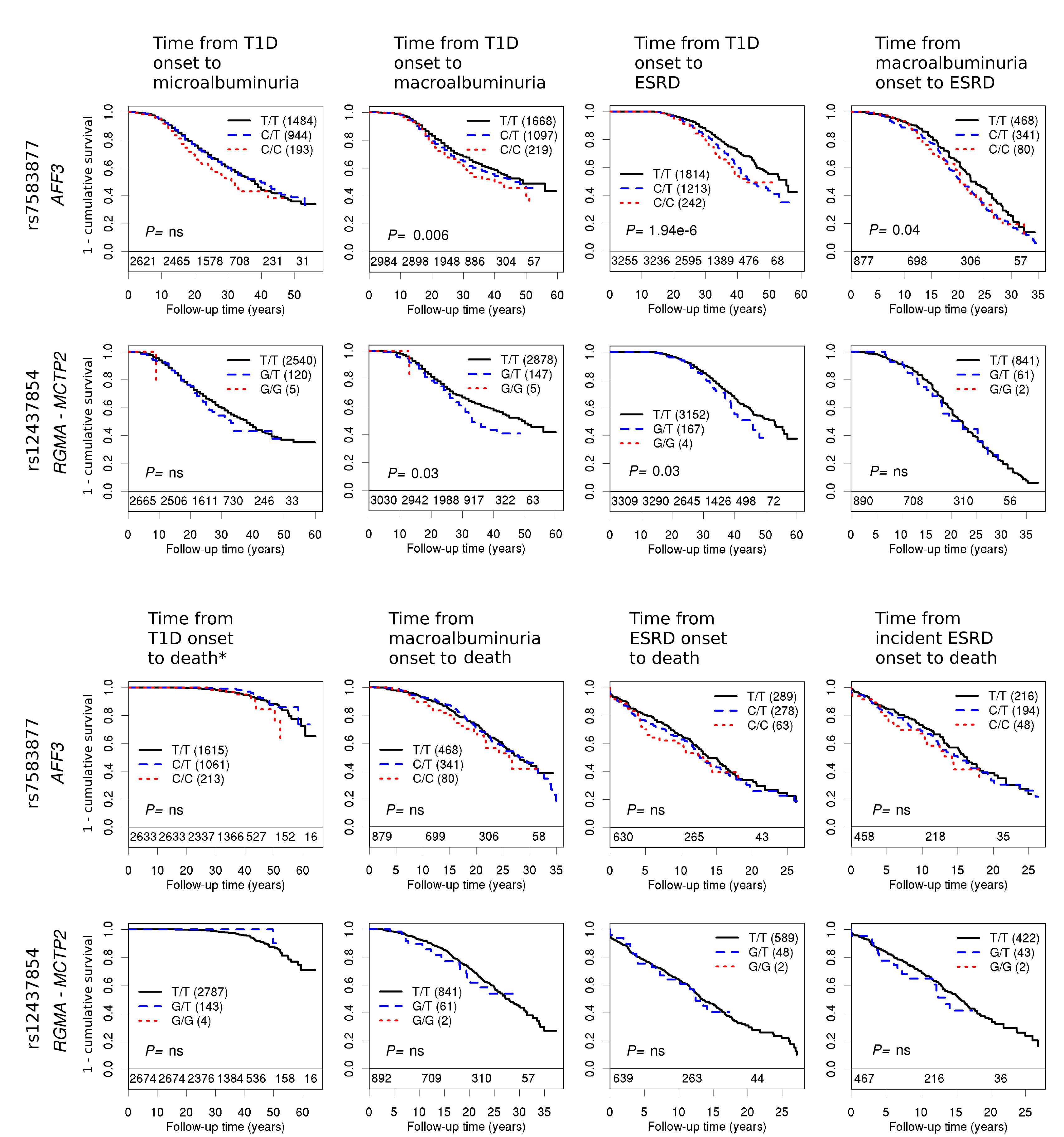

Supplement: Figure S3 — Longitudinal analyses in FinnDiane for rs7583877 (AFF3) and rs12437854 (chromosome 15q26). Analyses assume an additive model of the SNP effects. The plotted survival curves have been truncated at the point at which fewer than five participants remained with the corresponding genotype. The genotype legend in each figure indicates the number of samples with the corresponding genotype, shown in parentheses. The P-value is indicated for the nominally significant associations (P<0.05). ns = not significant. The bottom part of each figure indicates the number of samples at risk at ten-year intervals. (TIF) [file pgen.1002921.s003.tif]

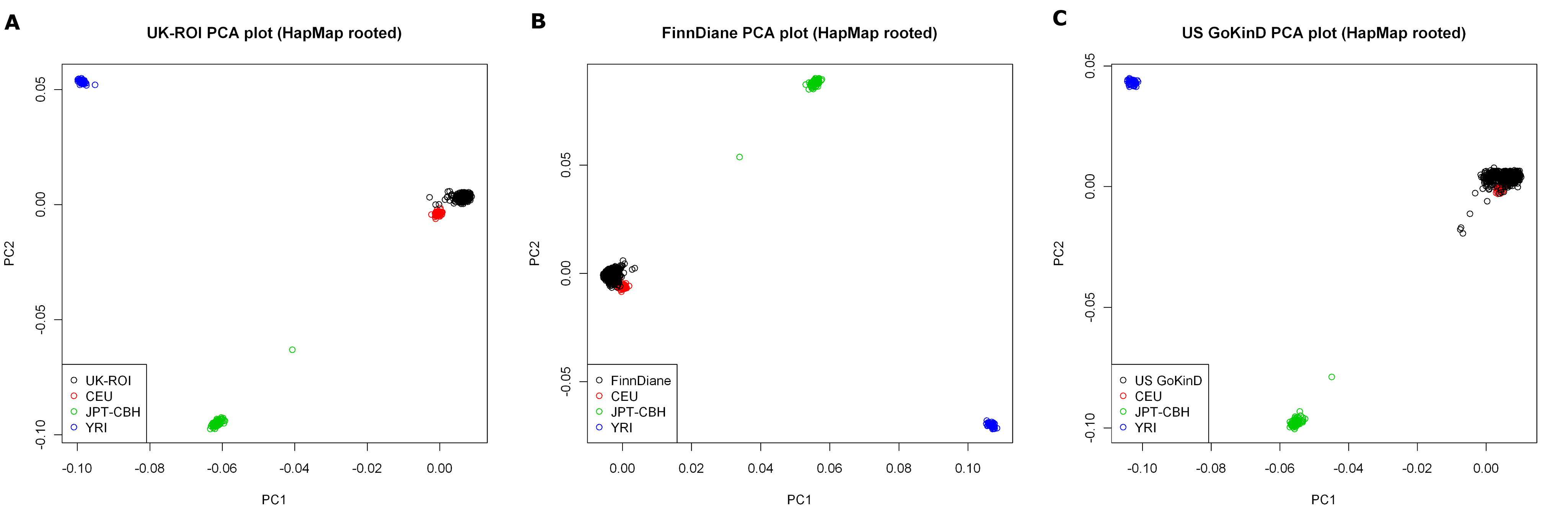

Supplement: Figure S4 — Rooted Principal Component Analysis of the discovery cohorts. Two first principal components (PC1 and PC2) are shown for (A) UK-ROI, (B) FinnDiane and (C) GoKinD US. Principal Component Analysis was calculated with EIGENSTRAT software including CEU, YRI and CBT from HapMap II as reference samples. (TIF) [file pgen.1002921.s004.tif]
