# Supplementary material for: New Susceptibility Loci Associated with Kidney Disease in Type 1 Diabetes
Source: PLoS Genet. 2012 Sep 20;8(9):e1002921. doi: 10.1371/journal.pgen.1002921 (PMC3447939; doi:10.1371/journal.pgen.1002921)
Supplement: Table S1 — Top ranked SNPs selected for DN, ESRD vs. non ESRD, and ESRD vs. normoalbuminuria phenotypes. (DOC) [file pgen.1002921.s005.doc]

**Table S1. Top ranked SNPs selected for DN, ESRD vs. non ESRD, and ESRD vs. normoalbuminuria phenotypes.**

|  |  |  |  |  |  | Discovery | |  | Stage 2 | |  | Combined | | |
| --- | --- | --- | --- | --- | --- | --- | --- | --- | --- | --- | --- | --- | --- | --- |
| SNP | Proxy | A1 | A2 | Freq | Region | OR (95% CI) | P-value |  | OR (95% CI) | P-value |  | OR (95% CI) | P-value | Het P |
| **DN** |  |  |  |  |  |  |  |  |  |  |  |  |  |  |
| rs7588550 | | G | A | 0.05 | 2q34 (*ERBB4*) | 0.65 (0.55 - 0.79) | 5.31E-06 |  | 0.67 (0.49 - 0.92) | 0.013 |  | 0.66 (0.56 - 0.77) | 2.14E-07 | 0.467 |
|  | rs10170838 | C | T | 0.05 | 2q34 (*ERBB4*) | 0.66 (0.55 - 0.8) | 9.67E-06 |  | 0.71 (0.52 - 0.97) | 0.033 |  | 0.67 (0.58 - 0.79) | 9.76E-07 | 0.349 |
| rs13045180 | | T | C | 0.19 | 20q11.21 | 1.26 (1.14 - 1.39) | 4.39E-06 |  | 1.01 (0.89 - 1.15) | 0.832 |  | 1.16 (1.07 - 1.26) | 1.70E-04 | 0.148 |
| rs12973926 | | T | A | 0.10 | 19q12 | 0.70 (0.60 - 0.81) | 3.25E-06 |  | 0.98 (0.83 - 1.16) | 0.804 |  | 0.81 (0.73 - 0.91) | 2.56E-04 | 0.026 |
| rs17802828 | | T | C | 0.17 | 5q33.1 (*SLC36A1*) | 1.27 (1.14 - 1.41) | 9.90E-06 |  | 0.99 (0.86 - 1.13) | 0.839 |  | 1.16 (1.07 - 1.26) | 6.10E-04 | 0.195 |
|  | rs11954054 | G | A | 0.22 | 5q33.1 (*SLC36A1*) | 1.22 (1.11 - 1.34) | 4.82E-05 |  | 0.99 (0.87 - 1.13) | 0.909 |  | 1.14 (1.05 - 1.23) | 1.15E-03 | 0.235 |
| rs9683583 | | A | G | 0.12 | 4q34.3 | 1.32 (1.17 - 1.49) | 6.49E-06 |  | 0.87 (0.70 - 1.08) | 0.211 |  | 1.19 (1.08 - 1.33) | 8.91E-04 | 0.071 |
| **ESRD vs. non-ESRD** | |  |  |  |  |  |  |  |  |  |  |  |  |  |
| rs12437854 | | G | T | 0.04 | 15q26.2 | 1.72 (1.36 - 2.18) | 7.65E-06 |  | 1.95 (1.41 - 2.70) | 5.40E-05 |  | 1.8 (1.48 - 2.17) | 2.04E-09 | 0.046 |
|  | rs17709163 | G | A | 0.03 | 15q26.1 (*RP11-266O8.1*) | 1.73 (1.34 - 2.23) | 2.68E-05 |  | 1.90 (1.29 - 2.80) | 1.15E-03 |  | 1.78 (1.44 - 2.2) | 1.19E-07 | 0.653 |
| rs7583877 | | C | T | 0.29 | 2q11.2 (*AFF3*) | 1.34 (1.22 - 1.48) | 4.76E-09 |  | 1.11 (0.93 - 1.34) | 0.246 |  | 1.29 (1.18 - 1.4) | 1.18E-08 | 0.037 |
| rs6027506 | | G | A | 0.37 | 20q13.33 (*RP5-1043L13.1*) | 0.79 (0.71 - 0.87) | 8.31E-07 |  | 0.95 (0.80 - 1.13) | 0.574 |  | 0.82 (0.76 - 0.89) | 4.54E-06 | 0.059 |
|  | rs11698685 | A | G | 0.32 | 20q13.33 (*RP5-1043L13.1*) | 0.78 (0.70 - 0.86) | 1.33E-06 |  | 0.95 (0.80 - 1.15) | 0.619 |  | 0.82 (0.75 - 0.89) | 7.85E-06 | 0.059 |
| rs12712065 | | C | G | 0.50 | 2q11.2 (*AFF3*) | 1.24 (1.13 - 1.36) | 2.96E-06 |  | 1.07 (0.91 - 1.25) | 0.430 |  | 1.20 (1.11 - 1.30) | 8.43E-06 | 0.615 |
|  | rs6732141 | G | C | 0.50 | 2q11.2 (*AFF3*) | 1.24 (1.13 - 1.35) | 4.51E-06 |  | 1.05 (0.90 - 1.24) | 0.520 |  | 1.19 (1.10 - 1.29) | 1.68E-05 | 0.449 |
| rs10483552 | | C | G | 0.02 | 14q21.2 (*RP11-398E10.1*) | 1.92 (1.44 - 2.55) | 8.45E-06 |  | 0.78 (0.32 - 1.91) | 0.583 |  | 1.76 (1.34 - 2.32) | 4.56E-05 | 0.095 |
|  | rs10483551 | G | A | 0.02 | 14q21.2 | 1.90 (1.43 - 2.52) | 9.73E-06 |  | NA | NA |  | 1.90 (1.43 - 2.52) | 9.73E-06 | 0.209 |
| rs11719363 | | T | G | 0.32 | 3p14.1 (*ADAMTS9*) | 1.26 (1.14 - 1.40) | 9.28E-06 |  | 1.01 (0.81 - 1.26) | 0.929 |  | 1.21 (1.11 - 1.33) | 4.66E-05 | 0.354 |
|  | rs7652817 | A | G | 0.30 | 3p14.1 (*ADAMTS9*) | 1.24 (1.12 - 1.37) | 2.28E-05 |  | 1.06 (0.88 - 1.29) | 0.526 |  | 1.20 (1.10 - 1.31) | 5.02E-05 | 0.498 |
| rs1018534 | | T | G | 0.37 | 14q32.2 (*RP11-566J3.1*) | 0.80 (0.72 - 0.88) | 8.63E-06 |  | 0.99 (0.83 - 1.19) | 0.948 |  | 0.84 (0.77 - 0.92) | 9.06E-05 | 0.350 |
|  | rs1467537 | T | C | 0.34 | 14q32.2 (*RP11-566J3.1*) | 0.81 (0.73 - 0.91) | 2.19E-04 |  | 0.92 (0.64 - 1.31) | 0.641 |  | 0.82 (0.74 - 0.91) | 2.42E-04 | 0.426 |
| A1 = Minor allele = effect allele, A2 = Major Allele, MAF = minor allele frequency, Region = Chromosomal region and associated gene, OR = odds ratio for minor allele, 95% CI = 95% confidence interval, Het P = P-value for meta-analysis heterogeneity test, Discovery: Meta analysis results for 3 GENIE discovery cohorts. Stage 2: Meta analysis results for 9 replication cohorts. Combined: Meta analysis results for discovery and the 10 stage 2 cohorts. NA= no result, due to genotype failure or quality control filtering. | | | | | | | | | | | | | | |
|
|
|

**Table S1. Continued**

|  |  |  |  |  |  | Discovery | |  | Stage 2 | |  | Combined | | |
| --- | --- | --- | --- | --- | --- | --- | --- | --- | --- | --- | --- | --- | --- | --- |
| SNP | Proxy | A1 | A2 | Freq | Region | OR (95% CI) | P-value |  | OR (95% CI) | P-value |  | OR (95% CI) | P-value | Het P |
| **ESRD vs normoalbuminuria** | | | | |  |  |  |  |  |  |  |  |  |  |
| rs7583877 | | C | T | 0.30 | 2q11.2 (*AFF3*) | 1.33 (1.19 - 1.48) | 4.87E-07 |  | 1.16 (0.95 - 1.41) | 0.139 |  | 1.29 (1.17 - 1.42) | 3.27E-07 | 0.119 |
| rs11723864 | | G | C | 0.11 | 4q34.1 | 1.50 (1.26 - 1.80) | 6.81E-06 |  | 1.57 (1.03 - 2.38) | 0.035 |  | 1.51 (1.29 - 1.78) | 6.89E-07 | 0.019 |
| rs4901737 | | G | T | 0.12 | 14q23.1 | 1.55 (1.30 - 1.85) | 1.47E-06 |  | 1.23 (0.84 - 1.79) | 0.288 |  | 1.49 (1.26 - 1.75) | 1.51E-06 | 0.633 |
|  | rs878889 | A | G | 0.13 | 14q22.3 (*RP11-1085N6.4*) | 1.43 (1.23 - 1.65) | 1.81E-06 |  | 1.54 (0.77 - 3.05) | 0.219 |  | 1.43 (1.24 - 1.65) | 8.43E-07 | 0.920 |
| rs11698685 | | A | G | 0.32 | 20q13.33 (*RP5-1043L13.1*) | 0.75 (0.67 - 0.84) | 2.80E-07 |  | 0.93 (0.76 - 1.13) | 0.453 |  | 0.79 (0.72 - 0.87) | 1.34E-06 | 0.037 |
|  | rs6027506 | G | A | 0.37 | 20q13.33 (*RP5-1043L13.1*) | 0.77 (0.69 - 0.85) | 8.21E-07 |  | 0.92 (0.77 - 1.11) | 0.396 |  | 0.80 (0.73 - 0.88) | 2.65E-06 | 0.048 |
| rs4871297 | | G | A | 0.48 | 8q24.13 (*RP11-973F15.1*) | 0.79 (0.71 - 0.87) | 3.41E-06 |  | 0.87 (0.73 - 1.04) | 0.119 |  | 0.81 (0.74 - 0.88) | 1.64E-06 | 0.764 |
|  | rs10094408 | T | C | 0.47 | 8q24.13 (*RP11-973F15.1*) | 0.79 (0.71 - 0.87) | 4.18E-06 |  | 0.88 (0.74 - 1.05) | 0.145 |  | 0.81 (0.74 - 0.88) | 2.50E-06 | 0.775 |
| rs1167726 | | C | A | 0.17 | 12q24.31 (*RNF10 - COQ5*) | 0.71 (0.62 - 0.82) | 2.27E-06 |  | NA | NA |  | 0.71 (0.62 - 0.82) | 2.27E-06 | 0.435 |
|  | rs614226 | T | C | 0.17 | 12q24.31 (*RNF10 - COQ5*) | 0.71 (0.62 - 0.82) | 2.37E-06 |  | 0.85 (0.66 - 1.10) | 0.217 |  | 0.74 (0.65 - 0.84) | 2.25E-06 | 0.633 |
|  | rs7174 | A | G | 0.17 | 12q24.31 (*RNF10 - POP5*) | 0.71 (0.62 - 0.82) | 2.44E-06 |  | 0.87 (0.68 - 1.12) | 0.290 |  | 0.75 (0.66 - 0.84) | 3.62E-06 | 0.551 |
| rs17013700 | | A | G | 0.01 | 3p24.3 | 3.21 (1.93 - 5.31) | 6.10E-06 |  | NA | NA |  | 3.21 (1.93 - 5.31) | 6.10E-06 | 0.269 |
| rs13045180 | | T | C | 0.19 | 20q11.21 | 1.35 (1.19 - 1.53) | 4.18E-06 |  | 1.11 (0.90 - 1.37) | 0.334 |  | 1.28 (1.15 - 1.43) | 9.10E-06 | 0.378 |
| rs11719363 | | T | G | 0.31 | 3p14.1 (*ADAMTS9*) | 1.30 (1.16 - 1.46) | 5.35E-06 |  | 1.06 (0.83 - 1.34) | 0.640 |  | 1.25 (1.13 - 1.39) | 1.63E-05 | 0.574 |
|  | rs7652817 | A | G | 0.30 | 3p14.1 (*ADAMTS9*) | 1.28 (1.15 - 1.43) | 8.86E-06 |  | 1.08 (0.88 - 1.32) | 0.456 |  | 1.23 (1.12 - 1.36) | 2.03E-05 | 0.698 |
| rs12712065 | | C | G | 0.50 | 2q11.2 (*AFF3*) | 1.25 (1.13 - 1.39) | 9.41E-06 |  | 1.07 (0.90 - 1.27) | 0.435 |  | 1.20 (1.10 - 1.31) | 2.50E-05 | 0.339 |
|  | rs6732141 | G | C | 0.50 | 2q11.2 (*AFF3*) | 1.26 (1.14 - 1.39) | 8.64E-06 |  | 1.06 (0.90 - 1.26) | 0.470 |  | 1.20 (1.10 - 1.31) | 2.69E-05 | 0.233 |
| rs4237928 | | T | C | 0.23 | 12p13.2 (*RP11-291B21.2*) | 1.31 (1.16 - 1.47) | 7.75E-06 |  | 1.02 (0.82 - 1.25) | 0.887 |  | 1.23 (1.11 - 1.36) | 7.27E-05 | 0.289 |
|  | rs4763548 | C | T | 0.23 | 12p13.2 (*RP11-291B21.2*) | 1.31 (1.16 - 1.47) | 7.75E-06 |  | 1.00 (0.65 - 1.53) | 0.985 |  | 1.28 (1.14 - 1.43) | 1.65E-05 | 0.092 |
| rs13282135 | | T | A | 0.49 | 8q24.3 (*ZNF696*) | 1.27 (1.14 - 1.41) | 9.33E-06 |  | 0.77 (0.63 - 0.93) | 7.59E-03 |  | 1.13 (1.03 - 1.24) | 7.82E-03 | 7.1E-4 |
|  | rs4545118 | A | C | 0.49 | 8q24.3 (*ZNF696*) | 1.27 (1.14 - 1.41) | 9.46E-06 |  | NA | NA |  | 1.27 (1.14 - 1.41) | 9.46E-06 | 0.520 |
| A1 = Minor allele = effect allele, A2 = Major Allele, MAF = minor allele frequency, Region = Chromosomal region and associated gene, OR = odds ratio for minor allele, 95% CI = 95% confidence interval, Het P = P-value for meta-analysis heterogeneity test, Discovery: Meta analysis results for 3 GENIE discovery cohorts. Stage 2: Meta analysis results for 10 replication cohorts. Combined: Meta analysis results for discovery and the 10 stage 2 cohorts. NA= no result, due to genotype failure or quality control filtering. | | | | | | | | | | | | | | |
