# Supplementary material for: New Susceptibility Loci Associated with Kidney Disease in Type 1 Diabetes
Source: PLoS Genet. 2012 Sep 20;8(9):e1002921. doi: 10.1371/journal.pgen.1002921 (PMC3447939; doi:10.1371/journal.pgen.1002921)
Supplement: Table S3 — Gene ontology analysis of all genes within ±1 Mbp of top GWAS signals: rs7583877/AFF3; rs12437854/15q26; rs7588550/ERBB4. (DOC) [file pgen.1002921.s007.doc]

**Table S3. Gene ontology analysis of all genes within ±1Mbp of top GWAS signals: rs7583877/ *AFF3*; rs12437854/ 15q26; rs7588550/ *ERBB4*.**

| **Annotation Cluster 1** | **Enrichment Score: 0.46** | | |
| --- | --- | --- | --- |
| Category | Count | Genes | P Value |
| GO:0032555~purine ribonucleotide binding | 4 | *ERBB4, CHD2, EIF5B, SPAG1* | 0.32 |
| GO:0032553~ribonucleotide binding | 4 | *ERBB4, CHD2, EIF5B, SPAG1* | 0.32 |
| nucleotide-binding | 4 | *ERBB4, CHD2, EIF5B, SPAG1* | 0.33 |
| GO:0017076~purine nucleotide binding | 4 | *ERBB4, CHD2, EIF5B, SPAG1* | 0.34 |
| GO:0000166~nucleotide binding | 4 | *ERBB4, CHD2, EIF5B, SPAG1* | 0.45 |
|  |  |  |  |
| **Annotation Cluster 2** | **Enrichment Score: 0.29** | | |
| Category | Count | Genes | P Value |
| dna-binding | 5 | *NPAS2, IKZF2, REV1, CHD2, AFF3* | 0.18 |
| GO:0003677~DNA binding | 5 | *NPAS2, IKZF2, REV1, CHD2, AFF3* | 0.23 |
| GO:0006357~regulation of transcription from RNA polymerase II | 3 | *NPAS2, IKZF2, CHD2* | 0.27 |
| nucleus | 6 | *NPAS2, IKZF2, REV1, CHD2, TSGA10, AFF3* | 0.61 |
| transcription regulation | 3 | *NPAS2, IKZF2, AFF3* | 0.71 |
| Transcription | 3 | *NPAS2, IKZF2, AFF3* | 0.73 |
| GO:0006355~regulation of transcription, DNA-dependent | 3 | *NPAS2, IKZF2, CHD2* | 0.73 |
| GO:0045449~regulation of transcription | 4 | *NPAS2, IKZF2, CHD2, AFF3* | 0.74 |
| GO:0051252~regulation of RNA metabolic process | 3 | *NPAS2, IKZF2, CHD2* | 0.74 |
| GO:0006350~transcription | 3 | *NPAS2, IKZF2, AFF3* | 0.82 |
|  |  |  |  |
| **Annotation Cluster 3** | **Enrichment Score: 0.24** | | |
| Category | Count | Genes | P Value |
| signal | 6 | *RGMA, FAM174B, ERBB4, LYG2, LYG1, NMS* | 0.35 |
| signal peptide | 6 | *RGMA, FAM174B, ERBB4, LYG2, LYG1, NMS* | 0.35 |
| Secreted | 3 | *LYG2, LYG1, NMS* | 0.61 |
| GO:0005576~extracellular region | 3 | *LYG2, LYG1, NMS* | 0.63 |
| disulfide bond | 3 | *ERBB4, LYG2, LYG1* | 0.87 |
| disulfide bond | 3 | *ERBB4, LYG2, LYG1* | 0.88 |
|  |  |  |  |
| **Annotation Cluster 4** | **Enrichment Score: 0.059** | | |
| Category | Count | Genes | P Value |
| alternative splicing | 8 | *RGMA, MCTP2, IKZF2, REV1, ERBB4, CHD2, MRPL30, LONRF2* | 0.85 |
| splice variant | 8 | *RGMA, MCTP2, IKZF2, REV1, ERBB4, CHD2, MRPL30, LONRF2* | 0.86 |
| GO:0046872~metal ion binding | 4 | *MCTP2, IKZF2, REV1, LONRF2* | 0.87 |
| GO:0043169~cation binding | 4 | *MCTP2, IKZF2, REV1, LONRF2* | 0.88 |
| GO:0043167~ion binding | 4 | *MCTP2, IKZF2, REV1, LONRF2* | 0.89 |
| metal-binding | 3 | *IKZF2, REV1, LONRF2* | 0.89 |
|  |  |  |  |
| **Annotation Cluster 5** | **Enrichment Score: 0.040** | | |
| Category | Count | Genes | P Value |
| membrane | 7 | *RGMA, FAM174B, MCTP2, CHST10, ERBB4, TSGA10, MITD1* | 0.81 |
| GO:0031224~intrinsic to membrane | 5 | *RGMA, FAM174B, MCTP2, CHST10, ERBB4* | 0.88 |
| glycosylation site:N-linked (GlcNAc...) | 4 | *RGMA, FAM174B, CHST10, ERBB4* | 0.90 |
| glycoprotein | 4 | *RGMA, FAM174B, CHST10, ERBB4* | 0.92 |
| topological domain:Cytoplasmic | 3 | *FAM174B, CHST10, ERBB4* | 0.93 |
| GO:0016021~integral to membrane | 4 | *FAM174B, MCTP2, CHST10, ERBB4* | 0.95 |
| transmembrane region | 4 | *FAM174B, MCTP2, CHST10, ERBB4* | 0.96 |
| transmembrane | 4 | *FAM174B, MCTP2, CHST10, ERBB4* | 0.96 |
| *Input gene list of all genes flanking the top three GWAS signals within a 1Mbp window either side of the signal (n=24) for DAVID Gene Ontology Analysis(http://david.abcc.ncifcrf.gov/). Background gene list for comparison was the DAVID default human whole genome background. | | | |
|
|
