# Supplementary material for: New Susceptibility Loci Associated with Kidney Disease in Type 1 Diabetes
Source: PLoS Genet. 2012 Sep 20;8(9):e1002921. doi: 10.1371/journal.pgen.1002921 (PMC3447939; doi:10.1371/journal.pgen.1002921)
Supplement: Table S4 — Gene expression in early DN versus living donor kidney biopsies. All genes within a 2 Mb window (1 Mb upstream and downstream) of the three main signals (rs7583877/AFF3, rs12437854/15q26, rs7588550/ERBB4) were studied. (DOC) [file pgen.1002921.s008.doc]

**Table S4. Gene expression in early DN versus living donor kidney biopsies.** All genes within a 2 Mb window (1 Mb upstream and downstream) of the three main signals (rs7583877/ *AFF3*, rs12437854/ 15q26, rs7588550/ *ERBB4*) were studied.

|  |  |  | Tub (DN=49, LD=32) | | Glom (DN=70, LD=32) | |
| --- | --- | --- | --- | --- | --- | --- |
| GWAS locus | GeneID | Gene | FoldChange | q-value | FoldChange | q-value |
| rs7583877/AFF3 | 80705 | *TSGA10* | **0.93** | **0.03** | **0.91** | **0.03** |
|  | 51601 | *LIPT1* | **1.15** | **0** | **1.24** | **0** |
|  | 10190 | *TXNDC9* | **1.09** | **0.01** | **1.17** | **0** |
|  | 9669 | *EIF5B* | 1.02 | 0.3 | 0.99 | 0.27 |
|  | 51455 | *REV1* | 0.94 | 0.11 | 0.93 | 0.09 |
|  | 3899 | *AFF3* | 1.02 | 0.24 | 0.97 | 0.1 |
|  | 9486 | *CHST10* | 1 | NA | 0.97 | 0.19 |
|  | 4862 | *NPAS2* | 0.94 | 0.09 | **0.93** | **0.01** |
| rs12437854/15q26 | 400451 | *FAM174B* | 0.98 | 0.38 | **0.8** | **0** |
|  | 1106 | *CHD2* | 1.01 | 0.41 | **0.94** | **0.02** |
| rs7588550/ERBB4 | 2066 | *ERBB4* | **0.94** | **0.04** | 0.94 | 0.06 |
|  | 22807 | *IKZF2* | 1.01 | 0.46 | 0.96 | 0.07 |
|  | 79582 | *SPAG16* | **1.11** | **0** | 1 | 0.3 |
| Tub = tubulo-interstitial kidney biopsies, Glom = glomerular kidney biopsies, DN = diabetic nephropathy, LD = living kidney donor, GeneID = NCBI UID, q-value: Q-value for false discovery rate (FDR); Bold: FDR<0.05 | | | | | | |
