# Supplementary material for: New Susceptibility Loci Associated with Kidney Disease in Type 1 Diabetes
Source: PLoS Genet. 2012 Sep 20;8(9):e1002921. doi: 10.1371/journal.pgen.1002921 (PMC3447939; doi:10.1371/journal.pgen.1002921)
Supplement: Table S5 — Significantly enriched pathways (Genomatix Pathway System) for the ERBB4-correlated genes in early diabetic nephropathy. (DOC) [file pgen.1002921.s009.doc]

**Table S5. Significantly enriched pathways (Genomatix Pathway System) for the *ERBB4*-correlated genes in early diabetic nephropathy.**

|  |  | **Pima *ERBB4*-correlated genes** | | |
| --- | --- | --- | --- | --- |
| **Canonical pathway** | **# Genes (total)** | **P-value** | **# Genes (observed)** | **# Genes (expected)** |
| platelet amyloid precursor protein pathway | 13 | 5.31E-07 | 6 | 0.36 |
| intrinsic prothrombin activation pathway | 24 | 3.26E-05 | 6 | 0.66 |
| regulators of bone mineralization | 11 | 1.51E-04 | 4 | 0.30 |
| Regulation of RhoA activity | 45 | 0.001 | 6 | 1.23 |
| Beta1 integrin cell surface interactions | 66 | 0.002 | 7 | 1.81 |
| basic mechanisms of sumoylation | 6 | 0.010 | 2 | 0.16 |
| rac1 cell motility signaling pathway | 36 | 0.016 | 4 | 0.99 |
| Filopodium formation ( Integrin signaling pathway ) | 122 | 0.018 | 8 | 3.35 |
| E-cadherin signaling in keratinocytes | 21 | 0.019 | 3 | 0.58 |
| reversal of insulin resistance by leptin | 10 | 0.029 | 2 | 0.27 |
| carm1 and regulation of the estrogen receptor | 26 | 0.033 | 3 | 0.71 |
| cardiac protection against ros | 11 | 0.035 | 2 | 0.30 |
| control of gene expression by vitamin d receptor | 11 | 0.035 | 2 | 0.30 |
| Posttranslational regulation of adherens junction stability and dissassembly | 48 | 0.041 | 4 | 1.32 |
| vegf hypoxia and angiogenesis | 30 | 0.048 | 3 | 0.82 |
| Pathway analysis was performed with Genomatix pathway System on 388 genes whose gene expression was correlated with that of *ERBB4* in Pima Indians with type 2 diabetes (Benjamin-Hochberg Q-value < 0.1). # Genes = number of genes, total/observed/expected. | | | | |
