# Supplementary material for: New Susceptibility Loci Associated with Kidney Disease in Type 1 Diabetes
Source: PLoS Genet. 2012 Sep 20;8(9):e1002921. doi: 10.1371/journal.pgen.1002921 (PMC3447939; doi:10.1371/journal.pgen.1002921)
Supplement: Table S6 — Cross-sectional and longitudinal analyses in FinnDiane for rs7583877 (AFF3) and rs12437854 (chromosome 15q26). (DOC) [file pgen.1002921.s010.doc]

**Table S6. Cross-sectional and longitudinal analyses in FinnDiane for rs7583877 (*AFF3*) and rs12437854 (chromosome 15q26).**

|  | **rs7583877 /** C | | |  | **rs12437854 /** G | | |
| --- | --- | --- | --- | --- | --- | --- | --- |
| **Case-Control Analysis** | **N (cases / controls)** | **OR (95% CI)** | **P** |  | **N (cases / controls)** | **OR (95 % CI)** | **P** |
| DN | 1319 / 1591 | 1.18 (1.04 - 1.34) | 9.33E-03 |  | 1319 / 1591 | 1.61 (1.14 - 2.28) | 6.11E-03 |
| ESRD vs. non-ESRD | 645 / 2725 | 1.46 (1.27 - 1.68) | 7.87E-08 |  | 645 / 2725 | 1.73 (1.23 - 2.43) | 0.00153 |
| ESRD vs. normoalbuminuria | 645 / 1591 | 1.44 (1.23 - 1.68) | 7.03E-06 |  | 645 / 1591 | 1.94 (1.3 - 2.88) | 0.00134 |
| **Time-to-Event Analysis** | **N (cases / controls)** | **HR (95% CI)** | **P** |  | **N (cases / controls)** | **HR (95 % CI)** | **P** |
| Time from T1D onset to micro | 1002 / 1619 | 1.07 (0.97 - 1.18) | 0.174 |  | 1036 / 1629 | 1.18 (0.92 - 1.51) | 0.201 |
| Time from T1D onset to macro | 907 / 2077 | 1.15 (1.04 - 1.27) | 6.45E-03 |  | 928 / 2102 | 1.31 (1.03 - 1.67) | 0.030 |
| Time from T1D onset to ESRD | 633 / 2636 | 1.33 (1.18 - 1.49) | 1.94E-06 |  | 645 / 2678 | 1.35 (1.02 - 1.77) | 0.034 |
| Time from macro to ESRD | 456 / 433 | 1.16 (1.01 - 1.33) | 0.040 |  | 465 / 439 | 1.16 (0.83 - 1.61) | 0.380 |
| Time from T1D onset to death^a^ | 92 / 2797 | 0.97 (0.7 - 1.36) | 0.879 |  | 94 / 2840 | 0.39 (0.1 - 1.52) | 0.173 |
| Time from macro to death | 266 / 623 | 1.05 (0.87 - 1.26) | 0.600 |  | 272 / 632 | 1.03 (0.66 - 1.62) | 0.883 |
| Time from ESRD to death | 295 / 335 | 1.09 (0.91 - 1.29) | 0.350 |  | 300 / 339 | 0.86 (0.56 - 1.31) | 0.481 |
| Time from incident ESRD to death | 198 / 260 | 1.09 (0.88 – 1.34) | 0.42 |  | 202 / 265 | 0.90 (0.60 – 1.51) | 0.83 |
| The Table shows the results for the longitudinal analyses performed for the top two signals in the FinnDiane discovery cohort. Results for the case-control analyses (DN, ESRD vs. non-ESRD and ESRD vs. normoalbuminuria) are provided for comparison of the *P*-values; all other analyses are time-to-event analyses. All analyses were performed only in the FinnDiane discovery cohort for consistency. All analyses assume an additive model of genetic effect. Kaplan-Meier plots of the time-to-event analyses are shown in Figure S3, with more detail on genotype counts and number of samples at risk at different time points. The allele code after each SNP rs-number indicates the minor and effect allele. N = number of samples in the analysis, OR = odds ratio, HR = hazard ratio, 95% CI = 95% confidence interval. ^a^Subjects that developed DN were censored out at the onset of DN (N=924). | | | | | | | |
