# Supplementary material for: New Susceptibility Loci Associated with Kidney Disease in Type 1 Diabetes
Source: PLoS Genet. 2012 Sep 20;8(9):e1002921. doi: 10.1371/journal.pgen.1002921 (PMC3447939; doi:10.1371/journal.pgen.1002921)
Supplement: Table S7 — Additional kidney phenotype analysis results for the three main loci. (DOC) [file pgen.1002921.s011.doc]

**Table S7. Additional kidney phenotype analysis results for the three main loci.**

|  | **rs7583877 (*AFF3*)** | |  | **rs12437854 (*RGMA* - *MCTP2*)** | |  | **rs7588550 (*ERBB4*)** | |
| --- | --- | --- | --- | --- | --- | --- | --- | --- |
| **Phenotype** | **OR (95%CI)** | **P** |  | **OR (95%CI)** | **P** |  | **OR (95%CI)** | **P** |
| DN | 1.14 (1.05 - 1.25) | 2.26E-03 |  | 1.43 (1.14 - 1.78) | 1.62E-03 |  | 0.65 (0.55 - 0.79) | **5.31E-06** |
| ESRD vs. non-ESRD | 1.34 (1.22 - 1.48) | **4.76E-09** |  | 1.72 (1.36 - 2.18) | **7.65E-06** |  | 0.78 (0.63 - 0.97) | 0.026 |
| ESRD vs. normo | 1.33 (1.19 - 1.48) | 4.87E-07 |  | 1.82 (1.39 - 2.39) | 1.59E-05 |  | 0.67 (0.53 - 0.84) | 7.54E-04 |
| ESRD vs. macro | 1.36 (1.20 - 1.53) | 1.49E-06 |  | 1.57 (1.17 - 2.11) | 2.42E-03 |  | 1.15 (0.86 - 1.54) | 0.34 |
| Macro vs. normo | 1.00 ( 0.90 - 1.11) | 0.95 |  | 1.14 ( 0.86 - 1.50) | 0.35 |  | 0.64 ( 0.51 - 0.81) | 2.19E-04 |
| Association results for various kidney phenotypes for the three top signals. Results are calculated for the meta-analysis of the three discovery cohorts. DN, ESRD vs. non-ESRD and ESRD vs. normo phenotypes have been described in the Methods. Two additional phenotypes were studied for better comparison of various phenotypes: ESRD vs. macro phenotype: cases with ESRD are compared to controls with macroalbuminuria. “Macro vs. normo”: cases with macroalbuminuria are compared to controls with normoalbuminuria. *P*-values with bold font indicate the SNP-phenotype combination that was selected for the second stage evaluation. All the analyses were adjusted for age, T1D duration, sex, ten first PCA components (and possibly the study center). All odds ratios are given considering the minor allele as the effect allele. | | | | | | | | |
