# Supplementary material for: New Susceptibility Loci Associated with Kidney Disease in Type 1 Diabetes
Source: PLoS Genet. 2012 Sep 20;8(9):e1002921. doi: 10.1371/journal.pgen.1002921 (PMC3447939; doi:10.1371/journal.pgen.1002921)
Supplement: Table S8 — P-value for association with DN related traits for the main signals after combined meta-analysis of DN and ESRD phenotypes. A1 is associated with increasing risk of ESRD/DN. (DOC) [file pgen.1002921.s012.doc]

**Table S8. *P*-value for association with DN-related traits for the main signals after combined meta-analysis of DN and ESRD phenotypes**.

|  |  |  |  |  | **MAGIC [1]** | | | |  | **GIANT [2]** | |  | **ICBP [3]** | |  | **Willer *et al.*2008 [4]** | | | |
| --- | --- | --- | --- | --- | --- | --- | --- | --- | --- | --- | --- | --- | --- | --- | --- | --- | --- | --- | --- |
| **Gene** | **SNP** | **A1/A2** | **Freq(A1)** |  | **Fasting glucose** | **Fasting insulin** | **2h glucose** | **HbA1c** |  | **BMI** | **WHR** |  | **SBP** | **DBP** |  | **TC** | **TG** | **HDL** | **LDL** |
| *RGMA* | rs12437854 | G/T | 0.15 |  | **0.028 (+)^a^** | 0.47 | 0.17 | 0.65 |  | - | 0.22 |  | 0.09 | 0.25 |  | 0.41 | **0.016 (-)^b^** | 0.46 | 0.88 |
| *AFF3* | rs7583877 | C/T | 0.33 |  | 0.68 | 0.89 | 0.09 | 0.73 |  | 0.42 | **0.035 (+)^a^** |  | 0.71 | 0.90 |  | 0.77 | 0.95 | 0.29 | 0.16 |
| *ERBB4* | rs7588550 | A/G | 0.95 |  | 0.47 | 0.82 | 0.34 | 0.93 |  | 0.63 | 0.47 |  | 0.28 | 0.33 |  | 0.62 | 0.21 | 0.12 | 0.54 |
| Gene = gene closest to SNP, A1 = Allele associated with increasing risk of DN, Freq(A1) = A1 frequency, BMI = body mass index, WHR = waist to hip ratio, TC = total cholesterol, TG = triglycerides, HDL = high density lipoprotein cholesterol, LDL = low density lipoprotein cholesterol. (+) and (-) signs indicate the direction of association for A1 with the DN-related phenotypes. ^a^ Expected direction of effect given clinical phenotype correlation with DN. ^b^Opposite direction of effect given clinical phenotype correlation with DN. | | | | | | | | | | | | | | | | | | | |

Reference List

1. Dupuis J, Langenberg C, Prokopenko I, Saxena R, Soranzo N, *et al.* (2010) New genetic loci implicated in fasting glucose homeostasis and their impact on type 2 diabetes risk. Nat Genet 42: 105-116.

2. Heid IM, Jackson AU, Randall JC, Winkler TW, Qi L, *et al.* (2010) Meta-analysis identifies 13 new loci associated with waist-hip ratio and reveals sexual dimorphism in the genetic basis of fat distribution. Nat Genet 42: 949-960.

3. Ehret GB, Munroe PB, Rice KM, Bochud M, Johnson AD, *et al.* (2011) Genetic variants in novel pathways influence blood pressure and cardiovascular disease risk. Nature 478: 103-109.

4. Willer CJ, Sanna S, Jackson AU, Scuteri A, Bonnycastle LL, *et al.* (2008) Newly identified loci that influence lipid concentrations and risk of coronary artery disease. Nat Genet 40: 161-169.
