# Supplementary material for: New Susceptibility Loci Associated with Kidney Disease in Type 1 Diabetes
Source: PLoS Genet. 2012 Sep 20;8(9):e1002921. doi: 10.1371/journal.pgen.1002921 (PMC3447939; doi:10.1371/journal.pgen.1002921)
Supplement: Table S9 — GENIE GWAS associations for SNPs that have been previously associated with T1D or chronic kidney disease. (DOC) [file pgen.1002921.s013.doc]

**Table S9**. GENIE GWAS associations for SNPs that have been previously associated with T1D or chronic kidney disease

| **Kidney Disease Signals** | | | | | | | | | | | | | | |
| --- | --- | --- | --- | --- | --- | --- | --- | --- | --- | --- | --- | --- | --- | --- |
|  |  |  |  |  |  |  | **DN** | |  | **ESRD vs. non-ESRD** | |  | **ESRD vs. normo** | |
|  | **Region** | **Gene** | **SNP** | **REF** | **A1** | **A2** | **P** | **OR (95% CI)** |  | **P** | **OR (95% CI)** |  | **P** | **OR (95% CI)** |
| **CKD** | | | | | | | | | | | | | | |
|  | 2p25.2 | *SOX11* | rs16864170 | b | T | C | 0.44 | 1.08 ( 0.89 ± 1.3 ) |  | 0.27 | 1.13 ( 0.91 ± 1.42 ) |  | 0.13 | 1.21 ( 0.94 ± 1.54 ) |
|  | 7q36.1 | *PRKAG2* | rs7805747 | b | A | G | 0.69 | 1.02 ( 0.91 ± 1.14 ) |  | 0.80 | 0.98 ( 0.86 ± 1.12 ) |  | 0.89 | 0.99 ( 0.86 ± 1.14 ) |
|  | 16p21.3 | *UMOD, FLJ20581, GP2, PDILT* | rs12917707 | a,b | T | G | 0.67 | 1.02 ( 0.92 ± 1.13 ) |  | 0.96 | 1 ( 0.89 ± 1.12 ) |  | 0.75 | 1.02 ( 0.9 ± 1.16 ) |
| **eGFRcys** | | | | | | | | | | | | | | |
|  | 8p21.2 | *STC1* | rs1731274 | a | A | G | 0.63 | 0.98 ( 0.91 ± 1.06 ) |  | 0.32 | 0.95 ( 0.87 ± 1.05 ) |  | 0.29 | 0.95 ( 0.85 ± 1.05 ) |
|  | 12q24.12 | *ATXN2* | rs653178 | b | T | C | 0.88 | 0.99 ( 0.92 ± 1.08 ) |  | 0.66 | 0.98 ( 0.89 ± 1.08 ) |  | 0.91 | 0.99 ( 0.9 ± 1.1 ) |
|  | 16p12.3 | *UMOD, FLJ20581, GP2, PDILT* | rs12917707 | a,b | T | G | 0.67 | 1.02 ( 0.92 ± 1.13 ) |  | 0.96 | 1 ( 0.89 ± 1.12 ) |  | 0.75 | 1.02 ( 0.9 ± 1.16 ) |
|  | 20p11.21 | *CST3, CST9* | rs13038305 | a | T | C | 0.54 | 1.03 ( 0.94 ± 1.13 ) |  | 0.31 | 1.06 ( 0.95 ± 1.18 ) |  | 0.32 | 1.06 ( 0.94 ± 1.2 ) |
|  | 20p11.21 | *CST3, CST4, CST9* | rs911119 | b | T | C | 0.51 | 0.97 ( 0.88 ± 1.06 ) |  | 0.30 | 0.94 ( 0.85 ± 1.05 ) |  | 0.31 | 0.94 ( 0.83 ± 1.06 ) |
| **eGFRcrea** | | | | | | | | | | | | | | |
|  | 1p13.3 | *SYPL2, ATXN7L2, CYB561D1, PSMA5, AMIGO1, SORT1* | rs1933182 | b | A | C | 0.54 | 1.03 ( 0.94 ± 1.12 ) |  | 0.47 | 1.04 ( 0.94 ± 1.15 ) |  | 0.60 | 1.03 ( 0.92 ± 1.15 ) |
|  | 1q21.3 | *ANXA9, FAM63A, PRUNE, BNIPL, LASS2, SETDB1* | rs267734 | b | T | C | 0.53 | 0.97 ( 0.88 ± 1.07 ) |  | 0.75 | 0.98 ( 0.88 ± 1.1 ) |  | 0.74 | 0.98 ( 0.86 ± 1.11 ) |
|  | 2p13.1 | *NAT8, NAT8B, ALMS1* | rs13538 | b | A | G | 0.70 | 1.02 ( 0.92 ± 1.12 ) |  | 0.92 | 1.01 ( 0.9 ± 1.13 ) |  | 0.97 | 1 ( 0.88 ± 1.13 ) |
|  | 2p23.3 | *GCKR, IFT172, FNDC4* | rs1260326 | b | T | C | 0.29 | 0.96 ( 0.88 ± 1.04 ) |  | 0.68 | 0.98 ( 0.89 ± 1.08 ) |  | 0.30 | 0.95 ( 0.85 ± 1.05 ) |
|  | 2q34 | *CPS1* | rs7422339 | b | A | C | 0.32 | 0.95 ( 0.87 ± 1.05 ) |  | 0.87 | 0.99 ( 0.89 ± 1.11 ) |  | 0.41 | 0.95 ( 0.84 ± 1.07 ) |
|  | 3q23 | *TFDP2* | rs347685 | b | A | C | 0.25 | 0.95 ( 0.87 ± 1.04 ) |  | 0.20 | 0.93 ( 0.84 ± 1.04 ) |  | 0.15 | 0.92 ( 0.82 ± 1.03 ) |

**Table S9.** Continued

| **Kidney Disease Signals continued** | | | | | | | | | | | | | | |
| --- | --- | --- | --- | --- | --- | --- | --- | --- | --- | --- | --- | --- | --- | --- |
|  |  |  |  |  |  |  | **DN** | |  | **ESRD vs. non-ESRD** | |  | **ESRD vs. normo** | |
|  | **Region** | **Gene** | **SNP** | **REF** | **A1** | **A2** | **P** | **OR (95% CI)** |  | **P** | **OR (95% CI)** |  | **P** | **OR (95% CI)** |
| **eGFRcrea** | | | | | | | | | | | | | | |
|  | 4q21.1 | *SHROOM3, FLJ25770* | rs17319721 | a,b | A | G | 0.57 | 1.02 ( 0.95 ± 1.11 ) |  | 0.69 | 1.02 ( 0.93 ± 1.12 ) |  | 0.71 | 1.02 ( 0.92 ± 1.13 ) |
|  | 5p13.1 | *DAB2, C9* | rs11959928 | b | A | T | 0.78 | 1.01 ( 0.93 ± 1.1 ) |  | 0.94 | 1 ( 0.91 ± 1.1 ) |  | 0.78 | 1.01 ( 0.91 ± 1.13 ) |
|  | 5q35.3 | *SLC34A1, GRK6, RGS14, LMAN2, PRR7, F12, PFN3* | rs6420094 | b | A | G | 0.30 | 0.96 ( 0.88 ± 1.04 ) |  | 0.39 | 0.96 ( 0.86 ± 1.06 ) |  | 0.19 | 0.93 ( 0.83 ± 1.04 ) |
|  | 6q21.1 | *VEGFA* | rs881858 | b | A | G | 0.68 | 1.02 ( 0.93 ± 1.12 ) |  | 0.42 | 1.05 ( 0.94 ± 1.17 ) |  | 0.77 | 1.02 ( 0.9 ± 1.15 ) |
|  | 6q25.3 | *SLC22A2* | rs2279463 | b | A | G | 0.11 | 0.91 ( 0.81 ± 1.02 ) |  | 0.74 | 0.98 ( 0.85 ± 1.12 ) |  | 0.47 | 0.95 ( 0.81 ± 1.1 ) |
|  | 7q11.23 | *TMEM60, RSBN1L, PHTF2* | rs6465825 | b | T | C | 0.26 | 1.05 ( 0.97 ± 1.14 ) |  | 0.85 | 1.01 ( 0.92 ± 1.11 ) |  | 0.53 | 1.03 ( 0.93 ± 1.15 ) |
|  | 8p21.2 | *STC1* | rs10109414 | b | T | C | 0.94 | 1 ( 0.93 ± 1.09 ) |  | 0.44 | 1.04 ( 0.95 ± 1.14 ) |  | 0.45 | 1.04 ( 0.94 ± 1.15 ) |
|  | 9q21.11 | *PIP5K1B, FAM122A* | rs4744712 | b | A | C | 0.17 | 0.95 ( 0.87 ± 1.02 ) |  | 0.14 | 0.93 ( 0.85 ± 1.02 ) |  | 0.11 | 0.92 ( 0.83 ± 1.02 ) |
|  | 10p15.3 | *WDR37* | rs10794720 | b | T | C | 0.76 | 1.02 ( 0.88 ± 1.19 ) |  | 0.84 | 1.02 ( 0.85 ± 1.22 ) |  | 0.67 | 1.04 ( 0.86 ± 1.27 ) |
|  | 11q13.1 | *RNASEH2C, DKFZp761E19 8, HTATIP, OVOL1* | rs4014195 | b | C | G | 0.63 | 1.02 ( 0.94 ± 1.1 ) |  | 0.37 | 0.96 ( 0.87 ± 1.05 ) |  | 0.66 | 0.98 ( 0.88 ± 1.08 ) |
|  | 12p13.33 | *SLC6A13, JARID1A, SLC6A12* | rs10774021 | b | T | C | 0.69 | 0.98 ( 0.9 ± 1.07 ) |  | 0.71 | 0.98 ( 0.89 ± 1.08 ) |  | 0.66 | 0.98 ( 0.88 ± 1.09 ) |
|  | 13q21.33 | *DACH1* | rs626277 | b | A | C | 0.30 | 0.96 ( 0.88 ± 1.04 ) |  | 0.93 | 1 ( 0.9 ± 1.1 ) |  | 0.73 | 0.98 ( 0.88 ± 1.09 ) |
|  | 15q21.1 | *GATM, SPATA5L1* | rs2453533 | b | A | C | 0.27 | 1.05 ( 0.97 ± 1.13 ) |  | 0.74 | 1.02 ( 0.92 ± 1.12 ) |  | 0.37 | 1.05 ( 0.95 ± 1.16 ) |
|  | 15q21.1 | *SPATA5L1, GATM* | rs2467853 | a | T | G | 0.37 | 0.96 ( 0.89 ± 1.05 ) |  | 0.64 | 0.98 ( 0.89 ± 1.08 ) |  | 0.36 | 0.95 ( 0.86 ± 1.06 ) |
|  | 15q21.3 | *WDR72* | rs491567 | b | A | C | 0.78 | 0.99 ( 0.9 ± 1.08 ) |  | 0.66 | 0.98 ( 0.88 ± 1.08 ) |  | 0.88 | 0.99 ( 0.88 ± 1.11 ) |
|  | 15q24.2 | *UBE2Q2, FBXO22* | rs1394125 | b | A | G | 0.27 | 1.05 ( 0.96 ± 1.16 ) |  | 0.19 | 1.08 ( 0.96 ± 1.2 ) |  | 0.11 | 1.11 ( 0.98 ± 1.25 ) |
|  | 16p12.3 | *UMOD, FLJ20581, GP2, PDILT* | rs12917707 | a,b | T | G | 0.67 | 1.02 ( 0.92 ± 1.13 ) |  | 0.96 | 1 ( 0.89 ± 1.12 ) |  | 0.75 | 1.02 ( 0.9 ± 1.16 ) |
|  | 17q23.2 | *BCAS3, TBX2, C17orf82* | rs9895661 | b | T | C | 0.61 | 1.03 ( 0.92 ± 1.15 ) |  | 0.12 | 1.11 ( 0.97 ± 1.26 ) |  | 0.20 | 1.1 ( 0.95 ± 1.27 ) |
|  | 19q13.11 | *SLC7A9, CCDC123, ECAT8* | rs12460876 | b | T | C | 0.21 | 0.95 ( 0.88 ± 1.03 ) |  | 0.98 | 1 ( 0.91 ± 1.1 ) |  | 0.83 | 0.99 ( 0.89 ± 1.1 ) |
|  | 20p12.2 | *JAG1* | rs6040055 | a | T | C | 0.68 | 1.02 ( 0.93 ± 1.11 ) |  | 0.38 | 1.05 ( 0.94 ± 1.16 ) |  | 0.43 | 1.05 ( 0.93 ± 1.18 ) |

**Table S9.** Continued

| **Kidney Disease Signals continued** | | | | | | | | | | | | | | |
| --- | --- | --- | --- | --- | --- | --- | --- | --- | --- | --- | --- | --- | --- | --- |
|  |  |  |  |  |  |  | **DN** | |  | **ESRD vs. non-ESRD** | |  | **ESRD vs. normo** | |
|  | **Region** | **Gene** | **SNP** | **REF** | **A1** | **A2** | **P** | **OR (95% CI)** |  | **P** | **OR (95% CI)** |  | **P** | **OR (95% CI)** |
| **Serum creatinine** | | | | | | | | | | | | | | |
|  | 2p12-p13 | *NAT8* | rs10206899 | c | G | A | 0.72 | 0.98 ( 0.89 - 1.08 ) |  | 0.91 | 0.99 ( 0.88 - 1.11 ) |  | 0.97 | 1.00 ( 0.88 - 1.13 ) |
|  | 6q26 | *SLC22A* | rs3127573 | c | G | A | 0.14 | 1.09 ( 0.97 - 1.22 ) |  | 0.99 | 1.00 ( 0.87 - 1.14 ) |  | 0.67 | 1.03 ( 0.88 - 1.20 ) |
|  | 17q23 | *TBX2* | rs8068318 | c | G | A | 0.65 | 1.02 ( 0.93 - 1.11 ) |  | 0.23 | 0.93 ( 0.84 - 1.04 ) |  | 0.70 | 0.97 ( 0.86 - 1.09 ) |
|  | 19q13 | *SLC7A9* | rs4805834 | c | A | G | 0.11 | 1.09 ( 0.97 - 1.23 ) |  | 0.40 | 0.94 ( 0.82 - 1.08 ) |  | 0.93 | 0.99 ( 0.85 - 1.15 ) |
| **Albuminuria** | | | | | | | | | | | | | | |
|  | 10p13 | *CUBN* | rs1801239 | d | C | T | 0.16 | 1.10 ( 0.96 - 1.27 ) |  | 0.97 | 1.00 ( 0.84 - 1.18 ) |  | 0.43 | 1.07 ( 0.89 - 1.30 ) |

**Table S9.** Continued

| **T1D Signals** | | | | | | | | | | | | | | |
| --- | --- | --- | --- | --- | --- | --- | --- | --- | --- | --- | --- | --- | --- | --- |
|  |  |  |  |  |  |  | **DN** | |  | **ESRD vs. non-ESRD** | |  | **ESRD vs. normo** | |
|  | **Region** | **Gene** | **SNP** | **REF** | **A1** | **A2** | **P** | **OR (95% CI)** |  | **P** | **OR (95% CI)** |  | **P** | **OR (95% CI)** |
|  | 1p13.2 | *PTPN22* | rs2476601 | e | A | G | 0.45 | 0.96 ( 0.87 ± 1.06 ) |  | 0.85 | 0.99 ( 0.88 ± 1.11 ) |  | 0.88 | 0.99 ( 0.87 ± 1.12 ) |
|  | 1q31.2 | *RGS1* | rs2760524 | e | A | G | 0.76 | 1.02 ( 0.91 ± 1.14 ) |  | 0.58 | 1.04 ( 0.91 ± 1.18 ) |  | 0.42 | 1.06 ( 0.92 ± 1.22 ) |
|  | 1q31.2 | *RGS1* | rs2816316 | e | A | C | 0.91 | 0.99 ( 0.89 ± 1.11 ) |  | 0.58 | 0.97 ( 0.85 ± 1.09 ) |  | 0.47 | 0.95 ( 0.83 ± 1.09 ) |
|  | 1q32.1 | *IL10, CD55* | rs3024505 | e | A | G | 0.83 | 1.01 ( 0.9 ± 1.14 ) |  | 0.83 | 1.02 ( 0.88 ± 1.17 ) |  | 0.93 | 1.01 ( 0.86 ± 1.18 ) |
|  | 2p23.3 |  | rs478222 | e | A | T | 0.53 | 1.03 ( 0.95 ± 1.11 ) |  | 0.85 | 0.99 ( 0.9 ± 1.09 ) |  | 0.92 | 1.01 ( 0.91 ± 1.11 ) |
|  | 2q11.2 | *AFF3* | rs10865035 | e | A | G | 0.07 | 1.07 ( 0.99 ± 1.16 ) |  | **0.005** | 1.14 ( 1.04 ± 1.25 ) |  | **0.01** | 1.14 ( 1.03 ± 1.26 ) |
|  | 2q11.2 | *AFF3* | rs1160542 | e | A | G | **0.05** | 0.92 ( 0.86 ± 1 ) |  | **0.003** | 0.87 ( 0.8 ± 0.95 ) |  | **0.01** | 0.87 ( 0.79 ± 0.96 ) |
|  | 2q11.2 | *AFF3* | rs9653442 | e | T | C | **0.05** | 0.92 ( 0.86 ± 1 ) |  | **0.003** | 0.87 ( 0.8 ± 0.95 ) |  | **0.005** | 0.87 ( 0.78 ± 0.96 ) |
|  | 2q24.2 | *IFIH1* | rs1990760 | e | T | C | 0.23 | 0.95 ( 0.88 ± 1.03 ) |  | 0.94 | 1 ( 0.91 ± 1.11 ) |  | 0.42 | 0.96 ( 0.86 ± 1.07 ) |
|  | 2q32.2 | *STAT4* | rs3821236 | e | A | G | 0.53 | 0.97 ( 0.89 ± 1.06 ) |  | 0.52 | 0.97 ( 0.87 ± 1.08 ) |  | 0.37 | 0.95 ( 0.84 ± 1.07 ) |
|  | 2q32.2 | *STAT4* | rs6752770 | e | A | G | 0.30 | 1.05 ( 0.95 ± 1.16 ) |  | 0.17 | 0.92 ( 0.82 ± 1.03 ) |  | 0.53 | 0.96 ( 0.85 ± 1.09 ) |
|  | 2q32.2 | *STAT4* | rs7574865 | e | T | G | 0.96 | 1 ( 0.91 ± 1.1 ) |  | 0.65 | 1.02 ( 0.92 ± 1.14 ) |  | 0.85 | 1.01 ( 0.9 ± 1.14 ) |
|  | 2q32.2 | *STAT4* | rs7582694 | e | C | G | 0.98 | 1 ( 0.91 ± 1.1 ) |  | 0.63 | 1.03 ( 0.92 ± 1.15 ) |  | 0.85 | 1.01 ( 0.9 ± 1.14 ) |
|  | 2q33.2 | *CTLA4* | rs3087243 | e | A | G | 0.17 | 1.06 ( 0.98 ± 1.15 ) |  | 0.28 | 1.05 ( 0.96 ± 1.16 ) |  | 0.35 | 1.05 ( 0.95 ± 1.17 ) |
|  | 3p21.31 | *CCR5* | rs11711054 | e | A | G | 0.47 | 0.97 ( 0.89 ± 1.05 ) |  | 0.21 | 0.94 ( 0.85 ± 1.04 ) |  | 0.16 | 0.93 ( 0.83 ± 1.03 ) |
|  | 4p15.2 |  | rs10517086 | e | A | G | **0.03** | 1.1 ( 1.01 ± 1.2 ) |  | 0.10 | 1.09 ( 0.98 ± 1.2 ) |  | **0.04** | 1.12 ( 1 ± 1.25 ) |
|  | 4q27 | *IL2, IL21* | rs17388568 | e | A | G | 0.33 | 0.96 ( 0.88 ± 1.04 ) |  | 0.30 | 0.95 ( 0.86 ± 1.05 ) |  | 0.29 | 0.94 ( 0.85 ± 1.05 ) |
|  | 4q27 | *IL2, IL21* | rs2069762 | e | A | C | 0.55 | 1.03 ( 0.94 ± 1.12 ) |  | 0.83 | 0.99 ( 0.89 ± 1.1 ) |  | 0.85 | 0.99 ( 0.88 ± 1.11 ) |
|  | 4q27 | *IL2, IL21* | rs4505848 | e | A | G | 0.51 | 1.03 ( 0.95 ± 1.11 ) |  | 0.53 | 1.03 ( 0.94 ± 1.13 ) |  | 0.47 | 1.04 ( 0.94 ± 1.16 ) |
|  | 6q15 | *BACH2* | rs10806425 | e | A | C | **0.04** | 1.09 ( 1.01 ± 1.18 ) |  | 0.85 | 0.99 ( 0.9 ± 1.09 ) |  | 0.61 | 1.03 ( 0.92 ± 1.14 ) |
|  | 6q15 | *BACH2* | rs11755527 | e | C | G | 0.11 | 0.94 ( 0.87 ± 1.02 ) |  | 0.76 | 1.01 ( 0.92 ± 1.11 ) |  | 0.77 | 0.98 ( 0.89 ± 1.09 ) |
|  | 6q22.32 | | rs9388489 | e | A | G | 0.96 | 1 ( 0.93 ± 1.08 ) |  | 0.34 | 0.96 ( 0.87 ± 1.05 ) |  | 0.95 | 1 ( 0.9 ± 1.1 ) |
|  | 6q23.3 | *TNFAIP3* | rs10499194 | e | T | C | 0.29 | 0.95 ( 0.87 ± 1.04 ) |  | 0.82 | 0.99 ( 0.89 ± 1.1 ) |  | 0.69 | 0.98 ( 0.87 ± 1.1 ) |
|  | 6q23.3 | *TNFAIP3* | rs2327832 | e | A | G | 0.85 | 1.01 ( 0.92 ± 1.11 ) |  | 0.74 | 0.98 ( 0.88 ± 1.1 ) |  | 1.00 | 1 ( 0.89 ± 1.13 ) |
|  | 6q23.3 | *TNFAIP3* | rs6920220 | e | A | G | 0.95 | 1 ( 0.91 ± 1.1 ) |  | 0.66 | 1.02 ( 0.92 ± 1.14 ) |  | 0.85 | 1.01 ( 0.9 ± 1.14 ) |
|  | 6q25.3 | *TAGAP* | rs1738074 | e | T | C | 0.71 | 1.02 ( 0.94 ± 1.1 ) |  | 0.76 | 0.99 ( 0.89 ± 1.09 ) |  | 0.94 | 1 ( 0.9 ± 1.12 ) |
|  | 6q27 |  | rs924043 | e | T | C | 0.47 | 1.04 ( 0.93 ± 1.16 ) |  | 0.99 | 1 ( 0.88 ± 1.14 ) |  | 0.93 | 1.01 ( 0.87 ± 1.16 ) |

**Table S9.** Continued

| **T1D Signals continued** | | | | | | | | | | | | | | |
| --- | --- | --- | --- | --- | --- | --- | --- | --- | --- | --- | --- | --- | --- | --- |
|  |  |  |  |  |  |  | **DN** | |  | **ESRD vs. non-ESRD** | |  | **ESRD vs. normo** | |
|  | **Region** | **Gene** | **SNP** | **REF** | **A1** | **A2** | **P** | **OR (95% CI)** |  | **P** | **OR (95% CI)** |  | **P** | **OR (95% CI)** |
|  | 7p12.1 |  | rs4948088 | e | A | C | 0.47 | 0.92 ( 0.72 ± 1.16 ) |  | 0.63 | 0.93 ( 0.7 ± 1.24 ) |  | 0.60 | 0.92 ( 0.67 ± 1.25 ) |
|  | 7p12.2 | *IKZF1* | rs10272724 | e | T | C | 0.70 | 0.98 ( 0.9 ± 1.07 ) |  | 0.89 | 0.99 ( 0.9 ± 1.1 ) |  | 0.64 | 0.97 ( 0.87 ± 1.09 ) |
|  | 7p15.2 | *SKAP2* | rs7804356 | e | T | C | 0.91 | 0.99 ( 0.9 ± 1.1 ) |  | 0.40 | 0.95 ( 0.85 ± 1.07 ) |  | 0.44 | 0.95 ( 0.84 ± 1.08 ) |
|  | 9p24.2 | *GLIS3* | rs7020673 | e | C | G | 0.44 | 0.97 ( 0.9 ± 1.05 ) |  | 0.80 | 1.01 ( 0.92 ± 1.11 ) |  | 0.89 | 0.99 ( 0.9 ± 1.1 ) |
|  | 10p15.1 | *IL2RA* | rs11594656 | e | A | T | 0.60 | 0.97 ( 0.88 ± 1.08 ) |  | 0.47 | 0.96 ( 0.85 ± 1.08 ) |  | 0.67 | 0.97 ( 0.85 ± 1.11 ) |
|  | 10p15.1 | *IL2RA* | rs12251307 | e | T | C | 0.24 | 1.07 ( 0.95 ± 1.21 ) |  | 0.58 | 0.96 ( 0.84 ± 1.1 ) |  | 0.73 | 0.97 ( 0.84 ± 1.13 ) |
|  | 10p15.1 | *IL2RA* | rs2104286 | e | T | C | 0.74 | 1.02 ( 0.92 ± 1.12 ) |  | 0.52 | 1.04 ( 0.93 ± 1.16 ) |  | 0.37 | 1.06 ( 0.94 ± 1.2 ) |
|  | 10p15.1 | *PRKCQ* | rs11258747 | e | T | G | 0.26 | 0.95 ( 0.87 ± 1.04 ) |  | 0.43 | 0.96 ( 0.86 ± 1.06 ) |  | 0.46 | 0.96 ( 0.86 ± 1.07 ) |
|  | 10p15.1 | *PRKCQ* | rs947474 | e | A | G | 0.19 | 1.07 ( 0.97 ± 1.19 ) |  | 0.77 | 0.98 ( 0.87 ± 1.11 ) |  | 0.55 | 1.04 ( 0.91 ± 1.19 ) |
|  | 10q22.3 | *ZMIZ1* | rs1250550 | e | A | C | 0.26 | 1.05 ( 0.96 ± 1.14 ) |  | 0.62 | 1.03 ( 0.93 ± 1.13 ) |  | 0.39 | 1.05 ( 0.94 ± 1.17 ) |
|  | 10q22.3 | *ZMIZ1* | rs1250552 | e | A | G | 0.10 | 0.93 ( 0.86 ± 1.01 ) |  | 0.14 | 0.93 ( 0.84 ± 1.02 ) |  | 0.05 | 0.9 ( 0.81 ± 1 ) |
|  | 10q22.3 | *ZMIZ1* | rs1250558 | e | A | G | 0.31 | 1.04 ( 0.96 ± 1.13 ) |  | 0.39 | 1.04 ( 0.95 ± 1.15 ) |  | 0.25 | 1.07 ( 0.96 ± 1.19 ) |
|  | 10q23.31 | *RNLS* | rs10509540 | e | T | C | 0.68 | 1.02 ( 0.93 ± 1.12 ) |  | 0.75 | 0.98 ( 0.88 ± 1.09 ) |  | 0.89 | 1.01 ( 0.9 ± 1.14 ) |
|  | 12p13.31 | | rs4763879 | e | A | G | 0.55 | 0.98 ( 0.9 ± 1.06 ) |  | 0.41 | 0.96 ( 0.87 ± 1.06 ) |  | 0.49 | 0.96 ( 0.87 ± 1.07 ) |
|  | 12q13.2 | | rs2292239 | e | T | G | 0.18 | 0.95 ( 0.87 ± 1.03 ) |  | 0.55 | 0.97 ( 0.88 ± 1.07 ) |  | 0.56 | 0.97 ( 0.87 ± 1.08 ) |
|  | 12q13.3 | *CYP27B1* | rs10877015 | e | A | G | **0.03** | 0.91 ( 0.84 ± 0.99 ) |  | 0.72 | 0.98 ( 0.89 ± 1.08 ) |  | 0.31 | 0.94 ( 0.85 ± 1.05 ) |
|  | 12q13.3 | *CYP27B1* | rs703842 | e | A | G | **0.03** | 0.91 ( 0.84 ± 0.99 ) |  | 0.72 | 0.98 ( 0.89 ± 1.08 ) |  | 0.32 | 0.95 ( 0.85 ± 1.05 ) |
|  | 12q24.12 | *SH2B3* | rs3184504 | e | T | C | 0.90 | 1 ( 0.93 ± 1.09 ) |  | 0.67 | 1.02 ( 0.93 ± 1.12 ) |  | 0.91 | 1.01 ( 0.91 ± 1.11 ) |
|  | 12q24.12 | *SH2B3* | rs653178 | e | T | C | 0.88 | 0.99 ( 0.92 ± 1.08 ) |  | 0.66 | 0.98 ( 0.89 ± 1.08 ) |  | 0.91 | 0.99 ( 0.9 ± 1.1 ) |
|  | 13q22.2 | | rs539514 | e | A | T | 0.09 | 0.93 ( 0.86 ± 1.01 ) |  | 0.35 | 0.96 ( 0.87 ± 1.05 ) |  | 0.21 | 0.94 ( 0.85 ± 1.04 ) |
|  | 13q32.3 | *GPR183* | rs9585056 | e | T | C | 0.30 | 1.05 ( 0.96 ± 1.14 ) |  | 0.49 | 0.96 ( 0.87 ± 1.07 ) |  | 0.66 | 0.97 ( 0.87 ± 1.09 ) |
|  | 14q24.1 | | rs1465788 | e | T | C | 0.20 | 1.06 ( 0.97 ± 1.15 ) |  | 0.60 | 0.97 ( 0.88 ± 1.08 ) |  | 0.89 | 1.01 ( 0.9 ± 1.13 ) |
|  | 14q32.2 | *DLK1* | rs941576 | e | A | G | 0.50 | 0.97 ( 0.9 ± 1.06 ) |  | 0.76 | 0.99 ( 0.89 ± 1.09 ) |  | 0.62 | 0.97 ( 0.87 ± 1.08 ) |
|  | 14q32.2 | | rs4900384 | e | A | G | **0.02** | 1.1 ( 1.01 ± 1.2 ) |  | 0.32 | 1.05 ( 0.95 ± 1.16 ) |  | 0.08 | 1.1 ( 0.99 ± 1.22 ) |
|  | 15q14 | *RASGRP1* | rs17574546 | e | A | C | 0.53 | 0.97 ( 0.88 ± 1.07 ) |  | 0.75 | 1.02 ( 0.91 ± 1.14 ) |  | 0.96 | 1 ( 0.89 ± 1.14 ) |
|  | 15q14 | *RASGRP1* | rs7171171 | e | A | G | 0.49 | 0.97 ( 0.88 ± 1.06 ) |  | 0.76 | 1.02 ( 0.91 ± 1.14 ) |  | 0.99 | 1 ( 0.89 ± 1.13 ) |
|  | 15q25.1 | | rs3825932 | e | T | C | 0.44 | 0.97 ( 0.89 ± 1.05 ) |  | 0.46 | 0.96 ( 0.87 ± 1.06 ) |  | 0.38 | 0.95 ( 0.85 ± 1.06 ) |

**Table S9.** Continued

| **T1D Signals continued** | | | | | | | | | | | | | | |
| --- | --- | --- | --- | --- | --- | --- | --- | --- | --- | --- | --- | --- | --- | --- |
|  |  |  |  |  |  |  | **DN** | |  | **ESRD vs. non-ESRD** | |  | **ESRD vs. normo** | |
|  | **Region** | **Gene** | **SNP** | **REF** | **A1** | **A2** | **P** | **OR (95% CI)** |  | **P** | **OR (95% CI)** |  | **P** | **OR (95% CI)** |
|  | 16p11.2 | *IL27* | rs151181 | e | T | C | 0.28 | 0.96 ( 0.88 ± 1.04 ) |  | 0.98 | 1 ( 0.91 ± 1.1 ) |  | 0.60 | 0.97 ( 0.87 ± 1.08 ) |
|  | 16p11.2 | *IL27* | rs4788084 | e | T | C | 0.33 | 1.04 ( 0.96 ± 1.13 ) |  | 0.82 | 0.99 ( 0.9 ± 1.09 ) |  | 0.73 | 1.02 ( 0.92 ± 1.13 ) |
|  | 16p11.2 | *IL27* | rs8049439 | e | T | C | 0.45 | 0.97 ( 0.9 ± 1.05 ) |  | 0.90 | 0.99 ( 0.91 ± 1.09 ) |  | 0.51 | 0.97 ( 0.87 ± 1.07 ) |
|  | 16p13.13 | | rs12599402 | e | T | C | 0.75 | 1.01 ( 0.93 ± 1.1 ) |  | 0.82 | 0.99 ( 0.9 ± 1.09 ) |  | 0.92 | 1.01 ( 0.91 ± 1.11 ) |
|  | 16p13.13 | | rs12708716 | e | A | G | 0.79 | 0.99 ( 0.91 ± 1.08 ) |  | 0.32 | 0.95 ( 0.86 ± 1.05 ) |  | 0.39 | 0.95 ( 0.85 ± 1.06 ) |
|  | 16p13.13 | | rs12927773 | e | T | G | 0.81 | 1.01 ( 0.91 ± 1.13 ) |  | 0.29 | 0.93 ( 0.82 ± 1.06 ) |  | 0.43 | 0.94 ( 0.82 ± 1.09 ) |
|  | 16p13.13 | | rs12928822 | e | T | C | 0.81 | 1.01 ( 0.91 ± 1.13 ) |  | 0.29 | 0.93 ( 0.82 ± 1.06 ) |  | 0.43 | 0.94 ( 0.82 ± 1.09 ) |
|  | 16q23.1 | | rs7202877 | e | T | G | 0.14 | 1.09 ( 0.97 ± 1.23 ) |  | 0.54 | 1.04 ( 0.91 ± 1.2 ) |  | 0.32 | 1.08 ( 0.93 ± 1.26 ) |
|  | 17q12 | *ORMDL3, GSDMB* | rs2290400 | e | T | C | 0.18 | 0.95 ( 0.88 ± 1.03 ) |  | 0.81 | 0.99 ( 0.9 ± 1.08 ) |  | 0.55 | 0.97 ( 0.88 ± 1.07 ) |
|  | 17q12 | *ORMDL3, GSDMB* | rs2872507 | e | A | G | 0.42 | 1.03 ( 0.96 ± 1.12 ) |  | 0.77 | 0.99 ( 0.9 ± 1.08 ) |  | 0.98 | 1 ( 0.91 ± 1.11 ) |
|  | 17q21.2 | | rs7221109 | e | T | C | 0.78 | 0.99 ( 0.91 ± 1.07 ) |  | 0.17 | 0.93 ( 0.85 ± 1.03 ) |  | 0.12 | 0.92 ( 0.83 ± 1.02 ) |
|  | 18p11.21 | *PTPN2* | rs1893217 | e | A | G | 0.97 | 1 ( 0.91 ± 1.1 ) |  | 0.29 | 1.06 ( 0.95 ± 1.2 ) |  | 0.65 | 1.03 ( 0.91 ± 1.17 ) |
|  | 18p11.21 | *PTPN2* | rs478582 | e | T | C | 0.85 | 0.99 ( 0.92 ± 1.07 ) |  | 0.71 | 0.98 ( 0.9 ± 1.08 ) |  | 0.39 | 0.96 ( 0.86 ± 1.06 ) |
|  | 18q22.2 | *CD226* | rs763361 | e | T | C | 0.43 | 0.97 ( 0.89 ± 1.05 ) |  | 0.25 | 0.95 ( 0.86 ± 1.04 ) |  | 0.43 | 0.96 ( 0.86 ± 1.07 ) |
|  | 19q13.32 | | rs425105 | e | T | C | 0.55 | 0.97 ( 0.86 ± 1.08 ) |  | 0.63 | 1.03 ( 0.91 ± 1.18 ) |  | 0.93 | 1.01 ( 0.87 ± 1.16 ) |
|  | 19q13.4 | *FUT2* | rs602662 | e | A | G | **0.05** | 0.92 ( 0.84 ± 1 ) |  | 0.56 | 0.97 ( 0.88 ± 1.07 ) |  | 0.23 | 0.93 ( 0.84 ± 1.04 ) |
|  | 20p13 |  | rs2281808 | e | T | C | 0.82 | 1.01 ( 0.93 ± 1.1 ) |  | 0.76 | 0.98 ( 0.89 ± 1.09 ) |  | 0.81 | 0.99 ( 0.88 ± 1.1 ) |
|  | 21q22.3 | *AIRE* | rs760426 | e | A | G | 0.34 | 1.06 ( 0.94 ± 1.2 ) |  | **0.02** | 1.2 ( 1.03 ± 1.39 ) |  | **0.05** | 1.18 ( 1 ± 1.39 ) |
|  | 21q22.3 | *UBASH3A* | rs11203203 | e | A | G | 0.90 | 1 ( 0.92 ± 1.08 ) |  | 0.83 | 0.99 ( 0.9 ± 1.09 ) |  | 0.85 | 0.99 ( 0.89 ± 1.1 ) |
|  | 21q22.3 | *UBASH3A* | rs3788013 | e | A | C | 0.21 | 0.95 ( 0.87 ± 1.03 ) |  | 0.80 | 0.99 ( 0.9 ± 1.09 ) |  | 0.42 | 0.96 ( 0.86 ± 1.07 ) |
|  | 22q12.2 | | rs2412973 | e | A | C | 0.89 | 1.01 ( 0.93 ± 1.09 ) |  | **0.02** | 0.9 ( 0.82 ± 0.98 ) |  | 0.06 | 0.91 ( 0.82 ± 1.01 ) |
|  | 22q12.2 | | rs5753037 | e | T | C | 0.63 | 1.02 ( 0.94 ± 1.11 ) |  | 0.09 | 1.09 ( 0.99 ± 1.19 ) |  | 0.08 | 1.1 ( 0.99 ± 1.22 ) |
|  | 22q12.2 | | rs713875 | e | C | G | 0.77 | 1.01 ( 0.93 ± 1.1 ) |  | **0.02** | 0.9 ( 0.81 ± 0.98 ) |  | 0.07 | 0.91 ( 0.82 ± 1.01 ) |
|  | 22q12.3 | *IL2RB* | rs3218253 | e | A | G | 0.77 | 0.99 ( 0.89 ± 1.09 ) |  | 0.61 | 1.03 ( 0.92 ± 1.16 ) |  | 0.78 | 1.02 ( 0.89 ± 1.16 ) |
|  | 22q13.1 | *IL2RB* | rs229527 | e | A | C | 0.19 | 1.05 ( 0.97 ± 1.14 ) |  | 0.24 | 1.06 ( 0.96 ± 1.16 ) |  | 0.16 | 1.07 ( 0.97 ± 1.19 ) |
|  | 22q13.1 | *IL2RB* | rs229541 | e | A | G | 0.23 | 1.05 ( 0.97 ± 1.14 ) |  | 0.21 | 1.06 ( 0.97 ± 1.16 ) |  | 0.15 | 1.08 ( 0.97 ± 1.19 ) |
| The Table lists GENIE GWAS association results (three studied GENIE phenotypes: DN, “ESRD vs. non-ESRD” and “ESRD vs. normoalbuminuria”) for SNPs that have been previously associated with T1D or chronic kidney disease. All analyses followed the GWAS analysis methods and were adjusted for T1D duration, age, sex, first ten principal components and study site (for UK-ROI and GoKinD US studies). See statistical analysis method section for full details. CKD = chronic kidney disease; eGFRcrea = estimted glomerular filtration rate based on serum cheatinine; eGFRcys = estimated glomerular filtration rate based on serum cystatin level; Gene = the suggested causal gene/genes; A1 = reference allele; A2 = non-reference allele; OR = odds ratio for minor allele; 95% CI = 95% confidence interval; REF = SNP reference: ^a^From Köttgen *et al*. 2009[1]; ^b^From Köttgen *et al.* 2010[2]; ^c^From Chambers *et al*. 2010 [3]; ^d^From Böger *et al.* 2011 [4]; ^e^From T1DBase (http://t1dbase.org)[5] | | | | | | | | | | | | | | |

Reference List

1. Kottgen A, Glazer NL, Dehghan A, Hwang SJ, Katz R, *et al.* (2009) Multiple loci associated with indices of renal function and chronic kidney disease. Nat Genet 41: 712-717.

2. Kottgen A, Pattaro C, Boger CA, Fuchsberger C, Olden M, *et al.* (2010) New loci associated with kidney function and chronic kidney disease. Nat Genet 42: 376-384.

3. Chambers JC, Zhang W, Lord GM, van der HP, Lawlor DA, *et al.* (2010) Genetic loci influencing kidney function and chronic kidney disease. Nat Genet 42: 373-375.

4. Boger CA, Chen MH, Tin A, Olden M, Kottgen A, *et al.* (2011) CUBN is a gene locus for albuminuria. J Am Soc Nephrol 22: 555-570.

5. Burren OS, Adlem EC, Achuthan P, Christensen M, Coulson RM, Todd JA (2011) T1DBase: update 2011, organization and presentation of large-scale data sets for type 1 diabetes research. Nucleic Acids Res 39: D997-1001.
