# Supplementary material for: New Susceptibility Loci Associated with Kidney Disease in Type 1 Diabetes
Source: PLoS Genet. 2012 Sep 20;8(9):e1002921. doi: 10.1371/journal.pgen.1002921 (PMC3447939; doi:10.1371/journal.pgen.1002921)
Supplement: Table S10 — Gene set enrichment analysis with MAGENTA. Gene sets with nominal P-value<0.01 for the three analyzed phenotypes. (DOC) [file pgen.1002921.s014.doc]

**Table S10. Gene set enrichment analysis with MAGENTA**. Gene sets with nominal *P*-value <0.01 for the three analyzed phenotypes.

| **DN phenotype (proteinuria + ESRD vs. normoalbuminuria)** | | | |  |  |  |
| --- | --- | --- | --- | --- | --- | --- |
|  | Data base | Gene set | P-value | Expected | Observed | Flagged genes |
|  | GO term | sugar binding | 6.00E-04 | 7 | 16 |  |
|  | GO term | double-stranded DNA binding | 1.30E-03 | 3 | 10 | *HDAC9* |
|  | Panther | Oogenesis | 1.70E-03 | 3 | 9 |  |
|  | GO term | ligase activity | 2.50E-03 | 14 | 25 | *PAK7* |
|  | GO term | cell redox homeostasis | 2.60E-03 | 3 | 9 |  |
|  | GO term | histone deacetylase binding | 3.00E-03 | 2 | 6 |  |
|  | GO term | estrogen receptor binding | 3.10E-03 | 1 | 4 |  |
|  | GO term | retrograde vesicle-mediated transport, Golgi to ER | 3.10E-03 | 1 | 5 |  |
|  | Panther | Nucleic_acid_binding | 4.00E-03 | 29 | 43 |  |
|  | Panther | Damaged_DNA-binding_protein | 5.30E-03 | 1 | 5 |  |
|  | Panther | Non-receptor_tyrosine_protein_kinase | 6.10E-03 | 2 | 6 |  |
|  | GO term | positive regulation of mitosis | 8.50E-03 | 1 | 4 |  |
|  | GO term | postsynaptic density | 8.50E-03 | 4 | 9 |  |
|  | Panther | Cell_adhesion_molecule | 9.60E-03 | 4 | 10 |  |
|  | Panther | Cytoskeletal_regulation_by_Rho_GTPase | 9.90E-03 | 1 | 5 |  |
|  |  |  |  |  |  |  |
| **ESRD vs. non-ESRD** | | |  |  |  |  |
|  | Data base | Gene set | P-value | Expected | Observed | Flagged genes |
|  | GO term | metanephros development | 1.80E-03 | 1 | 5 |  |
|  | Panther | DNA_replication | 2.40E-03 | 1 | 4 | *TOP1MT* |
|  | Panther | Developmental_processes | 2.70E-03 | 21 | 34 | *AES, A2BP1* |
|  | GO term | sequence-specific DNA binding | 2.90E-03 | 20 | 33 | *CREBL2, VSX1, MKX* |
|  | GO term | positive regulation of transcription | 2.90E-03 | 6 | 14 | *RNF10* |
|  | GO term | cytokine receptor activity | 3.20E-03 | 1 | 4 |  |
|  | GO term | embryonic skeletal system development | 3.40E-03 | 1 | 5 |  |
|  | Panther | Homeobox_transcription_factor | 3.70E-03 | 8 | 16 | *VSX1, MKX* |
|  | KEGG | KEGG_NATURAL_KILLER_CELL_MEDIATED_CYTOTOXICITY | 4.30E-03 | 5 | 12 |  |
|  | GO term | oxygen binding | 4.30E-03 | 2 | 6 |  |
|  | GO term | translational initiation | 4.40E-03 | 2 | 6 | *EIF3F* |
|  | GO term | ion transmembrane transporter activity | 4.60E-03 | 1 | 4 |  |
|  | GO term | biological_process | 6.00E-03 | 25 | 37 | *GLI4* |
|  | Panther | Segment_specification | 6.10E-03 | 3 | 9 |  |
|  | GO term | ribonuclease P activity | 6.40E-03 | 0 | 3 |  |
|  | GO term | positive regulation of gene-specific transcription from RNA polymerase II promoter | 6.60E-03 | 3 | 8 |  |
|  | GO term | chromatin silencing | 7.00E-03 | 0 | 3 |  |
|  | GO term | positive regulation of interferon-gamma biosynthetic process | 8.20E-03 | 0 | 3 |  |
|  | GO term | cartilage development | 8.90E-03 | 2 | 6 |  |
|  | GO term | protein amino acid O-linked glycosylation | 9.70E-03 | 1 | 4 |  |
|  |  |  |  |  |  |  |
| **ESRD vs normoalbuminuria** | | |  |  |  |  |
|  | Data base | Gene set | P-value | Expected | Observed | Flagged genes |
|  | KEGG | KEGG_CIRCADIAN_RHYTHM_MAMMAL | 3.00E-03 | 1 | 4 |  |
|  | GO term | extrinsic to membrane | 3.00E-03 | 2 | 6 | *FRMD3* |
|  | Panther | Neurotransmitter_release | 3.90E-03 | 4 | 11 | *RIMS2* |
|  | GO term | negative regulation of smooth muscle cell proliferation | 4.10E-03 | 1 | 4 |  |
|  | Ingenuity | Natural.Killer.Cell.Signaling | 4.20E-03 | 1 | 5 |  |
|  | KEGG | KEGG_SPLICEOSOME | 5.20E-03 | 6 | 13 | *SFRS9* |
|  | GO term | microtubule cytoskeleton | 5.60E-03 | 2 | 6 |  |
|  | KEGG | KEGG_MATURITY_ONSET_DIABETES_OF_THE_YOUNG | 6.10E-03 | 1 | 5 |  |
|  | Panther | Segment_specification | 7.00E-03 | 3 | 9 |  |
|  | Panther | Dopamine_receptor_mediated_signaling_pathway | 7.20E-03 | 1 | 5 | *FRMD3* |
|  | Panther | Regulated_exocytosis | 8.00E-03 | 2 | 7 | *RIMS2* |
|  | GO term | mitochondrial membrane | 8.10E-03 | 2 | 7 |  |
|  | KEGG | KEGG_TASTE_TRANSDUCTION | 9.10E-03 | 2 | 6 |  |
|  | GO term | regulation of growth | 9.60E-03 | 2 | 7 |  |
|  |  |  |  |  |  |  |
| MAGENTA pathway analysis was performed simultaneously with KEGG, Panther and Ingenuity pathways and GO terms, 2580 gene sets in total. Expected = Expected number of genes. Observed = Observed number of genes. Flagged genes = Genes in the pathways with *P*-value < 1x10^-4^ in GWAS. | | | | | | |
