# Supplementary material for: New Susceptibility Loci Associated with Kidney Disease in Type 1 Diabetes
Source: PLoS Genet. 2012 Sep 20;8(9):e1002921. doi: 10.1371/journal.pgen.1002921 (PMC3447939; doi:10.1371/journal.pgen.1002921)
Supplement: Table S11 — Number of patients included in the study. (DOC) [file pgen.1002921.s015.doc]

**Table S11. Number of patients included in the study.**

|  | **Before QC** | | | | | | | |  | **After QC** | | | | | | |
| --- | --- | --- | --- | --- | --- | --- | --- | --- | --- | --- | --- | --- | --- | --- | --- | --- |
|  | **normo** | **micro** | **macro** | **ESRD** | **unknown** | **Non-ESRD total** | **DN + ESRD total** | **total** |  | **normo** | **micro** | **macro** | **ESRD** | **Non-ESRD total** | **DN + ESRD total** | **total** |
| FinnDiane | 1721 | 516 | 733 | 682 | 0 | 2970 | 1415 | 3652 |  | 1591 | 460 | 674 | 645 | 2725 | 1319 | 3370 |
| UK-ROI ^a^ | 956 | 0 | 567 | 265 | 42 | 1523 | 872 | 1830 |  | 903 | 0 | 538 | 246 | 1441 | 823 | 1726 |
| US GoKinD | 889 | 0 | 306 | 577 | 20 | 1195 | 903 | 1792 |  | 821 | 0 | 266 | 508 | 1087 | 774 | 1595 |
| **Discovery total** | **3566** | **516** | **1606** | **1524** | **62** | **5688** | **3190** | **7274** |  | **3315** | **460** | **1478** | **1399** | **5253** | **2916** | **6691** |
|  |  |  |  |  |  |  |  |  |  |  |  |  |  |  |  |  |
| DCCT/EDIC ^b^ | 1172 | 0 | 132 | 0 | 0 | 0 | 132 | 1304 |  | 1172 |  | 132 |  | 0 | 132 | 1304 |
| Steno | 381 | 60 | 368 | 91 | 118 | 809 | 459 | 1018 |  | 368 | 58 | 350 | 84 | 776 | 434 | 860 |
| France | 408 | 198 | 283 | 74 | 0 | 889 | 357 | 963 |  | 391 | 192 | 277 | 71 | 860 | 348 | 931 |
| UK - Rep | 350 | 0 | 106 | 30 | 299 | 456 | 136 | 785 |  | 310 | 0 | 86 | 24 | 396 | 110 | 420 |
| Diabetes Registry Scania | 302 | 94 | 107 | 35 | 0 | 503 | 142 | 538 |  | 290 | 91 | 103 | 35 | 484 | 138 | 519 |
| Italy | 185 | 0 | 42 | 170 | 0 | 227 | 212 | 397 |  | 167 | 0 | 40 | 124 | 207 | 164 | 331 |
| Sweden ( Umea+Stockholm) | 194 | 94 | 40 | 19 | 0 | 328 | 59 | 347 |  | 183 | 92 | 39 | 17 | 314 | 56 | 331 |
| FinnDiane - Rep | 283 | 21 | 22 | 7 | 3 | 326 | 29 | 336 |  | 254 | 16 | 19 | 7 | 289 | 26 | 296 |
| Romania | 64 | 27 | 66 | 28 | 0 | 157 | 94 | 185 |  | 56 | 23 | 60 | 25 | 139 | 85 | 164 |
| **Stage 2 cohorts total** | **3339** | **494** | **1166** | **454** | **420** | **3695** | **1620** | **5873** |  | **3191** | **472** | **1106** | **387** | **3465** | **1493** | **5156** |
|  |  |  |  |  |  |  |  |  |  |  |  |  |  |  |  |  |
| **Discovery + Stage 2** | **6905** | **1010** | **2772** | **1978** | **482** | **9383** | **4810** | **13147** |  | **6506** | **932** | **2584** | **1786** | **8718** | **4409** | **11847** |
| The number of samples included in the study before and after quality control (QC). DN-phenotype included the cases from the “DN + ESRD total” sub-group and controls from the normo group. ESRD (vs. non-ESRD) included cases from the ESRD group and controls from the “non-ESRD total” group. ESRD vs normo phenotype included the cases from ESRD group and controls from the normo group. Normo: normoalbuminuric subjects. Micro: microalbuminuria. Macro: macroalbuminuria, DN. Unknown: subjects with unclear nephropathy status, or unknown T1D duration, age at T1D onset, or sex. “Non-ESRD total”: patients in normo-, micro- and macroalbuminuria categories. “DN + ESRD total“: patients with macroalbuminuria or ESRD. ^A^ 39 DN cases in UK-ROI Cohort have either macroalbuminuria or ESRD. ^b^ Because the DCCT/EDIC cohort was analyzed as time-to-event analysis, dichotomization of participants into cases and controls is not appropriate; Numbers shown here indicate the number of events (development of severe nephropathy) and of censored controls who did not reach severe nephropathy during the follow-up. | | | | | | | | | | | | | | | | |
