# Supplementary material for: New Susceptibility Loci Associated with Kidney Disease in Type 1 Diabetes
Source: PLoS Genet. 2012 Sep 20;8(9):e1002921. doi: 10.1371/journal.pgen.1002921 (PMC3447939; doi:10.1371/journal.pgen.1002921)
Supplement: Table S12 — Quality control and filtering for the discovery GWAS data. (DOC) [file pgen.1002921.s016.doc]

**Table S12.** **Quality control and filtering for the discovery GWAS data.**

|  | **US GoKinD** | | **UK-ROI** | | **FinnDiane** | |
| --- | --- | --- | --- | --- | --- | --- |
| **QC Step** | **Subjects** | **SNPs** | **Subjects** | **SNPs** | **Subjects** | **SNPs** |
| *Raw GWAS Data (no QC) | 1,792 | 364,292 | 1,830 | 975,120 | 3652 | 610,000 |
| Pre-QC Steps* | 162 | 734 | 2 | -- | 11 | 10,990 |
| Unsuccessful Genotyping (0 Call Rate) | -- | -- | -- | -- | 35 | -- |
| Filter on SNP Call Rate (>90%) | -- | 0 | -- | 296 | -- | 2,252 |
| Filter on SNP MAF (>1%) | -- | 25 | -- | 179,985 | -- | 44,617 |
| Filter on Subject Call Rate (>95%) | 0 | -- | 27 | -- | 2 | -- |
| Filter on Extreme Heterozygosity | 16 | -- | 14 | -- | 19 | -- |
| Filter on IBD/Cryptic Relatedness | 4 | -- | 22 | -- | 39 | -- |
| Clustering Approaches  (MDS, PCA, Neighbor) | 15 | -- | 39 | -- | 0 | -- |
| HWE Filter (P < 1e-7) | -- | 234 | -- | 1,417 | -- | 185 |
| Missing by Haplotype (P < 1e-7) | -- | 2,200 | -- | 1,421 | -- | 2,100 |
| Missing by Phenotype (P < 1e-7) | -- | 0 | -- | 282 | -- | 237 |
| Test for Plate Effects | -- | 200 | -- | 32 | -- | 89 |
| *Final QC GWAS | 1,595 | 360,899 | 1,726 | 791,687 | 3,546 | 549,530 |

First row denotes raw GWAS data requiring QC and last row final counts after all QC steps. All other rows correspond to a specific QC step and number of subjects and or SNPs removed at that step.
