# Supplementary material for: New Susceptibility Loci Associated with Kidney Disease in Type 1 Diabetes
Source: PLoS Genet. 2012 Sep 20;8(9):e1002921. doi: 10.1371/journal.pgen.1002921 (PMC3447939; doi:10.1371/journal.pgen.1002921)
Supplement: Table S13 — Physicians and nurses participating in the collection of the FinnDiane study subjects. (DOC) [file pgen.1002921.s017.doc]

**Table S13. Physicians and nurses participating in the collection of the FinnDiane study subjects**

| **FinnDiane Study Centers** | **Physicians and nurses** |
| --- | --- |
| Anjalankoski Health Centre | S. Koivula, T. Uggeldahl |
| Central Finland Central Hospital, Jyväskylä | T. Forslund, A. Halonen, A. Koistinen, P. Koskiaho, M. Laukkanen, J. Saltevo, M. Tiihonen |
| Central Hospital of Åland Islands, Mariehamn | M. Forsen, H. Granlund, A-C. Jonsson, B. Nyroos |
| Central Hospital of Kanta-Häme, Hämeenlinna | P. Kinnunen, A. Orvola, T. Salonen, A. Vähänen |
| Central Hospital of Länsi-Pohja, Kemi | H. Laukkanen, P. Nyländen, A. Sademies |
| Central Ostrabothnian Hospital District, Kokkola | S. Anderson, B. Asplund, U. Byskata, P. Liedes, M. Kuusela, T. Virkkala |
| City of Espoo Health Centre |  |
| Espoonlahti | A. Nikkola, E. Ritola |
| Tapiola | M. Niska, H. Saarinen |
| Samaria | E. Oukko-Ruponen, T. Virtanen |
| Viherlaakso | A. Lyytinen |
| City of Helsinki Health Centre |  |
| Puistola | H. Kari, T. Simonen |
| Suutarila | A. Kaprio, J. Kärkkäinen, B. Rantaeskola |
| Töölö | P. Kääriäinen, J. Haaga, A-L. Pietiläinen |
| City of Hyvinkää Health Centre | S. Klemetti, T. Nyandoto, E. Rontu, S. Satuli-Autere |
| City of Vantaa Health Centre |  |
| Korso | R. Toivonen, H. Virtanen |
| Länsimäki | R. Ahonen, M. Ivaska-Suomela, A. Jauhiainen |
| Martinlaakso | M. Laine, T. Pellonpää, R. Puranen |
| Myyrmäki | A. Airas, J. Laakso, K. Rautavaara |
| Rekola | M. Erola, E. Jatkola |
| Tikkurila | R. Lönnblad, A. Malm, J. Mäkelä, E. Rautamo |
| Heinola Health Centre | P. Hentunen, J. Lagerstam |
| Helsinki University Central Hospital, Department of Medicine, Division of Nephrology | A. Ahola, M. Feodoroff, D. Gordin, O. Heikkilä, K Hietala, J. Kytö, S. Lindh, K. Pettersson-Fernholm, A. Sandelin, A-R Salonen, L. Salovaara, L. Thorn, J. Tuomikangas, T. Vesisenaho, J. Wadén |
| Herttoniemi Hospital, Helsinki | V. Sipilä |
| Hospital of Lounais-Häme, Forssa | T. Kalliomäki, J. Koskelainen, R. Nikkanen, N. Savolainen, H. Sulonen, E. Valtonen |
| Iisalmi Hospital | E. Toivanen |
| Jokilaakso Hospital, Jämsä | A. Parta, I. Pirttiniemi |
| Jorvi Hospital, Helsinki University Central Hospital | S. Aranko, S. Ervasti, R. Kauppinen-Mäkelin, A. Kuusisto, T. Leppälä, K. Nikkilä, L. Pekkonen |
| Jyväskylä Health Centre, Kyllö | K. Nuorva, M. Tiihonen |
| Kainuu Central Hospital, Kajaani | S. Jokelainen, P. Kemppainen, A-M. Mankinen, M. Sankari |
| Kerava Health Centre | H. Stuckey, P. Suominen |
| Kirkkonummi Health Centre | A. Lappalainen, M. Liimatainen, J. Santaholma |
| Kivelä Hospital, Helsinki | A. Aimolahti, E. Huovinen |
| Koskela Hospital, Helsinki | V. Ilkka, M. Lehtimäki |
| Kotka Heath Centre | E. Pälikkö-Kontinen, A. Vanhanen |
| Kouvola Health Centre | E. Koskinen, T. Siitonen |
| Kuopio University Hospital | E. Huttunen, R. Ikäheimo, P. Karhapää, P. Kekäläinen, M. Laakso, T. Lakka, E. Lampainen, L. Moilanen, L. Niskanen, U. Tuovinen, I. Vauhkonen, E. Voutilainen |
| Kuusamo Health Centre | T. Kääriäinen, E. Isopoussu |
| Kuusankoski Hospital | E. Kilkki, I. Koskinen, L. Riihelä |
| Laakso Hospital, Helsinki | T. Meriläinen, P. Poukka, R. Savolainen, N. Uhlenius |
| Lahti City Hospital | A. Mäkelä, M. Tanner |
| Lapland Central Hospital, Rovaniemi | L. Hyvärinen, S. Severinkangas, T. Tulokas |
| Lappeenranta Health Centre | P. Linkola, I. Pulli |
| Lohja Hospital | T. Granlund, M. Saari, T. Salonen |
| Loimaa Health Centre | A. Mäkelä, P. Eloranta |
| Länsi-Uusimaa Hospital, Tammisaari | I-M. Jousmaa, J. Rinne |
| Malmi Hospital, Helsinki | H. Lanki, S. Moilanen, M. Tilly-Kiesi |
| Mikkeli Central Hospital | A. Gynther, R. Manninen, P. Nironen, M. Salminen, T. Vänttinen |
| Mänttä Regional Hospital | I. Pirttiniemi, A-M. Hänninen |
| North Karelian Hospital, Joensuu | U-M. Henttula, P. Kekäläinen, M. Pietarinen, A. Rissanen, M. Voutilainen |
| Nurmijärvi Health Centre | A. Burgos, K. Urtamo |
| Oulankangas Hospital, Oulainen | E. Jokelainen, P-L. Jylkkä, E. Kaarlela, J. Vuolaspuro |
| Oulu Health Centre | L. Hiltunen, R. Häkkinen, S. Keinänen-Kiukaanniemi |
| Oulu University Hospital | R. Ikäheimo |
| Päijät-Häme Central Hospital | H. Haapamäki, A. Helanterä, S. Hämäläinen, V. Ilvesmäki, H. Miettinen |
| Palokka Health Centre | P. Sopanen, L. Welling |
| Pieksämäki Hospital | V. Javtsenko, M. Tamminen |
| Pietarsaari Hospital | M-L. Holmbäck, B. Isomaa, L. Sarelin |
| Pori City Hospital | P. Ahonen, P. Merensalo, K. Sävelä |
| Porvoo Hospital | M. Kallio, B. Rask, S. Rämö |
| Raahe Hospital | A. Holma, M. Honkala, A. Tuomivaara, R. Vainionpää |
| Rauma Hospital | K. Laine, K. Saarinen, T. Salminen |
| Riihimäki Hospital | P. Aalto, E. Immonen, L. Juurinen |
| Salo Hospital | A. Alanko, J. Lapinleimu, P. Rautio, M. Virtanen |
| Satakunta Central Hospital, Pori | M. Asola, M. Juhola, P. Kunelius, M-L. Lahdenmäki, P. Pääkkönen, M. Rautavirta |
| Savonlinna Central Hospital | E. Korpi-Hyövälti, T. Latvala, E. Leijala |
| South Karelia Central Hospital, Lappeenranta | T. Ensala, E. Hussi, R. Härkönen, U. Nyholm, J. Toivanen |
| Tampere Health Centre | A. Vaden, P. Alarotu, E. Kujansuu, H. Kirkkopelto-Jokinen, M. Helin, S. Gummerus, L. Calonius, T. Niskanen, T. Kaitala, T. Vatanen |
| Tampere University Hospital | I. Ala-Houhala, T. Kuningas, P. Lampinen, M. Määttä, H. Oksala, T. Oksanen, K. Salonen, H. Tauriainen, S. Tulokas |
| Tiirismaa Health Centre, Hollola | T. Kivelä, L, Petlin, L. Savolainen |
| Turku Health Centre | I. Hämäläinen, H. Virtamo, M. Vähätalo |
| Turku University Central Hospital | K. Breitholz, R. Eskola, K. Metsärinne, U. Pietilä, P. Saarinen, R. Tuominen, S. Äyräpää |
| Vaajakoski Health Centre | K. Mäkinen, P. Sopanen |
| Valkeakoski Regional Hospital | S. Ojanen, E. Valtonen, H. Ylönen, M. Rautiainen, T. Immonen |
| Vammala Regional Hospital | I. Isomäki, R. Kroneld, M. Tapiolinna-Mäkelä |
| Vaasa Central Hospital | S. Bergkulla, U. Hautamäki, V-A. Myllyniemi, I. Rusk |
