# Supplementary material for: New Susceptibility Loci Associated with Kidney Disease in Type 1 Diabetes
Source: PLoS Genet. 2012 Sep 20;8(9):e1002921. doi: 10.1371/journal.pgen.1002921 (PMC3447939; doi:10.1371/journal.pgen.1002921)
Supplement: Table S14 — The DCCT/EDIC Study Research Group. (DOC) [file pgen.1002921.s018.doc]

**Table S14. The DCCT/EDIC Study Research Group**

| **The following persons and institutions participated in the DCCT/EDIC Study Research Group:** | |
| --- | --- |
| Study Chairmen | S. Genuth, D.M. Nathan, B. Zinman (vice-chair), O. Crofford (past) |
| Albert Einstein College of Medicine | J. Crandall, M. Reid, J. Brown-Friday, S. Engel, J. Sheindlin, H. Martinez (past), H. Shamoon (past), H. Engel (past), M. Phillips |
| Case Western Reserve University | R. Gubitosi-Klug, L. Mayer, S. Pendegast, H. Zegarra, D. Miller, L. Singerman, S. Smith-Brewer, M. Novak, J. Quin (past), W. Dahms (deceased), Saul Genuth (past), M. Palmert (past) |
| Cornell University Medical Center | D. Brillon, M.E. Lackaye, S. Kiss, R. Chan, V. Reppucci (past), T. Lee (past), M. Heinemann (past) |
| Henry Ford Health System | F. Whitehouse, D. Kruger, J. K. Jones, M. McLellan (past), J.D. Carey, E. Angus, A. Thomas, A. Galprin (past) |
| International Diabetes Center | R. Bergenstal, M. Johnson, M. Spencer (past), K. Morgan, D. Etzwiler (deceased), D. Kendall (past) |
| Joslin Diabetes Center | Lloyd Paul Aiello, E. Golden, A. Jacobson (past), R. Beaser, O. Ganda, O. Hamdy, H. Wolpert, G. Sharuk, P. Arrigg, D. Schlossman, J. Rosenzwieg (past), L. Rand (past) |
| Massachusetts General Hospital | D.M. Nathan, M. Larkin, M. Ong, J. Godine, E. Cagliero, P. Lou, K. Folino, S. Fritz (past), S. Crowell (past), K. Hansen (past), C. Gauthier-Kelly (past) |
| Mayo Foundation | J. Service, G. Ziegler |
| Medical University of South Carolina | L. Luttrell, S. Caulder, M. Lopes-Virella (past), J. Colwell (past), J. Soule (past), J. Fernandes, K. Hermayer, S. Kwon, M. Brabham (past), A. Blevins, J. Parker, D. Lee (past), N. Patel, C. Pittman, P. Lindsey (past), M. Bracey (past), K. Lee, M. Nutaitis, A. Farr (past), S. Elsing (past), T. Thompson (past), J. Selby (past), T. Lyons (past), S. Yacoub-Wasef (past), M. Szpiech (past), D. Wood (past), R. Mayfield (past) |
| Northwestern University | M. Molitch, B. Schaefer, L. Jampol, A. Lyon, M. Gill, Z. Strugula, L. Kaminski, R. Mirza, E. Simjanoski, D. Ryan |
| University of California, San Diego | O. Kolterman, G. Lorenzi, M. Goldbaum |
| University of Iowa | W. Sivitz, M. Bayless |
| University of Maryland School of Medicine | D. Counts, S. Johnsonbaugh, M. Hebdon (past), P. Salemi, R. Liss, T. Donner (past), J. Gordon (past), R. Hemady (past), A. Kowarski (past), D. Ostrowski (past) S. Steidl (past), B. Jones (past) |
| University of Michigan | W.H. Herman, C.L. Martin, R. Pop-Busui, A. Sarma, J. Albers, E. Feldman, K. Kim, S. Elner, G. Comer, T. Gardner, R. Hackel, R. Prusak, L. Goings, A. Smith, J. Gothrup, P. Titus, J. Lee, M. Brandle, L. Prosser, D.A. Greene (past), M.J. Stevens (past), A. K. Vine (past) |
| University of Minnesota | J. Bantle, N. Wimmergren, A. Cochrane, T. Olsen (past), E. Steuer (past), P Rath (past), B. Rogness (past) |
| University of Missouri | D. Hainsworth, D. Goldstein , S. Hitt, J. Giangiacomo |
| University of New Mexico | D.S. Schade, J.L. Canady, J.E. Chapin, L.H. Ketai C |
| University of Pennsylvania | S. Braunstein, P.A. Bourne, S. Schwartz (past), A. Brucker , B.J. Maschak-Carey (past), L. Baker (deceased) |
| University of Pittsburgh | T. Orchard, N. Silvers, C. Ryan, T. Songer, B. Doft, S. Olson, R.L. Bergren, L. Lobes, P. Paczan Rath, D. Becker, D. Rubinstein, P.W. Conrad, S. Yalamanchi, A. Drash (past) |
| University of South Florida | A. Morrison, M.L. Bernal, J. Vaccaro-Kish (past), J. Malone, P.R. Pavan, N. Grove, M.N. Iyer, A.F. Burrows, E.A. Tanaka (past), R. Gstalder (past) |
| University of Tennessee | S. Dagogo-Jack, C. Wigley, H. Ricks, A. Kitabchi, M. B. Murphy (past), S. Moser (past), D. Meyer (past), A. Iannacone (past), E. Chaum, S. Yoser (past), M. Bryer-Ash (past), S. Schussler (past), H. Lambeth (past) |
| The University of Texas Southwestern Medical Center at Dallas | P. Raskin, S. Strowig |
| University of Toronto | B. Zinman, A. Barnie, R. Devenyi, M. Mandelcorn, M. Brent, S. Rogers, A. Gordon |
| University of Washington | J. Palmer, S. Catton, J. Brunzell, H. Wessells, I. H. de Boer, J. Hokanson, J. Purnell, J. Ginsberg, J. Kinyoun, S. Deeb, M. Weiss, G. Meekins, J. Distad, L. Van Ottingham (past) |
| University of Western Ontario | J. Dupre, J. Harth, D. Nicolle, M. Driscoll, J. Mahon, C. Canny |
| Vanderbilt University | M. May, J. Lipps, A. Agarwal, T. Adkins, L. Survant, R. L. Pate, G. E. Munn, R. Lorenz (past), S. Feman (past) |
| Washington University, St. Louis | N. White, L. Levandoski, I. Boniuk, G. Grand, M. Thomas, D. D. Joseph, K. Blinder, G. Shah, Boniuk (past), Burgess (past), J. Santiago (deceased) |
| Yale University School of Medicine | W. Tamborlane, P. Gatcomb, K. Stoessel, K. Taylor (past)J. Goldstein (past), S. Novella (past), H. Mojibian (past), D. Cornfeld (past) |
| Clinical Coordinating Center (Case Western Reserve University) | R. Gubitosi-Klug, J. Quin, P. Gaston, M. Palmert (past), R. Trail (past), W. Dahms (deceased) |
| Data Coordinating Center (The George Washington University, The Biostatistics Center) | J. Lachin, P. Cleary, J. Backlund, W. Sun, B. Braffett, K. Klumpp, K. Chan (past), L. Diminick, D. Rosenberg (past), B. Petty (past), A. Determan (past), D. Kenny (past), B. Rutledge (past), Naji Younes (past), Williams (past), L. Dews, M. Hawkins |
| National Institute of Diabetes and Digestive and Kidney Disease Program Office | C. Cowie, J. Fradkin, C. Siebert (past), R. Eastman (past) |
| Central Fundus Photograph Reading Center (University of Wisconsin) | R. Danis, S. Gangaputra, S. Neill, M. Davis (past), L. Hubbard (past), H. Wabers, M. Burger, J. Dingledine, V. Gama, R. Sussman |
| Central Biochemistry Laboratory (University of Minnesota) | M. Steffes, J. Bucksa, M. Nowicki, B. Chavers |
| Central Carotid Ultrasound Unit (New England Medical Center) | D. O’Leary, J. Polak, A. Harrington, L. Funk (past) |
| Central ECG Reading Unit (University of Minnesota) | R. Crow (past), B. Gloeb (past), S. Thomas (past), C. O’Donnell (past) |
| Central ECG Reading Unit (Wake Forest University) | E. Soliman, Z.M. Zhang, R. Prineas (past), C. Campbell |
| Central Neuropsychological Coding Unit | C. Ryan, D. Sandstrom, T. Williams, M. Geckle, E. Cupelli, F. Thoma, B. Burzuk, T. Woodfill |
| Central ANS Reading Unit (Mayo Clinic) | P. Low, C. Sommer, K. Nickander |
| Computed Tomography Reading Center (Harbor UCLA Research and Education Institute) | M. Budoff, R. Detrano (past), N. Wong, M. Fox, L. Kim (past), R. Oudiz |
| Johns Hopkins Medical Institutions | J. Lima, D. Bluemke, E. Turkbey, R. J. van der Geest, C. Liu, A. Malayeri, A. Jain, C. Miao (past), H. Chahal (past), R. Jarboe (past) |
| External Evaluation Committee | G. Weir (Chairman), M. Espeland, B. Klein, T Manolio, L. Rand, D. Singer, M. Stern, A.E. Boulton, C. Clark (past), R. D’Agostino (past) |
| Molecular Risk Factors Program Project (Medical University of South Carolina) | M. Lopes-Virella, W.T. Garvey (past), T.J. Lyons, A. Jenkins, R. Klein, G. Virella, A. Jaffa, Rickey Carter, D. Lackland (past), M. Brabham (past), D. McGee (past), D. Zheng (past), R. K. Mayfield (past) |
| Genetic Studies Group (Hospital for Sick Children) | A. Paterson, A. Boright, S. Bull, L. Sun, S. Scherer (past), B. Zinman (past) |
| SCOUT (Veralight) | J. Maynard |
| Epigenetics (Beckman Research Institute of City of Hope Medical Center) | R. Natarajan, F. Miao, L. Zhang, Z. Chen |
| Editor, EDIC Publications | D.M. Nathan |
