# Supplementary material for: New Susceptibility Loci Associated with Kidney Disease in Type 1 Diabetes
Source: PLoS Genet. 2012 Sep 20;8(9):e1002921. doi: 10.1371/journal.pgen.1002921 (PMC3447939; doi:10.1371/journal.pgen.1002921)
Supplement: Text S1 — Supplementary Methods: Detailed explanation of employed methods. (DOC) [file pgen.1002921.s019.doc]

**Supplementary Methods**

**New susceptibility loci associated with kidney disease in type 1 diabetes**

Niina Sandholm, Rany M Salem, Amy Jayne McKnight, Eoin P Brennan, Carol Forsblom, Tamara Isakova, Gareth J McKay, Winfred W Williams, Denise M Sadlier, Ville-Petteri Mäkinen, Elizabeth J Swan, Cameron Palmer, Andrew P Boright, Emma Ahlqvist, Harshal A Deshmukh, Benjamin J Keller, Huateng Huang, Aila J Ahola, Emma Fagerholm, Daniel Gordin, Valma Harjutsalo, Bing He, Outi Heikkilä, Kustaa Hietala, Janne Kytö, Päivi Lahermo, Markku Lehto, Anne-May Österholm, Maija Parkkonen, Janne Pitkäniemi, Milla Rosengård-Bärlund, Markku Saraheimo, Cinzia Sarti, Jenny Söderlund, Aino Soro-Paavonen, Anna Syreeni, Lena M Thorn, Heikki Tikkanen, Nina Tolonen, Karl Tryggvason, Jaakko Tuomilehto, Johan Wadén, Geoffrey V Gill, Sarah Prior, Candace Guiducci, Daniel B Mirel, Andrew Taylor, S Mohsen Hosseini, DCCT/EDIC Research Group, Hans-Henrik Parving, Peter Rossing, Lise Tarnow, Claes Ladenvall, François Alhenc-Gelas, Pierre Lefebvre, Vincent Rigalleau, Ronan Roussel, David-Alexandre Tregouet, Anna Maestroni, Silvia Maestroni, Henrik Falhammar, Tianwei Gu, Anna Möllsten, Danut Cimponeriu, Mihai Ioana, Maria Mota, Eugen Mota, Cristian Serafinceanu, Monica Stavarachi, Robert L Hanson, Robert G Nelson, Matthias Kretzler, Helen M Colhoun, Nicolae Mircea Panduru, Harvest F Gu, Kerstin Brismar, Gianpaolo Zerbini, Samy Hadjadj, Michel Marre, Leif Groop, Maria Lajer, Shelley B Bull, Daryl Waggott, Andrew D Paterson, David A Savage, Stephen C Bain, Finian Martin, Joel N Hirschhorn, Catherine Godson, Jose C Florez, Per-Henrik Groop, and Alexander P Maxwell

**Online Supplementary Methods**

## Study Populations

We implemented a two stage analysis, in which a genome-wide association study (GWAS) was performed using a set of three discovery cohorts, and 24 top signals for diabetic nephropathy (DN) and end-stage renal disease (ESRD) analysis were analyzed further in the second phase in an additional set of nine independent cohorts as described in the statistical analysis section below, to minimize false positives.

## Discovery Cohorts

DNA collections employed in the discovery phase were specifically designed to study DN and have previously identified genetic association with this disease [1–4]. For the discovery analysis we evaluated a total of 3,223 DN cases and 3,620 controls from three independent studies in the GEnetics of Nephropathy: an International Effort (GENIE) consortium: UK-ROI, FinnDiane, and GoKinD US. Ethical approval was obtained for each study center, and written informed consent was provided by all study participants.

**Collections genotyped in phase 2**

DNA was sought from worldwide case-control collections of individuals with type 1 diabetes (T1D) and known renal status. A total of 5,741 individuals from nine independent collections were genotyped for the top-ranked SNPs (n=41 including 17 proxies), with the exception of the DCCT/EDIC cohort where data was imputed. All the patients included in the phase two analysis were adults of European descent who had T1D diagnosed before 35 years of age. Controls with normal albumin excretion rate (AER) had duration of T1D at least 15 years, and cases with DN had minimum T1D duration of 10 years. If a collection included patients with microalbuminuria, they were excluded from the primary analysis of DN, but included as controls in the analysis of ESRD versus non-ESRD. The main clinical characteristics of all the replication cohorts are shown in the Table S2.

**FinnDiane:** Patients in the FinnDiane phase two cohort were selected from the FinnDiane and National Institute of Health and Welfare T1D collections with the same diagnostic criteria as the FinnDiane discovery cohort. The patients in the replication collection did not overlap with the discovery set.

**UK:** All additional samples genotyped in the second phase met the same inclusion criteria as the discovery collection [5]. This group includes individuals recruited as part of the Golden Years collection [6], where all persons are recipients of the Alan Nabarro or RD Lawrence medals, which are awarded after 50 years and 60 years of insulin treatment respectively.

**DCCT/EDIC:** The DCCT was a multi-center randomized clinical trial to compare intensive and conventional insulin therapy on the development and progression of early vascular and neurological complications of T1D [7–11]. We studied 1,304 white probands (out of 1,441 DCCT subjects). Renal outcomes were defined as time in years from DCCT baseline until the event. AERs were measured annually in DCCT and every other year in EDIC. Renal outcomes included data up to EDIC year 12 (2005): which involved a follow-up of 17.5 ± 2.6 years (mean ± SD) in DCCT/EDIC with 12 ± 2 AER measures. Persistent microalbuminuria was defined as the time to two consecutive AER >30 mg/24 hours (>20.8 µg min^-1^); severe nephropathy was the time to AER >300mg/24 hours (>208 µg min^-1^) with prior persistent microalbuminuria, or ESRD. For persistent microalbuminuria, 22% developed the outcome during follow-up (268 events vs. 976 censored); while for severe nephropathy, 10% developed the outcome (132 events vs. 1172 censored). Cox proportional hazards analysis of discrete time-to-event outcomes was performed using two models with additive genotype coding. The first model was ‘univariate’ including DCCT cohort, treatment, cohort-treatment interaction as covariates, and stratified by the year of entry into DCCT. Our primary analysis was a multivariate Cox model including cohort, treatment, cohort-treatment, age of diagnosis, DCCT baseline duration, sex, BMI, mean blood pressure, triglyceride, HDL-cholesterol, total cholesterol, baseline smoking, glycated hemoglobin eligibility, time-dependent updated mean glycated hemoglobin, and time-dependent indicators for hypertension diagnosis and/or treatment of hypertension, with stratification by DCCT year of entry.

**Steno:** From 1993 to 2000, adult white patients with T1D and DN attending the outpatient clinic at Steno Diabetes Center have been invited to participate in a study of genetic risk factors for the development of diabetic complications [12]. Of these, 73% accepted. T1D was considered present if the age at onset of diabetes was ≤ 35 years and time to definite insulin therapy ≤ 1 year. In total, 458 patients with DN defined by persistent albuminuria (>300 mg/24 h) in two out of three consecutive measurements, presence of retinopathy, and absence of other kidney or urinary tract disease were enrolled as cases. Absence of DN (controls) was defined as persistent normoalbuminuria (<30mg/24 h) after more than 15 years of T1D in patients not treated with ACE inhibitors or angiotensin-II receptor blockers. In total, 442 were included as controls. All patients had blood samples and phenotypic characteristics collected as part of the EURAGEDIC project. From venous samples, glycated hemoglobin was measured by standard high-performance liquid chromatography (normal range: 4.1–6.4%) (Tosoh automated glycohemoglobin analyzer; Tosoh Bioscience, Minato, Japan). Urinary AER was measured in 24-h urine collections by an enzyme immunoassay. Serum creatinine concentration was determined by a modified Jaffe's method. Glomerular filtration rate (GFR) was measured annually in patients with DN after a single injection of 3.7 MBq ^51^Cr-EDTA by determination of radioactivity in venous blood samples taken 180, 200, 220, and 240 min after injection [13]. The results were standardized for 1.73 m^2^ body surface area, using the patient's surface area at the start of the study. The mean day-to-day coefficient of variation is 4% in our laboratory. In patients with normoalbuminuria, the GFR at baseline was estimated by the MDRD equation [14]. ESRD was defined as chronic dialysis or kidney transplantation. On the basis of standardized questionnaires, current smokers of one or more cigarettes/cigars/pipes per day were classified as smokers and all others as non-smokers.

**Scania Diabetes Registry:** The Scania Diabetes Registry (SDR) registers all individuals with diabetes in the Malmö region in Southern Sweden [15]. T1D was defined by age of onset (≤35 years), presence of GAD antibodies and low C-peptide levels or, in case of incomplete information, on the physician’s own classification. Patients of non-Scandinavian origin were excluded from the analysis. DN was classified as follows: normoalbuminuria (AER <20 μg min^-1^ or <30 mg/24 h, or albumin to creatinine ratio [ACR] <2.5 for men and 3.5 for women, duration of T1D >15 years; macroalbuminuria (AER ≥200 μg min^-1^ or ≥300 mg/24 h). End-stage renal disease was defined as estimated glomerular filtration rate (eGFR) <15 ml min^-1^, estimated by the MDRD4 formula. After genotype quality control [QC] there were 290 individuals with normoalbuminuria, 103 individuals with macroalbuminuria and 35 individuals with ESRD.

**France-Belgium: GENEDIAB & GENESIS Cohorts**

The GENEDIAB [16] and GENESIS [17] patients were recruited in France and Belgium, in 1994/1995 and 1999-2001, respectively. More than 800 patients with long-standing T1D were classified as nephropathic cases and controls. Selection criteria checking and renal disease staging was performed by an adjudication committee reviewing patients’ records and ophthalmological reports. In both cohorts, patients with T1D were selected on the following criteria: 1) age at diabetes onset before age 35 years, and 2) definitive insulin use within one year after diagnosis. DN was classified according to the highest 3 AER measurements, within the last 5 years, collected on sterile urines, and serum creatinine determined in a central laboratory. Categories included: 1) controls (normoalbuminuria), 2) incipient nephropathy (microalbuminuria), 3) established nephropathy (proteinuria), and 4) advanced nephropathy (serum creatinine > 150 μmol L^-1^ and/or renal replacement therapy). In the GENEDIAB cohort subjects were selected on the presence of microvascular disease evidenced by past or present severe retinopathy: either severe non-proliferative retinopathy requiring laser pan-photocoagulation or proliferative retinopathy. In the GENESIS cohort subjects with diabetic retinal involvement (background or proliferative) could be included.

**Italy cohort:** The Italian cohort comprises 356 patients affected by T1D, 188 with established DN and 168 with normal renal function despite a similar age and duration of diabetes. DN was defined as a median AER >200 µg min^-1^ in three overnight collections of sterile urine in patients with T1D for at least 10 years, concomitant diabetic retinopathy and absence of clinical or laboratory evidence of cardiac failure or other renal or urinary tract disease. Patients without nephropathy had a median AER <20 µg min^-1^. Italian individuals with T1D were unrelated, white subjects with onset of diabetes before 40 years of age collected at the Complications of Diabetes Unit of the San Raffaele Scientific Institute, Milan, Italy. The study was approved by the Ethics Committee of the San Raffaele Scientific Institute, Milan, Italy and informed consent was obtained from all participants.

**Sweden:** The Swedish cohort was collected from the Department of Endocrinology in Stockholm and the Department of Medicine in Umeå, Sweden. All patients with T1D were Swedish and diagnosed before 30 years of age. The patients with macroalbuminuria (urinary AER ≥ 200 µg min^-1^ in at least two consecutive overnight samples) were defined as cases (males/females 40/26; mean age 52/48 yrs; mean BMI 25.8/25.8 kg m^-2^; mean serum creatinine 1.98/1.59 mg dl^-1^; mean glycated hemoglobin 6.9/7.7%). The patients with AER < 20 µg min^-1^ were considered as controls (males/females 103/108; mean age 46/46 yrs; mean BMI 25.2/24.7 kg m^-2^; mean serum creatinine 0.80/0.66 mg dl^-1^; mean glycated hemoglobin 6.9/7.5%). Diabetes durations in cases were 34 and 32 yrs in males and females, while the durations in controls were 27 and 29 yrs respectively. The patients with microalbuminuria (AER 20-200 µg min^-1^) were included only as controls for the ESRD versus non-ESRD analysis in the present study.

**Romania:** RomDiane is a Romanian study, with two participating centers – Bucharest and Craiova. The aim of the study is to identify risk factors for DN and other chronic complications in patients with T1D in the Romanian population. The study, with a cross-sectional design, enrolled patients with T1D diagnosed before the age of 40 years and with insulin treatment started within 1 year from diagnosis. The data regarding diabetic complications, cardiovascular status, personal and family medical history, and medication use were assessed by a standardized questionnaire completed by the investigators. Disease status was defined based on urine AER or urine ACR in at least two out of three consecutive urine collections at local centers. Patients without DN were required to have more than 15 years of diabetes duration without microalbuminuria. ESRD was defined as ongoing dialysis or renal transplant.

## Genetic Analysis

**Genotyping:** Existing DNA from individuals in the UK-ROI collection were genotyped using the Omni1-Quad array (Illumina, San Diego, CA, USA) at the Broad Institute. For FinnDiane, DNA was extracted with the PureGene Gentra method (Gentra systems, Minneapolis, MN, USA). Phenol extraction method was used for some of the older samples to improve DNA yield. The FinnDiane samples were genotyped at the Institute of Molecular Medicine Finland (FIMM, Helsinki, Finland) on the Illumina's BeadArray 610 Quad array. Illumina's BeadStudio clustering algorithm was used to call genotypes in both the UK-ROI and FinnDiane. Existing genotype data for the GoKinD US genotype data was downloaded from dbGAP (phs000018.v2.p1, retrieved June 2010), containing genotype data from Affymetrix 500K set (Affymetrix, Santa Clara, CA, USA). Version 2 genotype data differed from the original US GoKinD data, containing updated and recalled genotype calls for a previously reported problematic plate [18] and additional QC steps performed by NHLBI.

**Data Cleanup / Quality Control:**

Samples for UK-ROI and FinnDiane were excluded for insufficient DNA quality, quantity or poor genotype concordance with previous genotypes during a fingerprint evaluation stage. In the UK-ROI sample, 1,830 unique case (n=872) and control (n=958) individuals were submitted for genotyping on the Omni1-quad. For FinnDiane, 3,651 individuals (cases, n=1,934; controls n=1,721) were submitted for genotyping on the 610 Quad. For all three discovery datasets (UK-ROI, FinnDiane, GoKinD US), uniform and extensive genotype quality control procedures were applied: SNPs were filtered for those with call rates greater than 90%, minor allele frequency (MAF) exceeding 1%, and concordance with Hardy Weinberg Equilibrium (HWE, *P*<10^-7^). Sample filters included individual call rates greater than 95%, no extreme heterozygosity and cryptic relatedness as determined using identity by descent (first-degree relatives, estimated identity by descent >0.4), and admixture assessment using principal components (plotted with HapMap reference panel, Figure S4). Additional quality control measures included test of missing by haplotype (*P*<10^-8^), missing by phenotype (*P*>10^-8^) and plate effects (*P*<10^-7^). These quality control steps were performed using PLINK [19] with custom perl and R analysis scripts. Known copy number variation and mitochondrial SNPs were excluded from analyses. Detailed results of each QC step are reported in Table S12 for each study population.

A HapMap control sample was included on all genotyping plates for UK-ROI; average call rate was 99.9% with HapMap concordance equaling 99.7%. The average sample call rate was 99.5% in UK-ROI with sample heterozygosity 22.1%. Concordance with internal control for FinnDiane was 99.996% with an average sample call rate of 99.8%.

In total, directly genotyped results for 823 cases and 903 controls in 791,687 SNPs passed QC procedure in UK-ROI. Similarly, 549,530 SNPs with average genotyping rate of 99.9% passed the QC filters in 1,319 cases, 1,591 controls and 460 individuals with microalbuminuria for FinnDiane. 360,899 SNPs in 774 cases and 821 controls for GoKinD US passed quality control and were included in the analysis.

**Population stratification:** Principal Component Analysis (PCA) was performed separately for each of the three studies with the EIGENSTRAT program [20] in order to detect genetic outliers and to adjust the analyses for population structure. Genetic outliers were defined as more than six standard deviations away from the center of distribution along any of the ten first principal components and the procedure was repeated until no outliers were detected. After filtering, PCA were calculated for each study cohort combined with unrelated individuals from three original HapMap populations ([www.hapmap.org](http://www.hapmap.org/)), and plotted to identify additional admixed individuals. The first ten principal components were employed to adjust the association analysis for any residual population structure from the cleaned datasets.

**SNP imputation:** After quality control, MACH 1.0 software (<http://www.sph.umich.edu/csg/abecasis/MACH>) was used to perform imputation using HapMap phase II CEU population as a reference, resulting in ~2.4 million SNPs for each cohort. The cross-over and error rates were estimated with 50 iteration rounds in roughly 300 randomly selected samples. The imputation was run with the greedy algorithm and the maximum likelihood method in order to obtain expected allele dosages rather than integer allele counts. SNPs with low imputation quality (r^2^ < 0.6) are not reported.

**Statistical analysis:** PLINK v1.07 [21] was employed to conduct association tests for the allele dosage data with logistic regression adjusted for sex, age, the duration of diabetes and the ten first components of the study specific principal component analysis. UK-ROI and GoKinD US were adjusted for study centre, but in the primary DN phenotype the two GoKinD US centers GWU and JDC were analyzed separately as performed in the original GoKinD US analyses [22]. For the ESRD outcome, samples were analyzed together adjusting for recruitment center due to uneven distribution of ESRD cases by site and modest number of ESRD cases. Results from individual studies were adjusted for study specific genomic inflation factor and then combined by fixed effect meta-analysis model using METAL [23], to estimate the combined effect sizes and significances from beta values and standard error. Significance results were extracted for related phenotypes (Tables S6 and S7). The power to detect a nominal association (alpha=0.05) between previously reported CKD/eGFR/T1DN SNPs was calculated using the genetics power calculator [24] for the DN and ESRD outcomes separately. The combined GENIE cohorts had 80% power to identify a DN-associated SNP with OR=1.08 and MAF=20% (alternatively, 80% power for SNP with MAF=10% and OR=1.14). Power was lower for the ESRD endpoint (n=1,138 cases), 80% power was achieved for 20% MAF SNP and OR=1.15 (and 80% power for SNP with MAF=10% and OR=1.18).

**Second phase SNP selection and design:** All SNPs observed with *P*<10^-5^ were selected for further analysis. These SNPs were reviewed and a top SNP (with a proxy) was selected for each independent signal using the LD-based clumping procedure implemented in PLINK (SNPs more than 500 kb distant or LD r^2^<0.3 in HapMap II CEU). SNPs were annotated with associated genes and function using dbSNP build 132, human build 37.1. Cytogenetic locations for genes were sourced from Entrez gene; locations for SNPs that were not associated with genes were recorded from NCBI MapView. Regional association plots were generated using hg18 in LocusZoom [25].

**Second phase genotyping and statistical analysis:** *de novo* genotyping for all phase two cohorts except for DCCT/EDIC was performed using identical designs of Sequenom IPLEX assays (Sequenom Inc, San Diego, US). The quality control of the genotypes included filtering for low sample genotyping rate (<0.9), low SNP genotyping rate (<0.95), low minor allele frequency (<0.01) and deviation from Hardy Weinberg equilibrium (*P*<10^-4^). Additionally SNPs with low minor allele count (MAC<10) within the corresponding case group were excluded in order to ensure the stability of the statistical analysis. The DCCT/EDIC provided results from their GWAS on time to DN, using genotyped and imputed SNPs, which had undergone their respective quality control procedures (see Table S2 for the details of the genotyping and the quality control in each cohort). The statistical analysis used same covariates as the discovery stage, with exception there was no adjusted for principal components. All results were then combined by meta-analysis using METAL software as previously described.

**Longitudinal analysis:** Longitudinal data from the FinnDiane discovery cohort was used to perform time-to-event analysis using Kaplan-Meier and Cox proportional hazards regression, to evaluate the genetic association of rs7583877 and rs12437854 with the duration from the onset of T1D until various end points: microalbuminuria, macroalbuminuria or ESRD. Additionally, we analyzed time from onset of macroalbuminuria to ESRD. The most recent kidney status data was utilized for each patient. The year of the complication onset (microalbuminuria, macroalbuminuria, ESRD) was determined from the FinnDiane study questionnaires filled by the attending physician, or from the participants’ medical records. All subjects that had not reached the studied endpoint by the time of their latest FinnDiane visit or update of their medical records, were included as controls, and censored out at the latest known duration. For example, the “time from T1D onset to macroalbuminuria” analysis included also patients with microalbuminuria as controls. The latest data for ESRD was obtained from the Finnish Hospital Discharge Registry (HILMO, as per 30.12.2009), and these data were available for all participants.

We also examined whether the two main association signals, rs7583877 and rs12437854, were associated with mortality using data from the Finnish Death Registry (as per 30.9.2010). As DN is strongly associated with mortality, the time until death was analyzed separately for participants without DN (time from T1D onset to death; participants who developed DN were censored out at the time of the onset of DN) and for those with DN (time from onset of DN to death and time from onset of ESRD to death). We also performed the analysis of time from ESRD onset to death including only participants with incident ESRD, in order to avoid selection bias: If the loci would affect ESRD specific mortality, then the set of cross-sectionally sampled ESRD-participants would be affected by survival bias. The number of cases and controls for each analysis is given in Table S6.

Before the statistical analysis, we converted the allele dosage data for both SNPs into the most likely genotypes as given by the MACH software used for imputation. Genotype probability of 0.9 was used as a cut-off for accepting the genotype calling. Statistical analyses were performed using ‘survival’ package in R software (version 2.36-10, <http://cran.r-project.org/web/packages/survival>).

**Gene set enrichment analysis:** Gene Ontology (GO) terms, KEGG and Ingenuity pathways and PANTHER database entries were included in the gene set enrichment analysis performed with MAGENTA [26]. The analysis assigns each gene the highest P-value within the gene region, and adjusts the results for gene size and marker density among other gene related variables. All the genes that were within 10 kb distance from a SNP with GWAS P < 10^-4^ were flagged in the results file. The 95 percentile cut-off was used for inclusion of genes in the gene sets.

**DN gene expression profiles:** Expression analysis was performed on human renal tissue obtained by protocol renal biopsies in early DN (NIDDK Pima Indian Cohort) as described by Berthier *et al.*[27]. Briefly, gene expression profiling from microdissected glomerular and tubular tissue were performed using the Human Genome U133A and plus2 Affymetrix Genechip arrays (Santa Clara, CA). The CEL files were normalized (Robust Multichip Average method) log2 transformed and batch-corrected using the Genepattern platform ([www.genepattern.com](http://www.genepattern.com/)). Differential gene expression was assessed by Significance Analysis of Microarrays (SAM) algorithm and significance was defined as a Q-value < 0.05 using healthy living donor kidney transplant biopsies as controls.

**Intra-renal genotype – transcript correlation:** 54 tubulointerstitial gene expression data sets and 65 glomerular expression data sets with matching Affymetrix 6.0 SNP profiles were available for eQTL analysis [28]. To identify allele specific differential regulation in a renal compartment specific manner for *cis* eQTL studies, the *cis* region of each gene was defined as 150 kb upstream of TSS and 50 kb downstream of TES.

Two SNPs (rs17418640, rs17418814) were found to have *cis* associations with intra-renal *ERBB4* expression. Causal analysis implicates rs17418640 as the primary eQTL signal. Both SNPs lie within the same intron of *ERBB*4 as the candidate rs7588550 and its Affymetrix 6.0 proxies in the 1000 Genomes CEU dataset. In the Pima cohort, the SNP rs17418640 is in LD D’=1 with one proxy (the other failed QC), but weakly related by r^2^.

To infer a likely functional role for the effect of these SNPs within early DN, genes with significant correlated expression were identified (Pearson’s r correlation). A total of 388 *ERBB4*-correlated genes were found in the DN biopsy expression profiles (Benjamini-Hochberg Q-value <0.1), and, of these, 12 are also putatively associated with the *ERBB4* SNPs in *trans* (*P*≤0.05). Table S5 shows significantly enriched pathways (GePS, Genomatix) for the *ERBB4*-correlated genes in early DN.

**Cell culture:** Human kidney epithelial cells (HK-2) were purchased from the European Tissue Culture Collection (ETCC). HK-2 cells were cultured at 37 °C in a humidified atmosphere of 95% air/5% CO_2_, and maintained in DMEM-F12 (Sigma) supplemented with 2 mM l-glutamine, 100 U ml^-1^ penicillin, 100 µg ml^-1^ streptomycin, 10 ng ml^-1^ EGF, 36 ng ml^-1^ hydrocortisone, 3 pg ml^-1^ triiodothyronine and 5 µg ml^-1^ insulin– 5 µg ml^-1^ transferrin– 5ng ml^-1^ selenium (ITS) solution (Sigma-Aldrich). Cell stimulations were performed with TGF-β1 (5 ng ml^-1^ PromoCell) or vehicle for 48 h.

**Western blot and qRT-PCR:** Protein lysates were harvested in RIPA lysis buffer containing 50 mM Tris-HCl, pH 7.4, 1% Nonidet P-40, 0.25% sodium deoxycholate, 150 mM NaCl, and 1 mM EDTA, supplemented with 1 mM phenylmethylsulfonyl fluoride, 1 mM sodium orthovanadate, 1mM sodium fluoride and a protease inhibitor cocktail (pepstatin 1.0 µg ml^-1^, leupeptin 1.0 µg ml^-1^, bestatin 1.0 µg ml^-1^, and aprotinin 1.0 µg ml^-1^) (Sigma-Aldrich). Cell lysates were centrifuged at 20,000 g for 15 min at 4°C and total protein was estimated using the Bradford assay (BioRad). For Western blot analysis, normalized protein extract was resolved by SDS-PAGE. Proteins were then transferred onto Immobilin P-transfer membranes (Millipore), blocked with TBS-T (25 mM Tris•HCl, pH 7.6, 150 mM NaCl, and 1.0% (v/v) Tween 20) supplemented with 5% (w/v) non-fat dried milk and then incubated with the following antibodies: β-actin (1:20,000; Sigma-Aldrich), AFF3 (1:1,000; Santa Cruz) CTGF (1:2,000; Santa-Cruz), E-cadherin, (1:1,000; BD Biosciences), N-cadherin, (1:1,000; BD Biosciences) and Jagged1 (1:2,000; Santa-Cruz). Membranes were subsequently incubated with horseradish peroxidase-linked secondary antibodies (New England Biolabs). Blots were developed using enhanced chemiluminescence reagents (Supersignal™). Total RNA extraction was performed using an RNeasy RNA extraction kit according to the manufacturer’s protocol (Qiagen). RNA quality was assessed using a Bioanalyzer 2100 (Agilent). cDNA was synthesized from 1 µg of total RNA using the Superscript-II RNase H-Reverse transcriptase cDNA synthesis kit (Invitrogen). Real-Time TaqMan PCR was used to quantify relative gene expression levels of CDH1, CDH2, CTGF, JAG1 and AFF3 from total RNA extracted from HK-2 cells. The PCR primers and TaqMan probe for all assays were supplied as a pre-optimized single tube primer/probe Gene Expression Assay (Applied Biosystems). 18s rRNA was used as an endogenous control for normalization of the target genes. PCR reactions were set up with Taqman Universal PCR Master Mix in a 10 µl reaction using 1:20 diluted cDNA. Amplification was performed on the 7900HT Sequence Detection System (Applied Biosystems). Results were analyzed using the ΔCt method of analysis.

**AFF3 siRNA transfections of renal epithelial cells:** siGENOME SMARTpool AFF3 siRNA, and siGENOME RISC-Free control siRNA were purchased from Dharmacon. siRNAs were transfected into HK-2 cells at 60% confluence using Lipofectamine 2000 (Invitrogen) at a final concentration of 20 nM for 24 hr. Cells were then stimulated with TGF-β1 (5 ng ml^-1^ PromoCell) or vehicle for 48 h.

References

1. He B, Osterholm AM, Hoverfalt A, Forsblom C, Hjorleifsdottir EE et al. (2009) Association of genetic variants at 3q22 with nephropathy in patients with type 1 diabetes mellitus. Am J Hum Genet 84: 5-13.

2. McKnight AJ, Patterson CC, Pettigrew KA, Savage DA, Kilner J et al. (2010) A GREM1 gene variant associates with diabetic nephropathy. J Am Soc Nephrol 21: 773-781.

3. Pezzolesi MG, Poznik GD, Mychaleckyj JC, Paterson AD, Barati MT et al. (2009) Genome-wide association scan for diabetic nephropathy susceptibility genes in type 1 diabetes. Diabetes 58: 1403-1410.

4. Syreeni A, El Osta A, Forsblom C, Sandholm N, Parkkonen M et al. (2011) Genetic examination of SETD7 and SUV39H1/H2 methyltransferases and the risk of diabetes complications in patients with type 1 diabetes. Diabetes 60: 3073-3080.

5. McKnight AJ, Patterson CC, Pettigrew KA, Savage DA, Kilner J et al. (2010) A GREM1 gene variant associates with diabetic nephropathy. J Am Soc Nephrol 21: 773-781.

6. Bain SC, Gill GV, Dyer PH, Jones AF, Murphy M et al. (2003) Characteristics of Type 1 diabetes of over 50 years duration (the Golden Years Cohort). Diabet Med 20: 808-811.

7. 1986) The Diabetes Control and Complications Trial (DCCT). Design and methodologic considerations for the feasibility phase. The DCCT Research Group. Diabetes 35: 530-545.

8. Molitch ME, Steffes MW, Cleary PA, Nathan DM (1993) Baseline analysis of renal function in the Diabetes Control and Complications Trial. The Diabetes Control and Complications Trial Research Group [corrected]. Kidney Int 43: 668-674.

9. 1995) Effect of intensive therapy on the development and progression of diabetic nephropathy in the Diabetes Control and Complications Trial. The Diabetes Control and Complications (DCCT) Research Group. Kidney Int 47: 1703-1720.

10. EDIC (2003) Sustained effect of intensive treatment of type 1 diabetes mellitus on development and progression of diabetic nephropathy: the Epidemiology of Diabetes Interventions and Complications (EDIC) study. JAMA 290: 2159-2167.

11. Paterson AD, Waggott D, Boright AP, Hosseini SM, Shen E et al. (2010) A genome-wide association study identifies a novel major locus for glycemic control in type 1 diabetes, as measured by both A1C and glucose. Diabetes 59: 539-549.

12. Tarnow L, Groop PH, Hadjadj S, Kazeem G, Cambien F et al. (2008) European rational approach for the genetics of diabetic complications--EURAGEDIC: patient populations and strategy. Nephrol Dial Transplant 23: 161-168.

13. Bröchner-Mortensen J, Rödbro P (1976) Selection of routine method for determination of glomerular filtration rate in adult patients. Scand J Clin Lab Invest 36: 35-45.

14. * National Kidney Foundation (2002) K/DOQI clinical practice guidelines for chronic kidney disease: evaluation, classification, and stratification. Am J Kidney Dis 39: S1-266.

15. Lindholm E, Agardh E, Tuomi T, Groop L, Agardh CD (2001) Classifying diabetes according to the new WHO clinical stages. Eur J Epidemiol 17: 983-989.

16. Marre M, Jeunemaitre X, Gallois Y, Rodier M, Chatellier G et al. (1997) Contribution of genetic polymorphism in the renin-angiotensin system to the development of renal complications in insulin-dependent diabetes: Genetique de la Nephropathie Diabetique (GENEDIAB) study group. J Clin Invest 99: 1585-1595.

17. Hadjadj S, Pean F, Gallois Y, Passa P, Aubert R et al. (2004) Different patterns of insulin resistance in relatives of type 1 diabetic patients with retinopathy or nephropathy: the Genesis France-Belgium Study. Diabetes Care 27: 2661-2668.

18. Pluzhnikov A, Below JE, Konkashbaev A, Tikhomirov A, Kistner-Griffin E et al. (2010) Spoiling the whole bunch: quality control aimed at preserving the integrity of high-throughput genotyping. Am J Hum Genet 87: 123-128.

19. Purcell S, Neale B, Todd-Brown K, Thomas L, Ferreira MA et al. (2007) PLINK: a tool set for whole-genome association and population-based linkage analyses. Am J Hum Genet 81: 559-575.

20. Price AL, Patterson NJ, Plenge RM, Weinblatt ME, Shadick NA et al. (2006) Principal components analysis corrects for stratification in genome-wide association studies. Nat Genet 38: 904-909.

21. Purcell S, Neale B, Todd-Brown K, Thomas L, Ferreira MA et al. (2007) PLINK: a tool set for whole-genome association and population-based linkage analyses. Am J Hum Genet 81: 559-575.

22. Pezzolesi MG, Poznik GD, Mychaleckyj JC, Paterson AD, Barati MT et al. (2009) Genome-wide association scan for diabetic nephropathy susceptibility genes in type 1 diabetes. Diabetes 58: 1403-1410.

23. Willer CJ, Li Y, Abecasis GR (2010) METAL: fast and efficient meta-analysis of genomewide association scans. Bioinformatics 26: 2190-2191.

24. Purcell S, Cherny SS, Sham PC (2003) Genetic Power Calculator: design of linkage and association genetic mapping studies of complex traits. Bioinformatics 19: 149-150.

25. Pruim RJ, Welch RP, Sanna S, Teslovich TM, Chines PS et al. (2010) LocusZoom: regional visualization of genome-wide association scan results. Bioinformatics 26: 2336-2337.

26. Segre AV, Groop L, Mootha VK, Daly MJ, Altshuler D (2010) Common inherited variation in mitochondrial genes is not enriched for associations with type 2 diabetes or related glycemic traits. PLoS Genet 6.

27. Berthier CC, Zhang H, Schin M, Henger A, Nelson RG et al. (2009) Enhanced expression of Janus kinase-signal transducer and activator of transcription pathway members in human diabetic nephropathy. Diabetes 58: 469-477.

28. Malhotra A, Kobes S, Knowler WC, Baier LJ, Bogardus C et al. (2011) A genome-wide association study of BMI in American Indians. Obesity (Silver Spring) 19: 2102-2106.
